# Supplementary material for: Qualitative investigation of relatives’ and service users’ experience of mental healthcare for suicidal behaviour in bipolar disorder
Source: BMJ Open. 2019 Nov 11;9(11):e030335. doi: 10.1136/bmjopen-2019-030335 (PMC6858148; doi:10.1136/bmjopen-2019-030335)
Supplement: Supplementary data [file bmjopen-2019-030335supp001.pdf]

Supplementary Table S1: Account of the method used to identify themes, adapted from Braun and Clarke (2006).

<u>Familiarisation with data corpus.</u><br>Analyses started when two relatives and two self-harm participants' interviews had been completed and transcribed in full. Close reading of the transcripts by CC and SP enabled familiarisation with the data. Interviews and analysis were conducted in parallel and additional transcripts were incorporated into the analysis as they became available. Back-coding of older transcripts was carried out when new codes were identified.
<u>Generation of initial codes</u>.<br>Coding was conducted by CC and SP in order to confirm concordance of coding and ideas during analysis. Interesting features within the participants' accounts were highlighted and allocated preliminary codes. These coded extracts were compiled and organised within a working text document. Examples of early codes included: 'family fighting for additional care,' 'changes in personality not recorded by services,' 'hiding suicidal behaviour,' 'not wanting to be a burden to family.'
<u>Identification of themes</u>.<br>Through an iterative process of discussion and review CC and SP consolidated codes into potential themes by grouping coded extracts that represented the same or similar issues within the working document. While the accounts were initially analyses as a single group (of people with experience of suicidal behaviours in bipolar disorder) at this point attention was given to whether themes were consistent or discriminant between the accounts of the two participant populations (i.e. people with bipolar and suicidal behaviour disorder and people bereaved by the suicide of someone with bipolar disorder). Analysis continued until thematic saturation was achieved, and no new ideas relating to the research topic emerged.
<u>Review and definition of themes</u>.<br>Themes identified were summarised and presented for discussion with the extended multidisciplinary research team at team meetings. Themes were presented along with supporting quotes from across the participants group. Themes were accepted as valid once there was unanimous agreement across the research team that the theme was present and accurately captured the experiences of participants.
<u>Reporting the analysis.</u><br>Appropriate supporting quotes were selected from participants' account for inclusion in the report to illustrate each theme and associated subthemes. Models (Figures 1 and 2) were constructed to help describe the relationship between subthemes and aid understanding. Models were confirmed as appropriate via review and discussion with the research team. Results were then contextualized with reference to existing literature on care for suicidal behaviour in bipolar disorder.
