# Supplementary material for: Qualitative investigation of relatives’ and service users’ experience of mental healthcare for suicidal behaviour in bipolar disorder
Source: BMJ Open. 2019 Nov 11;9(11):e030335. doi: 10.1136/bmjopen-2019-030335 (PMC6858148; doi:10.1136/bmjopen-2019-030335)
Supplement: Supplementary data [file bmjopen-2019-030335supp002.pdf]

Supplementary Table S2: Full quotes relating to the theme 'access to care.'

Help-seeking
"He'd put himself in hospital, he hadn't been sectioned or anything, because he just felt so suicidal and depressed [r1]."
"I want to feel better, I don't want to be feeling low and you know, not happy [s6]."
Knowing how to navigate the system
"We didn't realise that we had a pass, if you like, back into the mental health system so we went back to the GP [r6]."
"I went to see the doctor and she said to me, right […] I am going to get someone to ring you from the crisis team, and you need somebody to talk to. Do you know how long it took? They never phoned me five weeks. I suffered like that for five weeks. It took five weeks, and in the end I have to phone up, and then they had no recognition of who I was [s7]."
"[T]he GP had phoned up the psychiatrist's secretary as well, and had got exactly the same. It was ring five numbers on a piece of paper, she said phone them so I phoned them, and they gave me another number so I phoned them, and they gave me another number so I phoned them, and they gave me another number, and it went round in a circle, so in the end the fifth person gave me the number of the first person to phone so I had been round in a ring […]and my GP got given the same ring of numbers and went round the same way [s5]"
"I said can I short circuit it by paying for it and she sort of said 'I'm not allowed to recommend anyone.' So it was this, I'm trying to do the best for [the patient] and you won't help me [r2]."
Gate-keepers
"I cannot fault my GP, because they […] could not have dealt with it any better. He was wonderful, and got us into the psychiatric hospital as soon, the same day, which was just unbelievable [r3]."
"I have to say the GPs were very much, 'oh pull yourself together,' you know, sort of attitude [r6]."

“I spent 3 days in and out of A&E begging them to help me [s2].”
“I was as honest as I am being with you, and [psychiatric liaison clinician] just turned to [psychiatric liaison nurse] and he went, ‘do you believe a word she’s saying?’ And this woman just looked at him and went, ‘no’ and I felt about that big, and I’m sort of thinking I need help… help [s1]”
“Nobody else would see me, so there was another psychiatrist at the hospital, they wouldn't see me because I was under the care of [consultant psychiatrist]. So they wouldn't touch me, so... it just shows how you can fall out of the system really [s5].”
Obtaining a correct diagnosis
“[T]hey put me on antidepressants and I went really, really manic for seven months or something, and I ended up just leaving. Oh, at the hospital I got arrested because I threw a bin through a window. And they had me arrested and then refused to treat me, which is where I was getting the treatment. So I was just left on these antidepressants [without a mood stabiliser] *going higher and higher [s5].”*
Increased risk due to being denied care
“The crisis team, the home treatment team, nobody would see me, and that is, that is when this [suicidal behaviour] happened, really I was just really desperate [s5].
“And also I present always with a mask and I think that's why I was diagnosed so late. You know because I am always, and I find with services they get quite confused you know they look at me and say, ‘You are not depressed, you are bathed and clothed.’ I am terribly depressed even though I am bathed and clothed [s3].”
“It used to make him angry, cross, you know, and he couldn’t get people to sort of listen, and to help him [r8].”
