# Supplementary material for: Qualitative investigation of relatives’ and service users’ experience of mental healthcare for suicidal behaviour in bipolar disorder
Source: BMJ Open. 2019 Nov 11;9(11):e030335. doi: 10.1136/bmjopen-2019-030335 (PMC6858148; doi:10.1136/bmjopen-2019-030335)
Supplement: Supplementary data [file bmjopen-2019-030335supp003.pdf]

**Supplementary Table S3:** Full quotes supporting the theme "problems with communication".

| Service users |
|---|
| *"She was putting up this front that she was absolutely fine, and that was the really big message that was coming across [r5]."* |
| *"I didn't want a professional to know. It's classic hiding it and not telling anybody, because they have to intervene [s2]."* |
| *"I had a suicide plan in place, or whatever they call it, and wouldn't reveal the details of that to him* [the psychiatrist] *[s5]"* |
| *"I used to get quite cross because, you know, he would say he hadn't seen this person [community psychiatric nurse], but when I inquired into it, the more likely thing was that they had gone to his house and he wasn't there [r8]."* |
| *"She had attempted it maybe about four times, but she didn't want us to know, she didn't want to worry us [r7]."* |
| *"I have got like a really good family, but I find it difficult to let people know a lot of stuff, they do know, but I keep a lot to myself and I don't know why I just, I just feel as though if I say things I will upset people [s6]."* |
| *"I came down and found him in the chair. He took an overdose and he'd promised [the psychiatrist] that he wasn't suicidal. He said that he was alright now things were getting done [r1]."* |
| Relatives |
| *"[W]e were aware that he was drinking. I brought all this, about a month before [the patient] died, I brought all this to the attention of his CPN […] and he was quite shocked by that, and the living conditions, how [the patient] was living [r4]."* |
| *"I had been delusional for weeks and weeks and I was really poorly, and my daughter was ringing the mental health service saying you need to come and check my mum she is not, my* |

| |
|---|
| *mum is not well she is acting in a weird way, she is walking round naked, she is not good, you need to come you know, and they didn't come [s4].”* |
| *“[A]s a family we were their biggest resource and they never sort of came to us to help. Either to help us, or to use what we were saying to help him [r4].”* |
| *“I feel so sorry for anybody who had got depression or anything like that got no chance unless you have got somebody to help you [s7].”* |
| *“I would like somebody to come here and sit them all down and tell them what it involves because I don't think they have a clue [s7].”* |
| *“I have written an advanced statement, because you know the services would be so grateful to have family involved that they push the care to the family, where really that is detrimental to me [s3]”* |
| *“You just got told on many, many occasions that, you know, its patient confidentiality. But, when you’re dealing with somebody who’s suffering from mental health, and if I turn round and said to them, I said “if my son had cancer, you would have involved me, to care for him quite well at home [r4].”* |
| *“[W]hen he died, you know, the psychiatrist had actually said that he didn't think it was planned suicide, because he didn't think [the patient] was in that frame of mind to make that decision at that time, even though, even though he had been telling me that he wanted, you know, he didn't want to live. He wanted to die for probably months [r6].”* |
| *“[W]hen he died, you know, the psychiatrist had actually said that he didn't think it was planned suicide, because he didn't think [the patient] was in that frame of mind to make that decision at that time, even though, even though he had been telling me that he wanted, you know, he didn't want to live. He wanted to die for probably months [r6].”* |
| Mental health teams |
| *“[T]hey never said to me, you know your mom has mental illness you might need to you know go and get yourself checked out at the doctors, with your suicidal thoughts, suicidal tendencies... they didn't do it, they didn't do it [s4].”* |

| |
|---|
| *"[I]n the appointment that I had, after I had self-harmed and run away, […] one of the home treatment team came into the appointment and sat in the room with me, and neither she nor the psychiatrist mentioned the fact that I had self-harmed and run away [s5]."* |
| *"[W]hen I spoke to [community psychiatric nurse] on the Thursday after me thinking about suicide on the Wednesday, she spoke to the psychiatrist and then she spoke to the crisis team, and she came out on the Friday and brought the crisis team out on the Friday and said, and they said if I needed them over the weekend that they could you know, I could ring them and they would come and see me. So you have a bigger support work now than you did a few years ago, because there wasn't no crisis team a few years ago [s4]."* |
| *"I gave up* [on] *the psychiatrist because they were a bloody waste of time, they didn't know me. They didn't know who I was. They just read the notes and there was no connection at all [s7]."* |
