# Supplementary material for: Qualitative investigation of relatives’ and service users’ experience of mental healthcare for suicidal behaviour in bipolar disorder
Source: BMJ Open. 2019 Nov 11;9(11):e030335. doi: 10.1136/bmjopen-2019-030335 (PMC6858148; doi:10.1136/bmjopen-2019-030335)
Supplement: Author's manuscript [file bmjopen-2019-030335.draft_revisions.pdf]

BMJ Open

BMJ Open is committed to open peer review. As part of this commitment we make the peer review history of every article we publish publicly available.

When an article is published we post the peer reviewers' comments and the authors' responses online. We also post the versions of the paper that were used during peer review. These are the versions that the peer review comments apply to.

The versions of the paper that follow are the versions that were submitted during the peer review process. They are not the versions of record or the final published versions. They should not be cited or distributed as the published version of this manuscript.

BMJ Open is an open access journal and the full, final, typeset and author-corrected version of record of the manuscript is available on our site with no access controls, subscription charges or pay-per-view fees (http://bmjopen.bmj.com).

If you have any questions on BMJ Open's open peer review process please email info.bmjopen@bmj.com

BMJ Open

A qualitative investigation of relatives' and patients' experience of mental health care for suicidal behaviour in bipolar disorder.

Journal:	*BMJ Open*
Manuscript ID	bmjopen-2019-030335
Article Type:	Research
Date Submitted by the Author:	13-Mar-2019

Complete List of Authors:	Clements, Caroline; University of Manchester Faculty of Biology, Medicine and Health, Centre for Mental Health and Safety<br>Kapur, Navneet; University of Manchester Faculty of Biology, Medicine and Health, Centre for Mental Health and Safety<br>Jones, Steven; Lancaster University Faculty of Health and Medicine<br>Morriss, Richard; University of Nottingham, Psychiatry<br>Peters, Sarah; University of Manchester Faculty of Biology, Medicine and Health, Psychology and Mental Health

Keywords:	suicide, self-harm, bipolar disorder, QUALITATIVE RESEARCH

SCHOLARONE™
Manuscripts

A qualitative investigation of relatives' and patients' experience of mental health care for suicidal behaviour in bipolar disorder.

Caroline Clements[1], N. Kapur[1&5], Steve Jones[2], Richard Morriss[3], and Sarah Peters[4]

[1]Centre for Mental Health and Safety, Division of Psychology and Mental Health, School of Health Sciences, University of Manchester, Manchester Academic Health Sciences Centre, UK.

[2]Spectrum Centre for Mental Health Research, School of Health and Medicine, Lancaster University, UK.

[3]Division of Psychiatry and Applied Psychology, School of Medicine, University of Nottingham, UK.

[4]Division of Psychology and Mental Health, School of Health Sciences, University of Manchester, UK.

[5]Greater Manchester Mental Health NHS Foundation Trust, Manchester, UK.

Corresponding Author:
Caroline Clements
Room 2.308, Jean McFarlane Building,
Centre for Mental Health and Safety,
The University of Manchester,
Oxford Road,
Manchester,
M13 9PL
UK
Fax: +44(0)161 275 0716
Email: caroline.v.clements@manchester.ac.uk

Word Count: 4746

Key Words: Suicide, self-harm, bipolar disorder, qualitative, relatives

For peer review only

ABSTRACT

Objective: People with bipolar disorder are known to be at high risk of engaging in suicidal behaviours, and those who die by suicide have often been in recent contact with mental health services. The objective of this study was to explore suicidal behaviour in bipolar disorder and how suicidal behaviours are monitored and managed in this high risk group.

Aims: To identify themes within relatives' and patients' accounts of mental health care, related to management and prevention of suicidal behaviour in bipolar disorder.

Design: Thematic analysis of 22 semi-structured interviews.

Participants: Participants were people aged 18 years or over, fluent in written and spoken English, who either, 1) had bipolar disorder and a history of suicidal behaviour, or 2) were relatives of people who had bipolar disorder and died by suicide.

Setting: England, UK.

Primary outcome: Themes identified from participants' accounts of mental health care for suicidal behaviours in bipolar disorder.

Results: Two main themes were identified. 'The merry-go-round of access to mental health care' was characterised as a cyclical journey where potential barriers, such as gate-keepers, lack of an accurate diagnosis and lack of knowledge, prevented access care, with implications for increased risk of suicidal behaviours when participants became trapped in the cycle. 'Routes and roadblocks in communication' captured the importance of maintaining open routes of communication between all parties involved in care to ensure successful monitoring and management of suicidal behaviours in bipolar disorder.

Conclusions: Mental health services need to be accessible and respond rapidly to people with suicidal behaviour in bipolar disorder. Open communication and inclusion of relatives in care, where appropriate, could help closer monitoring of changes in symptoms that indicate increased risk.

Article Summary: Strengths and limitations of the study.

- This was a novel qualitative exploration of suicidal behaviour in bipolar disorder.
- Thematic analysis of in-depth semi-structured interviews generated rich and complex participant-driven data that offered new insight into why suicidal risk in people with bipolar disorder might be missed by mental health services.
- This study is exploratory in nature, and further work is needed to confirm which themes/subthemes are specific to people with bipolar disorder.

For peer review only - http://bmjopen.bmj.com/site/about/guidelines.xhtml

For peer review only

INTRODUCTION

Bipolar disorder is a common mental illness with an estimated prevalence over 2.4% worldwide [1]. People with bipolar disorder are not only subject to potential burdens and reduced quality of life across a number of domains (e.g. employment, social, physical health etc.), but are known to be at high risk of engaging in suicidal behaviours [2,3] . More than half of people diagnosed with bipolar disorder self-harm at least once in their life-time, and 6-7% die by suicide with risk estimated at 20-30 times that of the general population [3–7]. Some authors suggest that failure to identify elevated risk of suicidal behaviours in people with bipolar disorder may reflect the nature of the disorder, with rapid changes in mood-state alongside impulsive characteristics, leading to limitations in assessing diagnosis-specific risk [8,9].

Potential difficulties in assessing suicide risk in this population was illustrated in a UK study of people with bipolar disorder who died by suicide while under the care of mental health services [10]. Sixty percent had been seen by the care team within seven days of the suicide, with 40% seen within 24 hours of the suicide [10]. Furthermore, most people with bipolar disorder who present to hospital for self-harm report being currently under the care of community mental health services [11]. These results suggest that risk is not being appropriately identified or responded to within this group and that opportunities for intervention are being missed.

It may be that treatments shown to be effective at reducing suicidal behaviour, such as cognitive-behavioural therapies or medication with lithium are not being successfully implemented by mental health services in response to suicidal crises in people with bipolar disorder [12–16]. However, work with other psychiatric groups and general samples have revealed difficulties in access to and/or engagement with mental health services in relation to suicidal behaviours [17].

A number of studies have investigated barriers and facilitators of access to/engagement with mental health and medical services in general patient groups [18–20], psychiatric groups [17], and after episodes of suicidal behaviour [21–23]. Although qualitative methods have been used to explore the broader experiences of people with bipolar disorder (and their carers), such as social factors in suicidality [24], psychiatric assessment [25] and other aspects of mental health care [26–28], there has been no investigation specific to accessing care and support for suicidal behaviours in this psychiatric group. Understanding the experiences of this high-risk group is a priority, particularly with regard to how mental health services respond to people with bipolar disorder at times of crisis and increased risk. The aim of this study was, therefore, to explore relatives' and patients'

For peer review only - http://bmjopen.bmj.com/site/about/guidelines.xhtml

experience of mental health services in relation to suicidal behaviour in bipolar disorder to exploring why suicidal risk in this population might be missed.

METHOD

Participants

Participants were: 1) relatives/carers of people with bipolar disorder who died by suicide, or 2) people with bipolar disorder who had self-harmed (self-harm is defined here as any self-poisoning or self-injury without reference to motivation or level of suicidal intent [29]). To ensure capture of a broad range of experiences there were no further inclusion criteria beyond being over 18 years of age, and being fluent in written and spoken English (use of translators was beyond the scope of this study and fluency in English was essential enable fully informed consent). As the target population for this study was small and highly specific, to maximise recruitment no time-limits were placed on when the suicidal behaviour had taken place.

Given the sensitivity of the topic and potential for burden and distress, no formal assessments were conducted to establish a diagnosis of bipolar disorder. Inclusion was based on self-reported diagnosis, or diagnosis as reported by relative participants (the terms ‘relatives’ and ‘family members’ are used throughout the report rather than ‘carers’ as all were first-degree relatives of the deceased individual). The interview did include information on diagnostic history and therefore it was possible to confirm that most individuals had received a formal diagnosis of bipolar disorder (n=19), while the rest (n=3) had a provisional or suspected diagnosis made via a mental health professional prior to death (all self-harm participants had a formal diagnosis). The interview participants with bipolar disorder had experiences of a range of self-harm behaviours; from medically minor self-injury to serious suicide attempts. Participants also reported changes in method of self-harm, severity and intent over time, which is consistent with studies of hospital presentations for self-harm [30].

Ethical approval for the study was obtained from the National Research Ethics Service (Ref: 10/H1003/84) and R&D approvals were granted from all trusts involved in recruitment.

Recruitment

Twenty-two people were recruited into the study, split equally between the two participant groups.

For peer review only - http://bmjopen.bmj.com/site/about/guidelines.xhtml

Recruitment from highly specified populations was challenging and a variety of pragmatic methods were used. Recruitment initially centred on the North West area of England, but by necessity was rolled out to include all of England (amendment approved by the National Research Ethics Service to reflect these changes). Identification of potential participants was done using the following methods; identification of people who had presented to hospital following self-harm via local psychiatric liaison services in Manchester, UK; identification and referral via clinical studies officers based within the local NHS mental health trust; advertising via mental health charities and local newspapers; in-person visits to local voluntary sector support groups e.g. Bipolar UK; as well as personal referrals from other participants and research colleagues.

Potential participants were given a detailed participant information sheet and an opportunity to discuss the study further with the research team, before giving written informed consent to participate in the interview. Details of the study and procedure were repeated at the time of the interview and consent re-confirmed verbally.

Interviews

Semi-structured interviews were conducted, using a topic guide to facilitate the conversation. The topic guide was based on factors relevant to suicidal behaviour in bipolar disorder from the existing research literature, and input from a service users. Topics included; history of diagnosis, changes in mood or behaviour immediately before the most recent suicidal behaviour, situational factors such as negative life events or stressors, experience of mental health care, social support, and whether opportunities to prevent the suicidal behaviour had been missed. As this work was exploratory in nature, participants were encouraged to tell the story of their own experiences, and to raise any additional issues they felt were important.

Participants were interviewed in-person at the participant's home (n=10), or by telephone (n=12), ensuring the participant had appropriate levels of comfort and privacy in which to talk. Interviews were audio-recorded and transcribed verbatim. A code was allocated to each interview and identifying information (e.g. names and places) was removed to maintain anonymity. Interview duration ranged from 30 and 90 minutes, with a mean duration of 1 hour.

Patient and Public involvement

This work was carried out as part of the PARADES programme of research into bipolar disorder. A service user reference group for the programme had input into the development of the project from

For peer review only

an early stage. The methodology, recruitment materials, and topic guide were reviewed by the group and amendments made in line with feedback. The interview was also piloted with a service user and amended based on feedback provided.

Analysis

Thematic analysis was used on the interview data [31]. As this method is unattached to a specific theoretical framework and, offers flexibility to explore and interpret detailed qualitative data, it is particularly suited to pragmatic and exploratory work like that undertaken here. See supplementary Table S1 for full details of the analysis.

For peer review only

RESULTS

A summary of participants characteristics are provided in Table 1.

TABLE ONE HERE

Two main themes were identified, each containing multiple related subthemes. Given the opportunity to direct the conversation, participants' accounts coalesced around issues relating to access to mental health services, and failures in communication between parties involved in care, which were seen as contributing to poor care or lack of intervention when risk of suicidal behaviour was high. Themes are described below and, for purposes of brevity, illustrated by abbreviated quotes (presented in *italics*), from relatives [r] or service users [s]. Full versions of the quotations and additional supporting quotes are provided in Supplementary Table S2 and Supplementary Table S3.

The "*merry-go-round*" of access to mental health care.

Participants reported a range of difficulties when trying access to mental health services at times when they/their relative were at risk of engaging in suicidal behaviours. Issues raised applied across multiple service-contact situations, including; people currently receiving outpatient care, people discharged or disengaged from services, and people accessing care for the first time. The title of this theme derived from the observation of a service user that trying to access help for suicidal behaviour in bipolar disorder was *"like a merry-go-round [s2]."* This title captures the cyclical nature of as illustrated in the conceptual model in Figure 1.

Participants described difficult paths to receipt of mental health care for suicidal behaviours involving delays in obtaining an accurate diagnosis, increasing illness severity, suicidal thoughts and behaviours, poor experiences with GPs and/or clinical staff; ultimately leading to frustration and feelings of helplessness and the potential for further suicidal behaviours. Barriers to care were evident throughout the data corpus, but some participants did report positive experiences, with access to mental health services and support for prevention of suicidal behaviours facilitated. Therefore, the barriers discussed here only have the potential to interrupt or block access to successful mental health care for suicidal behaviour in bipolar disorder. Successful contact with mental health services has the potential to break the cyclical pattern at any stage.

FIGURE 1 HERE

Help-Seeking: Most participants/relatives of participants (i.e. the individual with bipolar disorder) did seek help for suicidal behaviour. Times of increased risk were recognised, as indicated by elevated suicidal thoughts and behaviours, and support from health/mental health services was actively sought.

> *"He'd put himself in hospital, he hadn't been sectioned or anything, because he just felt so suicidal and depressed [r1]."*

Navigating the system: Once on the path to accessing care, participants reported difficulty navigating their way to mental health care appropriate for them as people with both bipolar disorder and suicidal behaviours. There was extensive confusion over what services were available, which would provide the right support, and how to initiate contact. At times of suicidal crisis when help was needed urgently having the correct knowledge of where for effective support was seen as vital—but services often failed to respond promptly.

> *"We didn't realise that we had a pass [...] back into the mental health system so we went back to the GP [r6]."*

> *"I went to see the doctor and she said [...] I am going to get someone to ring you from the crisis team [...]. Do you know how long it took? They never phoned me five weeks. I suffered like that for five weeks. [s7]."*

In some circumstances confusion over access to appropriate services to medical professionals, such as GPs who participants turned to for urgent help with their suicidal crisis.

> *"[T]he GP had phoned up the psychiatrist's secretary as well, and had got exactly the same. It was ring five numbers on a piece of paper, she said phone them so I phoned them, and they*

For peer review only - http://bmjopen.bmj.com/site/about/guidelines.xhtml

gave me another number so I phoned them [...] and it went round in a circle, so in the end the fifth person gave me the number of the first person […] and my GP got given the same ring of numbers and went round the same way [s5].”

Another facet of this subtheme was accessing services either unavailable on the NHS or only available via long waiting-lists. Where participant/relatives were willing and able (e.g. financially) to tap into additional sources of support outside of the NHS, the value and appreciation of the expertise within NHS care teams was reflected in a desire for guidance and signposting to potentially helpful services, such as additional talking therapies. . In practice this type of guidance was limited or outright refused, leaving patients and relatives frustrated.

“I said can I short circuit it by paying for it and she sort of said ‘I’m not allowed to recommend anyone.’ So it was this, I’m trying to do the best for [the patient] and you won’t help me [r2].”

Gate-keepers: First point of contact was often the GP, who then acted as a default gate-keeper to accessing secondary mental health services. In this role GPs were viewed either as allies:

“I cannot fault my GP, because they […] could not have dealt with it any better [r3].”

Or as another barrier to be overcome, either through poor attitude or a lack of knowledge of how to respond to people with bipolar disorder experiencing suicidal thoughts and behaviours.

“I have to say the GPs were very much, ‘oh pull yourself together,’ you know, sort of attitude [r6].”

Hospital emergency departments were another critical point of contact, where clinical staff are placed in the role of gatekeeper to further mental health care. As in work on people who attend hospital for suicidal behaviour in general, participants/relatives with bipolar disorder were often subjected to negative attitudes of medical staff. Problems with psychiatric liaison staff as gatekeepers was a less common but not rare, with failures to recognise bipolar disorder symptomatology and associated risks of suicidal behaviours preventing access to further care.

“I was as honest as I am being with you, and [psychiatric liaison clinician] just turned to [psychiatric liaison nurse] and he went, ‘do you believe a word she’s saying?’ And this woman just looked at him and went, ‘no’ and I felt about that big, and I’m sort of thinking I need help… help [s1].”

While the quotes above describe first attempts to access mental health care, gate-keepers were also a potential barrier to ongoing successful care for participants/relatives already in contact with secondary mental health services. For example, when the psychiatrist responsible for a patient’s care is unavailable:

For peer review only - http://bmjopen.bmj.com/site/about/guidelines.xhtml

"[S]o there was another psychiatrist at the hospital, they wouldn't see me because I was under the care of [consultant psychiatrist]. So they wouldn't touch me [s5]."

Accurate diagnosis: A common experience for participants was the failure of clinicians (and here there is some cross-over with gate-keepers in the reasons given for barring participants from accessing further care services) failure to identify or recognise a diagnosis of bipolar disorder, and thereby failing to acknowledge associated increased risks. Lack of a formal diagnosis related to difficulties accessing care, and in gaining appropriate care from mental health services. This increased the risk of inappropriate or inadequate medication, exacerbated symptoms and increased potential for suicidal behaviours.

"[T]hey put me on antidepressants and I went really, really manic for seven months or something [...]. So I was just left on these antidepressants going higher and higher [s5]."

Screaming for help: The final subtheme in the "*merry-go-round*" of access to mental health care encapsulates participants' increasing frustration at failing to overcome the preceding barriers. Furthermore, participants were clear that this cycle, and repeated failures to access suitable care, can precipitate further suicidal behaviours; leaving participants/relatives feeling they have to *"scream for help [s2]."*

"The crisis team, the home treatment team, nobody would see me, and that is, that is when this [suicidal behaviour] happened, really I was just really desperate [s5].

A facet of this subtheme was a sense of distress at not being seen as ill enough to warrant mental health care. This was particularly evident when participants looked well or were able to articulate their own concerns about their illness and the risk of engaging in suicidal behaviours.

"I find with services they get quite confused you know they look at me and say, 'You are not depressed, you are bathed and clothed.' I am terribly depressed even though I am bathed and clothed [s3]."

Routes and roadblocks in communication.

Drawing together participants' experiences under the care of mental health services, the theme 'routes and roadblocks in communication' describes the importance of clear communication in the ongoing care of people with bipolar disorder, especially at times of crisis when suicidal behaviour is likely. Paths of communication are between any parties with an interest in the care of a patient, and are here presented in relation to the patient, relatives, and the mental health team. A conceptual

For peer review only - http://bmjopen.bmj.com/site/about/guidelines.xhtml

model is presented in Figure 2 which shows how potential roadblocks can disrupt the flow if information between parties.

FIGURE TWO HERE

Evaluation of care: Participants' judgements of quality of care provided by mental health services were more positive where care was seen as inclusive via taking relatives' and patients' opinions and wishes into account. When care was collaborative and fostered good communication between parties, opinions on quality of care received were positive regardless of outcome.

> *"[T]there was one guy, he was one of the few [health care professionals] that both [the patient] and I trusted because he was quite happy to have us both in at the same time [r2]."*

Sympathy and trust were key features of positive evaluations of care received. Clinicians who were willing to provide and receive information from patients and relatives were valued. Continuity of care to establish long-term therapeutic relationships were vital for creating trust, and allowed clinicians greater understanding of individual patients' illness, behaviour, and situation; aiding monitoring of bipolar disorder symptoms and identification of times of increased risk of suicidal behaviour. Where these factors were lacking, participants evaluated care as poor.

> *"I gave up [on] the psychiatrist because they were a bloody waste of time, they didn't know me. […] They just read the notes and there was no connection at all [s7]."*

Patients' communication: Patients were the main source of information about changes in illness, behaviour, and risk of suicidal behaviour, for clinicians and relatives. Some participants acknowledged that suicidal intent remained undisclosed to others at the time. Lack of awareness of changes in symptoms and the presence of non-fatal suicidal behaviours therefore remained hidden from relatives and services and opportunities to intervene were denied.

> *"I didn't want a professional to know. It's classic hiding it and not telling anybody, because they have to intervene [s2]."*

Patients' failure of disclosure of information that might help manage suicidal behaviours in bipolar disorder was not always intentional. Memory problems and disorganised lifestyles were also given as reasons why important changes may not have been reported.

> *"I used to get quite cross […], he would say he hadn't seen [his community psychiatric nurse], but when I inquired into it, the more likely thing was that they had gone to his house and he wasn't there [r8]."*

For peer review only - http://bmjopen.bmj.com/site/about/guidelines.xhtml

Motivation for failing to communicate suicidal thoughts and behaviours to relatives was driven largely by the wish to protect others from distress and to avoid placing additional burden on relatives.

> *"[S]he didn't want to worry us [r7]."*

> *"I keep a lot to myself and I don't know why I just, I just feel as though if I say things I will upset people [s6]."*

Whatever the reasons, the tangible consequence was that relatives and/or the mental health team were unaware of increasing risk and unable to respond effectively.

> *"I came down and found him in the chair. He took an overdose and he'd promised [the psychiatrist] that he wasn't suicidal. [r1]."*

Relatives' communication: The involvement of relatives in mental health care was generally positive. Proximity to the patient helped with observation of changes in illness and behaviour, and increased awareness of situational factors, such as the presence of stressful or negative life events, that might increase the risk of suicidal behaviour.

> *"[W]e were aware that he was drinking. […] I brought all this to the attention of his CPN […] and he was quite shocked by that, and the living conditions, how [the patient] was living [r4]."*

Relatives saw themselves as an untapped resource for services, and wanted to be considered and treated as full partners in the care of the patient.

> *"[A]s a family we were their biggest resource and they never sort of came to us to help. Either to help us, or to use what we were saying to help him [r4]."*

However, not all participants felt supported by relatives, or wanted the support of relatives. In some situations there was resistance on the part of the family to being involved in care. Lack of knowledge of bipolar disorder and the associated risks of suicidal behaviour were cited as obstacles. Where family involvement was wanted, a role was seen for mental health services in engagement and education of relatives to improve support and safety for the patient at home.

> *"I would like somebody to come here and sit them all down and tell them what it involves because I don't think they have a clue [s7]."*

Conversely, where familial relationships were poor or difficult the involvement of family members was seen as potentially detrimental to the patient and their ongoing care.

> *"[T]the services would be so grateful to have family involved that they push the care to the family, where really that is detrimental to me [s3]"*

For peer review only - http://bmjopen.bmj.com/site/about/guidelines.xhtml

Participants also gave accounts of conflict between family members and mental health staff. This was particularly evident when relatives felt prevented from contributing to care—which often went hand-in-hand with confusion over patient confidentiality rules.

> *"You just got told on many, many occasions that, you know, its patient confidentiality. […] I turn round and said to them, I said "if my son had cancer, you would have involved me, to care for him quite well at home [r4]."*

This roadblock led to failures in communicating information with potential value for assessing risk of suicidal behaviours in people with bipolar disorder. Relatives expressed frustration at being unable to help the patient despite being in possession of knowledge that may have influenced care.

> *"[W]hen he died, you know, the psychiatrist had actually said that he didn't think it was planned suicide, […] even though he had been telling me that he wanted, you know, he didn't want to live. He wanted to die for probably months [r6]."*

Mental Health Services' communication: Participants gave multiple accounts of communication failures between patients and the care team. Poor explanations of diagnosis and prognosis, risks and triggers, medication regimens and possible side effects of treatments were examples of times where participants felt ignored or dismissed by clinical teams. The result was poorer management, weaker therapeutic relationships, and ultimately the potential to miss indicators of increased risk.

> *"[T]hey never said to me, you know your mom has mental illness you might need to you know go and get yourself checked out at the doctors, with your suicidal thoughts, suicidal tendencies... [s4]."*

Following suicidal behaviour some participants reported being left without any follow-up or acknowledgment from clinical teams. Subsequently there was no adjustment of treatment or changes in monitoring in response to the suicidal behaviour.

> *"[O]ne of the home treatment team came into the appointment and sat in the room with me, and neither she nor the psychiatrist mentioned the fact that I had self-harmed and run away [s5]."*

However, there was some evidence that communication between services and patients has improved over time. With accounts of new teams and initiatives created to better ensure the safety of patients, participants were hopeful that services will continue to improve in the future.

> *"[S]he came out on the Friday and brought the crisis team out […] they said if I needed them over the weekend that they could you know, I could ring them and they would come and see me. So you have a bigger support work now than you did a few years ago [s4]."*

For peer review only - http://bmjopen.bmj.com/site/about/guidelines.xhtml

For peer review only

DISCUSSION

Main findings

This study used a systematic qualitative approach to investigate experiences of mental health care in relation to suicidal behaviour in bipolar disorder. Two main themes captured the focus of participants' accounts; potential barriers to accessing mental health services, and potential blocks in routes of communication between patients, relatives, and services, both of which might contribute to failures to identify and respond to indicators of increased suicide risk within this group.

A number of the experiences discussed in relation to the themes are common among psychiatric groups in general [19,20]. And while this does not diminish the importance of these concerns, their impact on the individual, or the possibility these difficulties may be over-represented among people with suicidal behaviour in bipolar disorder, this discussion will focus on factors that may be specific to the identification and prevention of suicidal behaviours in bipolar disorder.

Active help-seeking was common among participants. There was no evidence of issues relating to stigma or other individual-centred barriers as documented in more general studies of people with bipolar disorder [19,20,32]. Instead, barriers were predominantly external, such as gate-keeping and poor staff attitudes to suicidal behaviours [33,34]. One area of gate-keeping discussed by participants that may be particularly relevant to this group was the incorrect assumption of low illness severity and risk based on appearance and the ability to effectively communicate safety concerns. Bipolar disorder is characterised by periods of illness and relative stability when patients may be better able to effectively recognise and communicate distress and risk [3]. Although the literature is inconclusive there is some evidence that, compared to other psychiatric groups, people with bipolar disorder have an educational attainment level similar to the general population [35,36]. With unaffected pre-morbid intellectual functioning and an average age of onset of 25 years—possibly allowing for engagement in higher education, as found in our sample—it may be that this group tend towards more effective communication skills, especially when describing risk of suicidal behaviours[35–37]. Ensuring accurate diagnosis was critical in the management of suicidal behaviours in this group. Compared to some other psychiatric diagnoses, an accurate diagnosis of bipolar disorder is commonly delayed due to misdiagnosis of initial symptoms – especially when symptoms of depression are present, [38]. This aligns with evidence of the under-recognition of bipolar disorder among care services [39,40].

For peer review only - http://bmjopen.bmj.com/site/about/guidelines.xhtml

Mental health care for suicidal behaviour was enhanced by involving patients and relatives (where appropriate) in care decisions and resulted in positive evaluations of care even after the suicide of a loved-one. This is consistent with the Department of Health's consensus statement on sharing information with family and carers for the purpose of suicide prevention [41]. Therapies involving family members (e.g. family-focused therapy, psychoeducation) shown to help reduce relapse, symptom severity, and caregiver burden, may also be effective in addressing some of the communication blocks relating to suicidal behaviours found in this study (ref). This may be especially true of therapies that seek to educate families by increasing knowledge bipolar disorder and the associated increased risk [42,43]. Previous qualitative work showed that people with serious mental illness struggle to be seen as competent and equal partners in care decisions [44] – and this was evident in accounts given here where the focus was specifically on help for suicidal behaviours. Our work also supports the idea that relatives and carers often feel excluded from mental health care [28]. As in general mental health care for people with bipolar disorder, care received for suicidal behaviour was viewed more positively across interviews when care was collaborative and clinicians were sympathetic, open, and interested in involving patients and relatives [45].

Methodological limitations

Total number of participants was small but sufficient to provide a novel insight into the experiences of a small and hard to reach population--especially with regard to relatives—via rich in-depth data. Relatives may have inherently different perspectives on mental health care, their role, and concerns, following bereavement by suicide, than patients. As relatives were a more homogeneous group the commonalities of these accounts may have had greater influence on resulting themes. Although this does not reduce the validity of the themes presented, efforts were made throughout the results to give supporting quotes from both participant groups. Themes and conceptual models presented in this work do not attempt to give an exhaustive account of all experiences of accessing mental health care by people with suicidal behaviours in bipolar disorder. However, identification factors considered important by participants with diverse perspectives and experiences have been described, and common difficulties faced by this high-risk group when trying to access care identified.

Clinical implications

Many people who experience suicidal behaviour in bipolar disorder are in close proximity to care services at the time of the act. While the influence of rapidly changing mood-states and impulsivity make prediction and prevention of suicidal behaviours in this group particularly difficult, people with

For peer review only - http://bmjopen.bmj.com/site/about/guidelines.xhtml

experience of suicidal behaviours in bipolar disorder may have a role in helping to improve the response of mental health services [46–48]. Rapid access to care is crucial, and patients and relatives need good knowledge of what services are available to them, with clearly signposted ways to access services. Relatives are often in a position to observe behavioural changes that may indicate increased or fluctuating risk, and where possible, should be kept informed of treatment/management plans. Closer involvement of family and friends may allow important information to be communicated more quickly, help build trust and supportive therapeutic relationships, and create more positive judgements of overall quality of care provided by mental health services [49]. Further work with a wider range of participants, particularly those from less educated backgrounds where additional barriers may include health literacy, is needed to explore the generalisability of the themes presented, as well as how they could be translated into improved care practices.

Author contributions: NK and CC designed the study with input from all authors. CC collected all data. CC and SP analysed and interpreted the data, with input from all authors. CC drafted the manuscript. NK, SP, RM, SJ provided further input on subsequent drafts of the manuscript.

Funding: This article presents independent research commissioned by the National Institute for Health Research (NIHR) under its Programme Grants for Applied Research funding scheme (RP-PG-0407-10389). The views expressed in this work are those of the authors and not those of the NHS, NIHR, or Department of Health.

Competing interests: None

Patient consent for publication: Not required

Data statement: Due to confidentiality full interview data cannot be made available.

For peer review only

REFERENCES

1 Merikangas KR, Jin R, He J, *et al.* Prevalence and correlates of bipolar spectrum disorder in the world mental health survey initiative. *Arch Gen Psychiatry* 2011;**68**:241–51. doi:10.1001/archgenpsychiatry.2011.12.Prevalence

2 Michalak EE, Murray G, Collaborative RESearch Team to Study Psychosocial Issues in Bipolar Disorder (CREST.BD). Development of the QoL.BD: a disorder-specific scale to assess quality of life in bipolar disorder. *Bipolar Disord* 2010;**12**:727–40. doi:10.1111/j.1399-5618.2010.00865.x

3 Goodwin FK, Jamison KR. *Manic-Depressive Illness:Bipolar Disorders and Recurrent Depression: Bipolar Disorders and Recurrent Depression*. Oxford University Press, USA 2007. http://books.google.co.uk/books?id=X7XLhltqsv8C

4 Schaffer A, Isometsä ET, Tondo L, *et al.* International Society for Bipolar Disorders Task Force on Suicide: Meta-analyses and meta-regression of correlates of suicide attempts and suicide deaths in bipolar disorder. *Bipolar Disord* 2015;**17**:1–16. doi:10.1111/bdi.12271

5 Tondo L, Pompili M, Forte A, *et al.* Suicide attempts in bipolar disorders: Comprehensive review of 101 reports. *Acta Psychiatr Scand* 2016;**133**:174–86. doi:10.1111/acps.12517

6 Pompili M, Gonda X, Serafini G, *et al.* Epidemiology of suicide in bipolar disorders: a systematic review of the literature. *Bipolar Disord* 2013;**15**:457–90. doi:10.1111/bdi.12087

7 Nordentoft M, Mortensen PBB, Pedersen CB. Absolute risk of suicide after first hospital contact in mental disorder. *Arch Gen Psychiatry* 2011;**68**:1058–64. doi:10.1001/archgenpsychiatry.2011.113

8 Watkins HB, Meyer TD. Is there an empirical link between impulsivity and suicidality in bipolar disorders? A review of the current literature and the potential psychological implications of the relationship. *Bipolar Disord* 2013;**15**:542–58. doi:10.1111/bdi.12090

9 Gonda X, Pompili M, Serafini G, *et al.* Suicidal behavior in bipolar disorder: epidemiology, characteristics and major risk factors. *J Affect Disord* 2012;**143**:16–26. doi:10.1016/j.jad.2012.04.041

10 Clements C, Morriss R, Jones S, *et al.* Suicide in bipolar disorder in a national English sample, 1996-2009: Frequency, trends and characteristics. *Psychol Med* 2013;**43**. doi:10.1017/S0033291713000329

For peer review only - http://bmjopen.bmj.com/site/about/guidelines.xhtml

11 Clements C, Jones S, Morriss R, *et al.* Self-harm in bipolar disorder: Findings from a prospective clinical database. *J Affect Disord* 2015;**173**:113–9. doi:10.1016/j.jad.2014.10.012

12 Smith KA, Cipriani A. Lithium and suicide in mood disorders: Updated meta-review of the scientific literature. *Bipolar Disord* 2017;**19**:575–86. doi:10.1111/bdi.12543

13 Baldessarini RJ, Tondo L, Vázquez GH. Pharmacological treatment of adult bipolar disorder. *Mol Psychiatry* 2018;:1–20. doi:10.1038/s41380-018-0044-2

14 Tarrier N, Taylor K, Gooding P. Cognitive-behavioral interventions to reduce suicide behavior: a systematic review and meta-analysis. *Behav Modif* 2008;**32**:77–108. doi:10.1177/0145445507304728

15 Rihmer Z, Gonda X, Tondo L. Lithium and suicidal behavior in patients with bipolar disorder. *J Clin Psychiatry* 2008;**69**:1831–2.http://www.ncbi.nlm.nih.gov/pubmed/19200432 (accessed 12 May 2014).

16 Cipriani A, Hawton K, Stockton S, *et al.* Lithium in the prevention of suicide in mood disorders: updated systematic review and meta-analysis. *BMJ* 2013;**3646**:1–13. doi:10.1136/bmj.f3646

17 Anderson KK, Fuhrer R, Malla AK. “There are too many steps before you get to where you need to be”: Help-seeking by patients with first-episode psychosis. *J Ment Heal* 2012;**22**:384–95. doi:10.3109/09638237.2012.705922

18 Smith TE, Easter A, Pollock M, *et al.* Disengagement From Care: Perspectives of Individuals With Serious Mental Illness and of Service Providers. *Psychiatr Serv* 2013;**64**:770–5. doi:10.1176/appi.ps.201200394

19 Clement S, Schauman O, Graham T, *et al.* What is the impact of mental health-related stigma on help-seeking? A systematic review of quantitative and qualitative studies. *Psychol Med* 2015;**45**:11–27. doi:10.1017/S0033291714000129

20 Dockery L, Jeffery D, Schauman O, *et al.* Stigma- and non-stigma-related treatment barriers to mental healthcare reported by service users and caregivers. *Psychiatry Res* 2015;**228**:612–9. doi:10.1016/j.psychres.2015.05.044

21 Owens C, Lambert H, Donovan J, *et al.* A qualitative study of help seeking and primary care consultation prior to suicide. *Br J Gen Pract* 2005;**55**:503–9.

22 Taylor TL, Hawton K, Fortune S, *et al.* Attitudes towards clinical services among people who self-harm: Systematic review. *Br J Psychiatry* 2009;**194**:104–10.

For peer review only - http://bmjopen.bmj.com/site/about/guidelines.xhtml

doi:10.1192/bjp.bp.107.046425

23 Jordan J, McKenna H, Keeney S, *et al.* Providing meaningful care: learning from the experiences of suicidal young men. *Qual Heal Res* 2012;**22**:1207–19. doi:10.1177/1049732312450367

24 Owen R, Gooding P, Dempsey R, *et al.* A qualitative investigation into the relationships between social factors and suicidal thoughts and acts experienced by people with a bipolar disorder diagnosis. *J Affect Disord* 2015;**176**:133–40. doi:10.1016/j.jad.2015.02.002

25 Bilderbeck AC, Saunders KEA, Price J, *et al.* Psychiatric assessment of mood instability: qualitative study of patient experience. *Br J Psychiatry* 2014;**204**:234–9. doi:10.1192/bjp.bp.113.128348

26 Highet N, McNair B, Thompson M, *et al.* Experience with treatment services for people with bipolar disorder. *Med J Aust* 2004;**181**:47–51.https://www.mja.com.au/journal/2004/181/7/experience-treatment-services-people-bipolar-disorder

27 Chatzidamianos G, Lobban F, Jones S. A qualitative analysis of relatives', health professionals' and service users' views on the involvement in care of relatives in Bipolar Disorder. *BMC Psychiatry* 2015;**15**:228. doi:10.1186/s12888-015-0611-x

28 Cree L, Brooks HL, Berzins K, *et al.* Carers ' experiences of involvement in care planning : a qualitative exploration of the facilitators and barriers to engagement with mental health services. *BMC Psychiatry* 2015;:1–11. doi:10.1186/s12888-015-0590-y

29 Hawton K, Harriss L, Hall S, *et al.* Deliberate self-harm in Oxford, 1990-2000: a time of change in patient characteristics. *Psychol Med* 2003;**33**:987–95.http://www.ncbi.nlm.nih.gov/pubmed/12946083 (accessed 26 May 2014).

30 Owens D, Kelley R, Munyombwe T, *et al.* Switching methods of self-harm at repeat episodes: Findings from a multicentre cohort study. *J Affect Disord* 2015;**180**:44–51. doi:10.1016/j.jad.2015.03.051

31 Braun V, Clarke V. Using thematic analysis in psychology. *Qual Res Psychol* 2006;**3**:77–101. doi:10.1191/1478088706qp063oa

32 Hawke LD, Parikh S V., Michalak EE. Stigma and bipolar disorder: A review of the literature. *J Affect Disord* 2013;**150**:181–91. doi:10.1016/j.jad.2013.05.030

For peer review only - http://bmjopen.bmj.com/site/about/guidelines.xhtml

33 Anthony JS, Baik RNS, Bowers RNBJ, *et al.* Conditions That Influence a Primary Care Clinician ' s Decision to Refer Patients for Depression Care. *Rehabil Nurs* 2010;**35**:113–22.

34 Saunders KEA, Hawton K, Fortune S, *et al.* Attitudes and knowledge of clinical staff regarding people who self-harm: A systematic review. *J Affect Disord* 2012;**139**:205–16. doi:10.1016/j.jad.2011.08.024

35 Schoeyen HK, Melle I, Sundet K, *et al.* Occupational outcome in bipolar disorder is not predicted by premorbid functioning and intelligence. *Bipolar Disord* 2013;**15**:294–305. doi:10.1111/bdi.12056

36 Dickinson D, Harvey PD. Systemic hypotheses for generalized cognitive deficits in schizophrenia: a new take on an old problem. *Schizophr Bull* 2009;**35**:403–14. doi:10.1093/schbul/sbn097

37 Bauer M, Glenn T, Alda M, *et al.* Influence of birth cohort on age of onset cluster analysis in bipolar I disorder. *Eur Psychiatry* 2015;**30**:99–105. doi:10.1016/j.eurpsy.2014.10.005

38 Altamura AC, Buoli M, Caldiroli A, *et al.* Misdiagnosis, duration of untreated illness (DUI) and outcome in bipolar patients with psychotic symptoms: A naturalistic study. *J Affect Disord* 2015;**182**:70–5. doi:10.1016/j.jad.2015.04.024

39 Hughes T, Cardno A, West R, *et al.* Unrecognised bipolar disorder among UK primary care patients prescribed antidepressants: an observational study. *Br J Gen Pract* 2016;**66**:e71–7. doi:10.3399/bjgp16X683437

40 Mosolov S, Ushkalova A, Kostukova E, *et al.* Bipolar II disorder in patients with a current diagnosis of recurrent depression. *Bipolar Disord* 2014;**16**:389–99. doi:10.1111/bdi.12192

41 Department of Health. Information Sharing and Suicide Prevention: Consensus Statement. 2014. https://www.gov.uk/government/uploads/system/uploads/attachment_data/file/271792/Consensus_statement_on_information_sharing.pdf

42 Miklowitz DJ, Chung B. Family-Focused Therapy for Bipolar Disorder: Reflections on 30 Years of Research. *Fam Process* 2016;**55**:483–99. doi:10.1111/famp.12237

43 Baruch E, Pistrang N, Barker C. Psychological interventions for caregivers of people with bipolar disorder: A systematic review and meta-analysis. *J Affect Disord* 2018;**236**:187–98. doi:10.1016/j.jad.2018.04.077

For peer review only

44 Dahlqvist Jönsson P, Schön U-K, Rosenberg D, *et al.* Service users' experiences of participation in decision making in mental health services. *J Psychiatr Ment Health Nurs* 2015;**22**:688–97. doi:10.1111/jpm.12246

45 Norcross JC, Wampold BE. Evidence-based therapy relationships: research conclusions and clinical practices. *Psychotherapy (Chic)* 2011;**48**:98–102. doi:10.1037/a0022161

46 Cassidy F. Risk factors of attempted suicide in bipolar disorder. *Suicide Life Threat Behav* 2011;**41**:6–11. doi:10.1111/j.1943-278X.2010.00007.x

47 D'Ambrosio V, Salvi V, Bogetto F, *et al.* Serum lipids, metabolic syndrome and lifetime suicide attempts in patients with bipolar disorder. *Prog Neuropsychopharmacol Biol Psychiatry* 2012;**37**:136–40. doi:10.1016/j.pnpbp.2011.12.009

48 Tondo L, Baldessarini R. Suicidal risk in bipolar disorder. *Clin Neuropsychiatry* 2005;**2**:55–65.

49 Dirik A, Sandhu S, Giacco D, *et al.* Why involve families in acute mental healthcare? A collaborative conceptual review. *BMJ Open* 2017;**7**:1–10. doi:10.1136/bmjopen-2017-017680

For peer review only

For peer review only - http://bmjopen.bmj.com/site/about/guidelines.xhtml

Table 1. Demographic breakdown of interview participants

Variable	Participants n=22(%)
**Sex**	
Male	4 (18.2)
Female	18 (81.8)
**Age group**	
18-35	4 (18.2)
36-50	10 (45.5)
51-65	4 (18.2)
66>	4 (18.2)
**Method***	
Overdose	9 (45.0)
Self-injury	8 (40.0)
Hanging	3 (15.0)
**Time since last act****	
< 1 year	8 (44.4)
1-3 years	7 (38.9)
4-6 years	2 (11.1)
7 years >	1 (5.6)

*method unknown for 2 cases, denominator n=20
**Exact time since last act unknown in 4 cases, denominator n=18

For peer review only

Figure 1. Conceptual model of the theme 'the ‘merry-go-round’ of access to mental health care' based on service users and relatives experiences of care for suicidal behaviours in bipolar disorder.

Figure 2. Conceptual model of the theme 'routes and roadblocks in communication' based on service users and relatives experiences of care for suicidal behaviours in bipolar disorder.

For peer review only

For peer review only - http://bmjopen.bmj.com/site/about/guidelines.xhtml

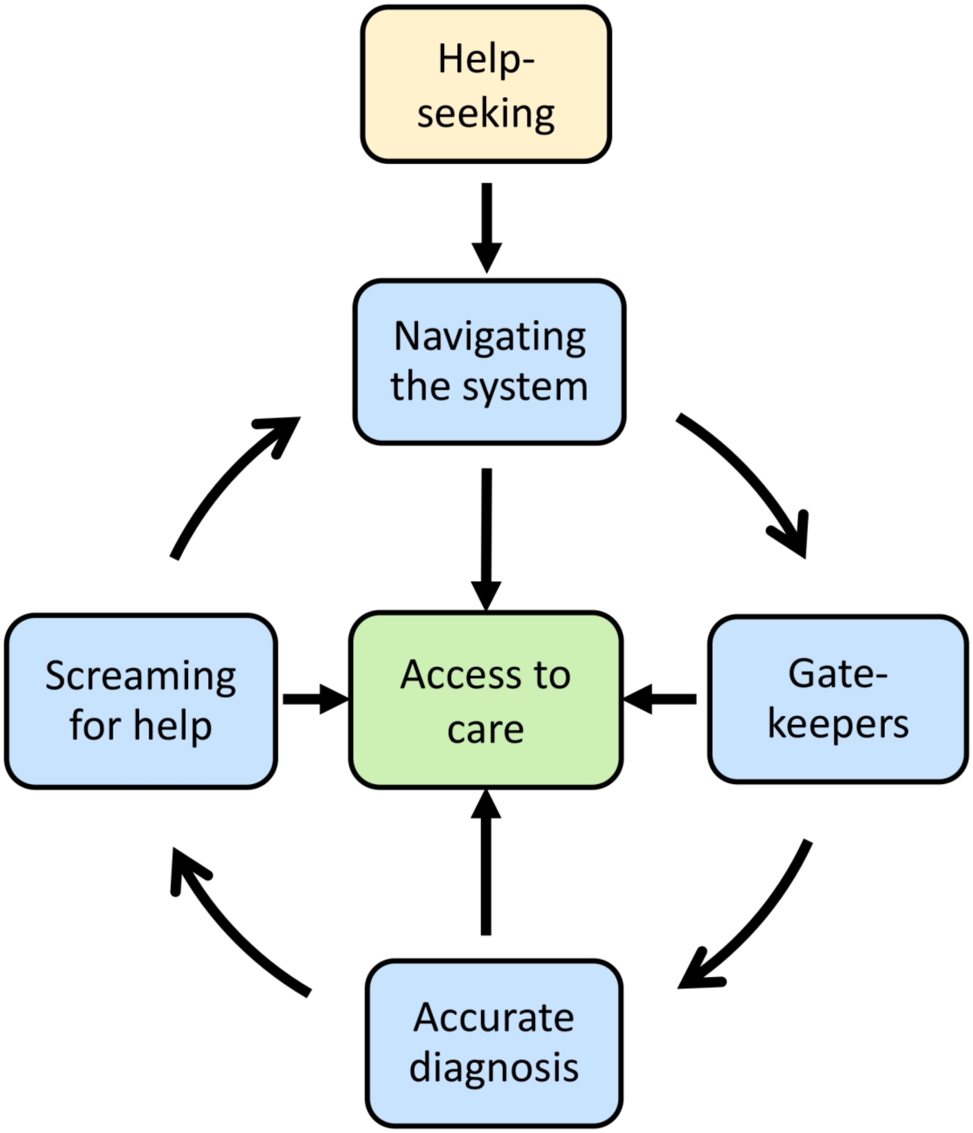

Figure 1. Conceptual model of the theme 'the ‘merry-go-round’ of access to mental health care' based on service users and relatives experiences of care for suicidal behaviours in bipolar disorder.

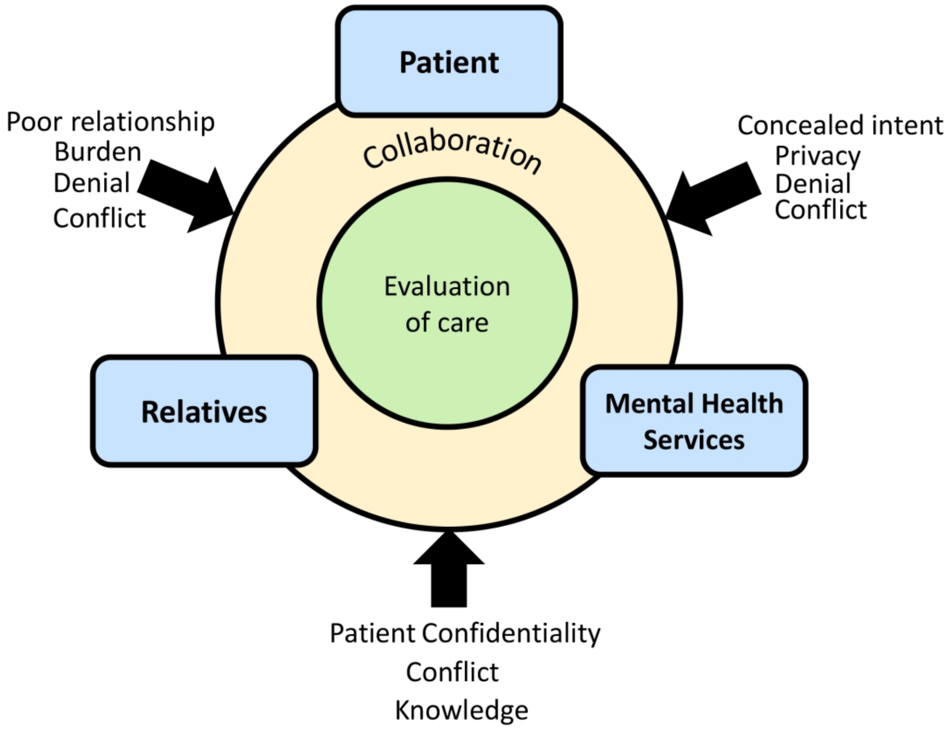

Figure 2. Conceptual model of the theme 'routes and roadblocks in communication' based on service users and relatives experiences of care for suicidal behaviours in bipolar disorder.

Supplementary Table S1: Account of the method used to identify themes, adapted from Braun and Clarke (2006).

Familiarisation with data corpus. Analyses started when two relatives and two self-harm participants' interviews had been completed and transcribed, with a close reading of all transcripts (CC). Analysis was carried out across the two participant groups, and transcripts were read by at least one other member of the research team (SP) to provide concordance of initial ideas and coding. Interviews and analysis were conducted in parallel and additional transcripts were included as they became available.
Generation of initial codes. Interesting features within the dataset were highlighted and given preliminary codes. Coded extracts were then compiled to create a working document.
Identification of themes. Through an iterative process of discussion and review, codes within the working document were consolidated into potential themes, with relevant codes and data extracts gathered together under the theme heading. At this point any irrelevant codes were removed.
Review and application of themes. The preliminary themes were then applied across the transcripts in a step-wise fashion, with any additional codes/themes being reapplied to earlier transcripts as the analysis progressed.
Defining themes. Through ongoing analysis the themes were refined and organized into a structure of higher-level and subthemes. This process was reviewed by the wider research team. Analysis continued until thematic saturation was achieved, and no new ideas relating to the research topic emerged.
Reporting the analysis. Appropriate supporting extracts were selected for inclusion in the report, and conceptual models (Figures 1 and 2) were constructed to illustrate the relationship of subthemes and higher-order themes. The results of the analyses were then contextualized by links to existing literature, and the research topic.

For peer review only

Supplementary Table S2: Full quotes relating to the theme 'the merry-go-round of access to mental health care.'

Help-seeking
"He'd put himself in hospital, he hadn't been sectioned or anything, because he just felt so suicidal and depressed [r1]."
"I want to feel better, I don't want to be feeling low and you know, not happy [s6]."
Navigating the system
"We didn't realise that we had a pass, if you like, back into the mental health system so we went back to the GP [r6]."
"I went to see the doctor and she said to me, right […] I am going to get someone to ring you from the crisis team, and you need somebody to talk to. Do you know how long it took? They never phoned me five weeks. I suffered like that for five weeks. It took five weeks, and in the end I have to phone up, and then they had no recognition of who I was [s7]."
"[T]he GP had phoned up the psychiatrist's secretary as well, and had got exactly the same. It was ring five numbers on a piece of paper, she said phone them so I phoned them, and they gave me another number so I phoned them, and they gave me another number so I phoned them, and they gave me another number, and it went round in a circle, so in the end the fifth person gave me the number of the first person to phone so I had been round in a ring […]and my GP got given the same ring of numbers and went round the same way [s5]"
"I said can I short circuit it by paying for it and she sort of said 'I'm not allowed to recommend anyone.' So it was this, I'm trying to do the best for [the patient] and you won't help me [r2]."
Gate-keepers

For peer review only

"I cannot fault my GP, because they […] could not have dealt with it any better. He was wonderful, and got us into the psychiatric hospital as soon, the same day, which was just unbelievable [r3]."
"I have to say the GPs were very much, 'oh pull yourself together,' you know, sort of attitude [r6]."
"I spent 3 days in and out of A&E begging them to help me [s2]."
"I was as honest as I am being with you, and [psychiatric liaison clinician] just turned to [psychiatric liaison nurse] and he went, 'do you believe a word she's saying?' And this woman just looked at him and went, 'no' and I felt about that big, and I'm sort of thinking I need help… help [s1]"
"Nobody else would see me, so there was another psychiatrist at the hospital, they wouldn't see me because I was under the care of [consultant psychiatrist]. So they wouldn't touch me, so... it just shows how you can fall out of the system really [s5]."
Formal diagnosis
"[T]hey put me on antidepressants and I went really, really manic for seven months or something, and I ended up just leaving. Oh, at the hospital I got arrested because I threw a bin through a window. And they had me arrested and then refused to treat me, which is where I was getting the treatment. So I was just left on these antidepressants [without a mood stabiliser] *going higher and higher [s5]."*
Screaming for help
"The crisis team, the home treatment team, nobody would see me, and that is, that is when this [suicidal behaviour] happened, really I was just really desperate [s5].
"And also I present always with a mask and I think that's why I was diagnosed so late. You

For peer review only

know because I am always, and I find with services they get quite confused you know they look at me and say , 'You are not depressed, you are bathed and clothed.' I am terribly depressed even though I am bathed and clothed [s3]."
"It used to make him angry, cross, you know, and he couldn't get people to sort of listen, and to help him [r8]."
"It used to make him angry, cross, you know, and he couldn't get people to sort of listen, and to help him [r8]."

For peer review only

Supplementary Table S3: Full quotes supporting the theme Routes and Road blocks in communication.

Evaluations of Care
"Most of them they excluded me […] there was one guy, he was one of the few people [health care professionals] *that both [the patient] and I trusted because he was quite happy to have us both in at the same time [r2]."*
"I gave up [on] *the psychiatrist because they were a bloody waste of time, they didn't know me. They didn't know who I was. They just read the notes and there was no connection at all [s7]."*
"I mean I can't remember how many different people, social workers have dealt with him. It would be sort of a few months and then they were having a baby, fair enough the woman would be having a baby, but you know it doesn't help the person you are working with [r8]."
Patients' communication
"She was putting up this front that she was absolutely fine, and that was the really big message that was coming across [r5]."
"I didn't want a professional to know. It's classic hiding it and not telling anybody, because they have to intervene [s2]."
"I had a suicide plan in place, or whatever they call it, and wouldn't reveal the details of that to him [the psychiatrist] *[s5]"*
"I used to get quite cross because, you know, he would say he hadn't seen this person [community psychiatric nurse], but when I inquired into it, the more likely thing was that they had gone to his house and he wasn't there [r8]."

For peer review only

"She had attempted it maybe about four times, but she didn't want us to know, she didn't want to worry us [r7]."
"I have got like a really good family, but I find it difficult to let people know a lot of stuff, they do know, but I keep a lot to myself and I don't know why I just, I just feel as though if I say things I will upset people [s6]."
"I came down and found him in the chair. He took an overdose and he'd promised [the psychiatrist] that he wasn't suicidal. He said that he was alright now things were getting done [r1]."
Relatives' Communication
"[W]e were aware that he was drinking. I brought all this, about a month before [the patient] died, I brought all this to the attention of his CPN […] and he was quite shocked by that, and the living conditions, how [the patient] was living [r4]."
"I had been delusional for weeks and weeks and I was really poorly, and my daughter was ringing the mental health service saying you need to come and check my mum she is not, my mum is not well she is acting in a weird way, she is walking round naked, she is not good, you need to come you know, and they didn't come [s4]."
"[A]s a family we were their biggest resource and they never sort of came to us to help. Either to help us, or to use what we were saying to help him [r4]."
"I feel so sorry for anybody who had got depression or anything like that got no chance unless you have got somebody to help you [s7]."

For peer review only

"I would like somebody to come here and sit them all down and tell them what it involves because I don't think they have a clue [s7]."
"I have written an advanced statement, because you know the services would be so grateful to have family involved that they push the care to the family, where really that is detrimental to me [s3]"
"You just got told on many, many occasions that, you know, its patient confidentiality. But, when you're dealing with somebody who's suffering from mental health, and if I turn round and said to them, I said "if my son had cancer, you would have involved me, to care for him quite well at home [r4]."
"[W]hen he died, you know, the psychiatrist had actually said that he didn't think it was planned suicide, because he didn't think [the patient] was in that frame of mind to make that decision at that time, even though, even though he had been telling me that he wanted, you know, he didn't want to live. He wanted to die for probably months [r6]."
"You just got told on many, many occasions that, you know, its patient confidentiality. But, when you're dealing with somebody who's suffering from mental health, and if I turn round and said to them, I said "if my son had cancer, you would have involved me, to care for him quite well at home [r4]."
"[W]hen he died, you know, the psychiatrist had actually said that he didn't think it was planned suicide, because he didn't think [the patient] was in that frame of mind to make that decision at that time, even though, even though he had been telling me that he wanted, you know, he didn't want to live. He wanted to die for probably months [r6]."
Services' communication.

For peer review only

"[T]hey never said to me, you know your mom has mental illness you might need to you know go and get yourself checked out at the doctors, with your suicidal thoughts, suicidal tendencies... they didn't do it, they didn't do it [s4]."
"[I]n the appointment that I had, after I had self-harmed and run away, [...] one of the home treatment team came into the appointment and sat in the room with me, and neither she nor the psychiatrist mentioned the fact that I had self-harmed and run away [s5]."
"[W]hen I spoke to [community psychiatric nurse] on the Thursday after me thinking about suicide on the Wednesday, she spoke to the psychiatrist and then she spoke to the crisis team, and she came out on the Friday and brought the crisis team out on the Friday and said, and they said if I needed them over the weekend that they could you know, I could ring them and they would come and see me. So you have a bigger support work now than you did a few years ago, because there wasn't no crisis team a few years ago [s4]."

For peer review only

Reporting checklist for qualitative study.

Based on the SRQR guidelines.

Instructions to authors

Complete this checklist by entering the page numbers from your manuscript where readers will find each of the items listed below.

Your article may not currently address all the items on the checklist. Please modify your text to include the missing information. If you are certain that an item does not apply, please write "n/a" and provide a short explanation.

Upload your completed checklist as an extra file when you submit to a journal.

In your methods section, say that you used the SRQR reporting guidelines, and cite them as:

O'Brien BC, Harris IB, Beckman TJ, Reed DA, Cook DA. Standards for reporting qualitative research: a synthesis of recommendations. Acad Med. 2014;89(9):1245-1251.

		Reporting Item	Page Number
	#1	Concise description of the nature and topic of the study identifying the study as qualitative or indicating the approach (e.g. ethnography, grounded theory) or data collection methods (e.g. interview, focus group) is recommended	2
	#2	Summary of the key elements of the study using the abstract format of the intended publication; typically includes background, purpose, methods, results and conclusions	2
Problem formulation	#3	Description and signifcance of the problem / phenomenon studied: review of relevant theory and empirical work; problem statement	3
Purpose or research question	#4	Purpose of the study and specific objectives or questions	3
Qualitative approach and research paradigm	#5	Qualitative approach (e.g. ethnography, grounded theory, case study, phenomenolgy, narrative research)	4

For peer review only

		and guiding theory if appropriate; identifying the research paradigm (e.g. postpositivist, constructivist / interpretivist) is also recommended; rationale. The rationale should briefly discuss the justification for choosing that theory, approach, method or technique rather than other options available; the assumptions and limitations implicit in those choices and how those choices influence study conclusions and transferability. As appropriate the rationale for several items might be discussed together.	
Researcher characteristics and reflexivity	#6	Researchers' characteristics that may influence the research, including personal attributes, qualifications / experience, relationship with participants, assumptions and / or presuppositions; potential or actual interaction between researchers' characteristics and the research questions, approach, methods, results and / or transferability	n/a
Context	#7	Setting / site and salient contextual factors; rationale	4
Sampling strategy	#8	How and why research participants, documents, or events were selected; criteria for deciding when no further sampling was necessary (e.g. sampling saturation); rationale	4
Ethical issues pertaining to human subjects	#9	Documentation of approval by an appropriate ethics review board and participant consent, or explanation for lack thereof; other confidentiality and data security issues	4
Data collection methods	#10	Types of data collected; details of data collection procedures including (as appropriate) start and stop dates of data collection and analysis, iterative process, triangulation of sources / methods, and modification of procedures in response to evolving study findings; rationale	4-5
Data collection instruments and technologies	#11	Description of instruments (e.g. interview guides, questionnaires) and devices (e.g. audio recorders) used for data collection; if / how the instruments(s) changed over the course of the study	4-5
Units of study	#12	Number and relevant characteristics of participants, documents, or events included in the study; level of	4

For peer review only

		participation (could be reported in results)	
Data processing	#13	Methods for processing data prior to and during analysis, including transcription, data entry, data management and security, verification of data integrity, data coding, and anonymisation / deidentification of excerpts	Table S1
Data analysis	#14	Process by which inferences, themes, etc. were identified and developed, including the researchers involved in data analysis; usually references a specific paradigm or approach; rationale	Table S1
Techniques to enhance trustworthiness	#15	Techniques to enhance trustworthiness and credibility of data analysis (e.g. member checking, audit trail, triangulation); rationale	Table S1
Syntheses and interpretation	#16	Main findings (e.g. interpretations, inferences, and themes); might include development of a theory or model, or integration with prior research or theory	6-13
Links to empirical data	#17	Evidence (e.g. quotes, field notes, text excerpts, photographs) to substantiate analytic findings	6-13<br>Tables S2 & S3
Intergration with prior work, implications, transferability and contribution(s) to the field	#18	Short summary of main findings; explanation of how findings and conclusions connect to, support, elaborate on, or challenge conclusions of earlier scholarship; discussion of scope of application / generalizability; identification of unique contributions(s) to scholarship in a discipline or field	12-13
Limitations	#19	Trustworthiness and limitations of findings	13
Conflicts of interest	#20	Potential sources of influence of perceived influence on study conduct and conclusions; how these were managed	14
Funding	#21	Sources of funding and other support; role of funders in data collection, interpretation and reporting	14

The SRQR checklist is distributed with permission of Wolters Kluwer © 2014 by the Association of American Medical Colleges. This checklist can be completed online using https://www.goodreports.org/, a tool made by the EQUATOR Network in collaboration with Penelope.ai

For peer review only

BMJ Open

A qualitative investigation of relatives' and patients' experience of mental health care for suicidal behaviour in bipolar disorder.

Journal:	*BMJ Open*
Manuscript ID	bmjopen-2019-030335.R1
Article Type:	Original research
Date Submitted by the Author:	06-Aug-2019
Complete List of Authors:	Clements, Caroline; University of Manchester Faculty of Biology, Medicine and Health, Centre for Mental Health and Safety<br>Kapur, Navneet; University of Manchester Faculty of Biology, Medicine and Health, Centre for Mental Health and Safety<br>Jones, Steven; Lancaster University Faculty of Health and Medicine<br>Morriss, Richard; University of Nottingham, Psychiatry<br>Peters, Sarah; University of Manchester Faculty of Biology, Medicine and Health, Psychology and Mental Health
**Primary Subject Heading**:	Qualitative research
Secondary Subject Heading:	Mental health
Keywords:	suicide, self-harm, bipolar disorder, QUALITATIVE RESEARCH

SCHOLARONE™
Manuscripts

A qualitative investigation of relatives' and patients' experience of mental health care for suicidal behaviour in bipolar disorder.

Caroline Clements[1], N. Kapur[1&5], Steve Jones[2], Richard Morriss[3], and Sarah Peters[4]

[1]Centre for Mental Health and Safety, Division of Psychology and Mental Health, School of Health Sciences, University of Manchester, Manchester Academic Health Sciences Centre, UK.

[2]Spectrum Centre for Mental Health Research, School of Health and Medicine, Lancaster University, UK.

[3]Division of Psychiatry and Applied Psychology, School of Medicine, University of Nottingham, UK.

[4]Division of Psychology and Mental Health, School of Health Sciences, University of Manchester, UK.

[5]Greater Manchester Mental Health NHS Foundation Trust, Manchester, UK.

Corresponding Author:
Caroline Clements
Room 2.308, Jean McFarlane Building,
Centre for Mental Health and Safety,
The University of Manchester,
Oxford Road,
Manchester,
M13 9PL
UK
Fax: +44(0)161 275 0716
Email: caroline.v.clements@manchester.ac.uk

Word Count: 4907

Key Words: Suicide, self-harm, bipolar disorder, qualitative, relatives

For peer review only

ABSTRACT

Objective: People with bipolar disorder are known to be at high risk of engaging in suicidal behaviours, and those who die by suicide have often been in recent contact with mental health services. The objective of this study was to explore suicidal behaviour in bipolar disorder and how suicidal behaviours are monitored and managed in this high risk group.

Aims: To identify themes within relatives' and patients' accounts of mental health care, related to management and prevention of suicidal behaviour in bipolar disorder.

Design: Thematic analysis of 22 semi-structured interviews.

Participants: Participants were people aged 18 years or over, fluent in written and spoken English, who either, 1) had bipolar disorder and a history of suicidal behaviour, or 2) were relatives of people who had bipolar disorder and died by suicide.

Setting: England, UK.

Primary outcome: Themes identified from participants' accounts of mental health care for suicidal behaviours in bipolar disorder.

Results: Two main themes were identified. 'Access to care' was characterised by potential barriers, such as gate-keepers and lack of an accurate diagnosis prevented access care, with implications for increased risk of suicidal behaviours when participants became trapped in a cycle of failure to access care and increasing risk. 'Problems with communication' captured the importance of maintaining open routes of communication between all parties involved in care to ensure successful monitoring and management of suicidal behaviours in bipolar disorder.

Conclusions: Mental health services need to be accessible and respond rapidly to people with suicidal behaviour in bipolar disorder. Open communication and inclusion of relatives in care, where appropriate, could help closer monitoring of changes in symptoms that indicate increased risk.

For peer review only

For peer review only - http://bmjopen.bmj.com/site/about/guidelines.xhtml

Article Summary: Strengths and limitations of the study.

- This was a novel qualitative exploration of suicidal behaviour in bipolar disorder.
- Thematic analysis of in-depth semi-structured interviews generated rich and complex participant-driven data that offered new insight into why suicidal risk in people with bipolar disorder might be missed by mental health services.
- This study is exploratory in nature, and further work is needed to confirm which themes/subthemes are specific to people with bipolar disorder.

For peer review only

For peer review only - http://bmjopen.bmj.com/site/about/guidelines.xhtml

INTRODUCTION

Bipolar disorder is a common mental illness with an estimated prevalence over 2.4% worldwide [1]. People with bipolar disorder are not only subject to potential burdens and reduced quality of life across a number of domains (e.g. employment, social, physical health etc.), but are known to be at high risk of engaging in suicidal behaviours [2,3] . More than half of people diagnosed with bipolar disorder self-harm at least once in their life-time, and 6-7% die by suicide with risk estimated at 20-30 times that of the general population [3–7]. Some authors suggest that failure to identify elevated risk of suicidal behaviours in people with bipolar disorder may reflect the nature of the disorder, with rapid changes in mood-state alongside impulsive characteristics, leading to limitations in assessing diagnosis-specific risk [8,9].

Potential difficulties in assessing suicide risk in this population was illustrated in a UK study of people with bipolar disorder who died by suicide while under the care of mental health services [10]. Sixty percent had been seen by the care team within seven days of the suicide, with 40% seen within 24 hours of the suicide [10]. Furthermore, most people with bipolar disorder who present to hospital for self-harm report being currently under the care of community mental health services [11]. These results suggest that risk is not being appropriately identified or responded to within this group and that opportunities for intervention are being missed.

It may be that treatments shown to be effective at reducing suicidal behaviour, such as cognitive-behavioural therapies or medication with lithium are not being successfully implemented by mental health services in response to suicidal crises in people with bipolar disorder [12–16]. However, work with other psychiatric groups and general samples have revealed difficulties in access to and/or engagement with mental health services in relation to suicidal behaviours [17].

A number of studies have investigated barriers and facilitators of access to/engagement with mental health and medical services in general patient groups [18–20], psychiatric groups [17], and after episodes of suicidal behaviour [21–23]. Qualitative methods have been used to explore broader experiences of people with bipolar disorder (and their carers), such as social factors in suicidality [24], psychiatric assessment [25] and other aspects of mental health care [26–28]. For example a recent study by Vallarino et al (2019) explored the experiences of mental health care in people with bipolar disorder; timely provision of information about bipolar disorder, recovery-focused treatment, provision of psychosocial therapies, and the access to peer support, had an impact on the attitudes to and level of engagement with care services [29]. To date there has been no investigation specific to accessing care and support for suicidal behaviours in this psychiatric group.

For peer review only - http://bmjopen.bmj.com/site/about/guidelines.xhtml

Understanding the experiences of this high-risk group is a priority, particularly with regard to how mental health services respond to people with bipolar disorder at times of crisis and increased risk. The aim of this study was, therefore, to explore relatives' and patients' experience of mental health services in relation to suicidal behaviour in bipolar disorder to exploring why suicidal risk in this population might be missed.

METHOD

Participants

Participants were: 1) relatives/carers of people with bipolar disorder who died by suicide, or 2) people with bipolar disorder who had self-harmed (self-harm is defined here as any self-poisoning or self-injury without reference to motivation or level of suicidal intent [30]). To ensure capture of a broad range of experiences there were no further inclusion criteria beyond being over 18 years of age, and being fluent in written and spoken English (use of translators was beyond the scope of this study and fluency in English was essential enable fully informed consent). As the target population for this study was small and highly specific, to maximise recruitment no time-limits were placed on when the suicidal behaviour had taken place.

Two populations were included in order to encompass the full range of suicidal behaviour in bipolar disorder. While people with bipolar disorder who have experience of self-harm can speak to their own experiences, excluding information around those who died by suicide would neglect the experiences of a group who may have the highest need for intervention by mental health services. People bereaved by suicide are often included as informants in suicide research as they are well positioned to know details of the individual and circumstances leading to suicide unavailable from clinical records [31,32]. Although some significant events may be unknown to the bereaved person (as well as being subject to recall bias and the influence of distress on over/under emphasis of salient factors) it is recognised as a pragmatic approach.

Given the sensitivity of the topic and potential for burden and distress, no formal assessments were conducted to establish a diagnosis of bipolar disorder. Inclusion was based on self-reported diagnosis, or diagnosis as reported by relative participants (the terms 'relatives' and 'family members' are used throughout the report rather than 'carers' as all were first-degree relatives of the deceased individual). The interview did include information on diagnostic history and therefore it was possible to confirm that most individuals had received a formal diagnosis of bipolar disorder

For peer review only - http://bmjopen.bmj.com/site/about/guidelines.xhtml

(n=19), while the rest (n=3) had a provisional or suspected diagnosis made via a mental health professional prior to death (all self-harm participants had a formal diagnosis). The interview participants with bipolar disorder had experiences of a range of self-harm behaviours; from medically minor self-injury to serious suicide attempts. Participants also reported changes in method of self-harm, severity and intent over time, which is consistent with studies of hospital presentations for self-harm [33].

Reflexivity

Throughout the planning and conduct of the study there was awareness that the authors are experts in the field of self-harm and suicide research. The authors' prior practical and theoretical knowledge of existing models of suicide and self-harm as well as the clinical management of suicidal behaviours in mental health services made it essential to ensure that participants voices were genuinely represented. The authors attempted to critically examine their work at every stage of conduct and analysis to ensure that the resulting themes and conclusions were represented in the participants' accounts. This was accomplished in two ways; a) review and amendment of the topic schedule by an independent service user group and a semi-structured approach to interview that encouraged participants to direct the conversation and raise additional factors not included in the topic guide and, b) at every stage of the analysis authors checked identified themes against manuscripts to confirm they were actually present in participants' accounts and that themes were not unduly shaped by the researchers prior positions.

Ethical approval for the study was obtained from the National Research Ethics Service (Ref: 10/H1003/84) and R&D approvals were granted from all trusts involved in recruitment.

Recruitment

Twenty-two people were recruited into the study, split equally between the two participant groups.

Recruitment from highly specified populations was challenging and a variety of pragmatic methods were used. Recruitment initially centred on the North West area of England, but by necessity was rolled out to include all of England (amendment approved by the National Research Ethics Service to reflect these changes). Identification of potential participants was done using the following methods; identification of people who had presented to hospital following self-harm via local psychiatric liaison services in Manchester, UK; identification and referral via clinical studies officers based within the local NHS mental health trust; advertising via mental health charities and local newspapers; in-

For peer review only - http://bmjopen.bmj.com/site/about/guidelines.xhtml

person visits to local voluntary sector support groups e.g. Bipolar UK; as well as personal referrals from other participants and research colleagues.

Potential participants were given a detailed participant information sheet and an opportunity to discuss the study further with the research team, before giving written informed consent to participate in the interview. Details of the study and procedure were repeated at the time of the interview and consent re-confirmed verbally.

Interviews

Semi-structured interviews were conducted, using a topic guide to facilitate the conversation. The topic guide was based on factors relevant to suicidal behaviour in bipolar disorder from the existing research literature.

Topics included; history of diagnosis, changes in mood or behaviour immediately before the most recent suicidal behaviour, situational factors such as negative life events or stressors, experience of mental health care, social support, and whether opportunities to prevent the suicidal behaviour had been missed. As this work was exploratory in nature, participants were encouraged to tell the story of their own experiences, and to raise any additional issues they felt were important. Two authors (SP and CC) developed the interview procedure with input from the other authors, and one (CC) conducted the interviews. An outline of the sorts of topics that might be discussed was presented at the start of the interview to ensure that the participant was comfortable going ahead with the interview, and the semi-structured nature of the interview was explained to ensure that participants knew they were in control of the conversation and could discuss any topics they thought important to the experience of suicidal behaviour in bipolar disorder.

Participants were interviewed in-person at the participant's home (n=10), or by telephone (n=12), ensuring the participant had appropriate levels of comfort and privacy in which to talk. Interviews were audio-recorded and transcribed verbatim. A code was allocated to each interview and identifying information (e.g. names and places) was removed to maintain anonymity. Interview duration ranged from 30 and 90 minutes, with a mean duration of 1 hour. The use of both interview methods was primarily a pragmatic decision to aid recruitment and thereby access a range of perspectives and experiences, there are potential advantages and disadvantages to using both telephone and face-to-face interviews. Interviews via telephone can add distance and detachment during a potentially sensitive interview and help people feel more comfortable disclosing personal information[34,35]; however, face-to-face methods are known to increase rapport in the interview setting and may help put participants at ease [36,37]. There was no discernible differences identified

For peer review only - http://bmjopen.bmj.com/site/about/guidelines.xhtml

in relation to topics covered in interviews or themes identified in the analysis, between the telephone and face-to-face interviewees.

Patient and Public involvement

This work was carried out as part of the PARADES programme of research into bipolar disorder. A service user reference group for the programme had input into the development of the project from an early stage. The methodology, recruitment materials, and topic guide were reviewed by the group and amendments made in line with feedback. . A first draft of the topic guide was confirmed to be generally suitable for the purpose of the project, but the group advised that more detail be collected on past history of self-harm and interaction with services prior to the most recent episode of suicidal behaviour to give additional context to participants' experiences. The interview was also piloted with a service user who confirmed the interview procedure and topic guide were comprehensive and appropriate for the research topic.

Analysis

Thematic analysis was used on the interview data [38]. As this method is unattached to a specific theoretical framework and, offers flexibility to explore and interpret detailed qualitative data, it is particularly suited to pragmatic and exploratory work like that undertaken here. In brief: While initial coding was conducted by one researcher (CC), transcripts were also read by another member of the research team with expertise in qualitative methods (SP) to confirm concordance of coding and ideas during analysis. Participants' accounts were given preliminary codes which were organised within a text document. An iterative process of discussion and review between the two researchers (CC and SP) consolidated codes into potential themes (similar codes were grouped in the working document). Themes identified were summarised and presented and discussed with the extended research team to further refine final themes (more detailed information is presented in Supplementary Table S1).

RESULTS

A summary of participants' age and gender are provided in Table 1.

TABLE ONE HERE

Given the opportunity to direct the conversation participants' accounts coalesced around two main themes; 'access to care' and 'problems with communication'. Themes and subthemes are presented

For peer review only - http://bmjopen.bmj.com/site/about/guidelines.xhtml

below. For the purpose of brevity abridged quotes (presented in *italics*) by relatives [r] and service users [s] are presented. Full quotations and additional supporting quotes are provided in Supplementary Table S2 and Supplementary Table S3.

1. Access to care.

Participants reported a range of difficulties when trying access to mental health services when risk of engaging in suicidal behaviour was high. This theme applied across different service-contact situations, such as outpatient care, people discharged or disengaged from services, as well as people trying to access care for the first time. Within this the subthemes 'help-seeking,' 'knowing how to navigate the system,' 'gatekeepers,' 'obtaining a correct diagnosis', and 'increased risk due to being denied care,' formed a series of potential barriers to accessing successful mental health care for suicidal behaviours in bipolar disorder.

Help-Seeking: Most participants or relatives of participants (i.e. the individual with bipolar disorder) did try to seek help for suicidal behaviour. Especially at times of increased risk, when there were increases in the number or severity of suicidal thoughts and behaviours support from health/mental health services was actively sought.

> *"He'd put himself in hospital, he hadn't been sectioned or anything, because he just felt so suicidal and depressed [r1]."*

Knowing how to navigate the system: Participants reported difficulties navigating the way into appropriate mental health services. There was confusion over which services were available, which would provide the right support for people experiencing suicidal thoughts and behaviours, and how to initiate contact based on their current patient-status (e.g. service provision differed depending on whether they were a current, discharged, or new patient). At times of suicidal crisis when urgent help was needed knowing how/who to contact for effective support was vital—however, services did not always respond promptly.

> *"We didn't realise that we had a pass [...] back into the mental health system so we went back to the GP [r6]."*

> *"I went to see the doctor and she said [...] I am going to get someone to ring you from the crisis team [...]. Do you know how long it took? They never phoned me five weeks. I suffered like that for five weeks. [s7]."*

Confusion over what services were available and how to access them was not restricted to patients/relatives, other medical professionals such as GPs also struggled when navigating the mental health care system.

For peer review only - http://bmjopen.bmj.com/site/about/guidelines.xhtml

> *"[T]he GP had phoned up the psychiatrist's secretary as well, and had got exactly the same. It was ring five numbers on a piece of paper, she said phone them so I phoned them, and they gave me another number so I phoned them [...] and it went round in a circle, so in the end the fifth person gave me the number of the first person […] and my GP got given the same ring of numbers and went round the same way [s5]."*

Participants also had problem when trying to access services unavailable on the NHS (or only available via long waiting-lists – such as talking therapies). Where participant/relatives were willing and able (e.g. financially) to tap into additional sources of mental health support outside the National Health Service, a wish for guidance and signposting to potentially helpful services by the existing care-team was expressed. In practice this type of guidance was either limited or refused, leaving patients/relatives frustrated.

> *"I said can I short circuit it by paying for it and she sort of said 'I'm not allowed to recommend anyone.' So it was this, I'm trying to do the best for [the patient] and you won't help me [r2]."*

Gate-keepers: The first point of contact during times of increased risk of suicidal behaviour was often the GP, who then acted as a default gate-keeper to accessing secondary mental health services. In this role GPs were viewed either as allies:

> *"I cannot fault my GP, because they […] could not have dealt with it any better [r3]."*

Or as a barrier to be overcome. Gate-keepers' poor attitudes and/or lack of knowledge about how to respond to people with bipolar disorder experiencing suicidal thoughts and behaviours were a common feature in participants' accounts.

> *"I have to say the GPs were very much, 'oh pull yourself together,' you know, sort of attitude [r6]."*

Hospital emergency departments were another critical point of contact and gate-keeping of access to mental health care. Participants were often subject to the negative attitudes of medical staff responsible for their treatment, and for referring those who present with suicidal behaviours to psychiatric teams. Once referred to psychiatric liaison staff there were still issues around gate-keeping of access to appropriate care, demonstrated by failures to recognise bipolar disorder symptomatology and the associated increased risk of suicidal behaviours in this population.

> *"I was as honest as I am being with you, and [psychiatric liaison clinician] just turned to [psychiatric liaison nurse] and he went, 'do you believe a word she's saying?' And this woman just looked at him and went, 'no' and I felt about that big, and I'm sort of thinking I need help… help [s1]."*

For peer review only - http://bmjopen.bmj.com/site/about/guidelines.xhtml

While the quotes above describe first attempts to access mental health care, gate-keepers were also a potential barrier to ongoing successful care for participants/relatives already in contact with secondary mental health services. For example, when the psychiatrist responsible for a patient's care is unavailable at a time of crisis:

> *"[S]o there was another psychiatrist at the hospital, they wouldn't see me because I was under the care of [consultant psychiatrist]. So they wouldn't touch me [s5]."*

Obtaining a correct diagnosis: A common experience among participants was a failure of clinicians to identify or recognise a diagnosis of bipolar disorder. By failing to take account of the individual's bipolar diagnosis clinicians may fail to correctly evaluate increased risk of suicidal behaviour. Lack of a formal diagnosis also impacted on access to care and increased the risk of inappropriate or inadequate medication for those already in contact with services. Inadequate or inappropriate treatment regimens had the potential to exacerbated symptoms and increase suicidal behaviours.

> *"[T]hey put me on antidepressants and I went really, really manic for seven months or something [...]. So I was just left on these antidepressants going higher and higher [s5]."*

Increased risk due to being denied care: The final subtheme that falls under the 'access to care' themes, represents the vulnerability of participants to the emotional and illness-related toll of repeated failures to access care (e.g. when failing to overcome the barriers presented in previous subthemes). Participants were clear that this has a potential to become a cyclic pattern of repeated attempts and failures to access care, with the attendant disappointment and frustration caused precipitating further suicidal thoughts and behaviours; leaving participants/relatives feeling they have to *"scream for help [s2]."*

> *"The crisis team, the home treatment team, nobody would see me, and that is, that is when this [suicidal behaviour] happened, really I was just really desperate [s5].*

There was also a sense of distress at often not being seen as 'ill enough' to deserve mental health care. This was particularly evident when participants looked well or were able to articulate their own concerns about their illness and the risk of engaging in suicidal behaviours (please note, the interview sample had a high level of educational attainment which may be related to the ability to better describe symptoms and distress).

For peer review only - http://bmjopen.bmj.com/site/about/guidelines.xhtml

> *"I find with services they get quite confused you know they look at me and say, 'You are not depressed, you are bathed and clothed.' I am terribly depressed even though I am bathed and clothed [s3]."*

Barriers to accessing care were evident throughout the data corpus, but a minority of participants reported positive experiences whereby access to care for the prevention of suicidal behaviours was facilitated by gatekeepers and/or knowledge of which service to contact in a crisis. Therefore, the barriers discussed here only have the potential to interrupt or block access to care for suicidal behaviour in bipolar disorder.

A model of relationships between subthemes is provided in Figure 1 to aid in interpretation of the 'access to care' theme. 'Help-seeking' is represented as the first step in the model. This connects directly to issues around 'knowing how to navigate the system,' as correct knowledge may facilitate access to care but it may also act as a barrier. This subtheme is then linked to 'gatekeepers' and on to 'obtaining a correct diagnosis.' Another failure at this point leads into the theme of 'increased risk due to being denied care.' The circular path of the arrows, denoting relationships between subthemes, represents the potential for individuals to become trapped in a cycle, this is only one interpretation and barriers may be encountered in any order and access to care maybe achieved at any point.

Problems with communication.

This theme describes the importance of clear communication in the ongoing care of people with bipolar disorder, especially at times of crisis when suicidal behaviour is likely. Paths of communication are between any parties with an interest in the care of a patient, and are here presented in relation to the 'patient', 'relatives', and the 'mental health team'.

Patients: Patients were the main source of information about changes in illness, behaviour, and risk of suicidal behaviour for clinicians and relatives. Some participants acknowledged they/their relative had not always disclosed suicidal intent or increases in suicidal thoughts and behaviour. The consequence of non-disclosure was that carers and services remained awareness of changes in symptoms and behaviours that might indicate increased risk (e.g. non-fatal suicidal behaviour) remained hidden, and opportunities to intervene were lost.

> *"I didn't want a professional to know. It's classic hiding it and not telling anybody, because they have to intervene [s2]."*

Failure to disclosure information that might help manage suicidal behaviour was not always intentional on behalf of the patient. Participants gave examples of memory problems and chaotic

For peer review only - http://bmjopen.bmj.com/site/about/guidelines.xhtml

lifestyles (e.g. being disorganised, substance use problems, or lack of a permanent address are examples of issues that may lead to a chaotic lifestyle) as possible reasons why important changes may not be reported.

> *"I used to get quite cross […], he would say he hadn't seen [his community psychiatric nurse], but when I inquired into it, the more likely thing was that they had gone to his house and he wasn't there [r8]."*

Motivation for failing to communicate suicidal thoughts and behaviours to relatives was driven largely by the wish to protect others from distress and to avoid placing additional burden on relatives.

> *"[S]he didn't want to worry us [r7]."*

> *"I keep a lot to myself and I don't know why I just, I just feel as though if I say things I will upset people [s6]."*

Tangible consequence was that relatives and/or the mental health team were unaware of increasing risk and unable to respond effectively.

> *"I came down and found him in the chair. He took an overdose and he'd promised [the psychiatrist] that he wasn't suicidal. [r1]."*

Relatives: The involvement of relatives in mental health care was generally viewed as positive. Proximity to the patient helped with observation of changes in illness and behaviour. Relatives were also privy to changes in situational factors, such as the presence of stressful or negative life events that might increase the risk of suicidal behaviour, which could then be communicated to the care team.

> *"[W]e were aware that he was drinking. […] I brought all this to the attention of his CPN […] and he was quite shocked by that, and the living conditions, how [the patient] was living [r4]."*

Relatives saw themselves as an untapped resource for services, and wanted to be considered and treated, as full partners in the care of the patient.

> *"[A]s a family we were their biggest resource and they never sort of came to us to help. Either to help us, or to use what we were saying to help him [r4]."*

Not all participants felt supported by relatives or wanted the support of relatives in the management of their illness or suicidal behaviours. And for a couple of participants there was resistance on the part of the family to being involved in care. In these cases a lack of knowledge of bipolar disorder

For peer review only

and the associated risks of suicidal behaviour were cited as obstacles to engaging family members in the care of the patient. Where family involvement was wanted mental health services were viewed as a source of engagement and education for relatives, where working collaboratively would improve support and safety for the patient at home.

> *"I would like somebody to come here and sit them all down and tell them what it involves because I don't think they have a clue [s7]."*

Where familial relationships were poor the involvement of family members was seen as potentially detrimental to the patient and their ongoing care.

> *"[T]the services would be so grateful to have family involved that they push the care to the family where really that is detrimental to me [s3]"*

Participants also gave accounts of conflict between family members and mental health staff. This was particularly evident when relatives felt prevented from contributing to care—which often went hand-in-hand with confusion over patient confidentiality rules.

> *"You just got told on many, many occasions that, you know, its patient confidentiality. […] I turn round and said to them, I said "if my son had cancer, you would have involved me, to care for him quite well at home [r4]."*

This led to failures in communicating information that had potential value for assessing risk. Relatives expressed frustration at being unable to help the patient despite being in possession of knowledge that may have influenced care.

> *"[W]hen he died, you know, the psychiatrist had actually said that he didn't think it was planned suicide, […] even though he had been telling me that he wanted, you know, he didn't want to live. He wanted to die for probably months [r6]."*

Mental health teams: Participants gave multiple accounts of communication failures between patients and the care team. Poor explanations of diagnosis and prognosis, risks and triggers, medication regimens and possible side effects of treatments all contributed to patients feeling ignored or dismissed by clinical teams. The consequence of this was poorer management, weaker therapeutic relationships, and ultimately the potential to miss indicators of increased risk.

> *"[T]hey never said to me, you know your mom has mental illness you might need to you know go and get yourself checked out at the doctors, with your suicidal thoughts, suicidal tendencies... [s4]."*

For peer review only - http://bmjopen.bmj.com/site/about/guidelines.xhtml

Following episodes of suicidal behaviour, some participants reported being left without any follow-up or acknowledgment from clinical teams. Subsequently there was no adjustment of treatment, additional interventions, or changes in monitoring in response to the suicidal behaviour.

> *"[O]ne of the home treatment team came into the appointment and sat in the room with me, and neither she nor the psychiatrist mentioned the fact that I had self-harmed and run away [s5]."*

There was evidence that communication between services and patients has improved over time. With accounts of new teams and initiatives created to better ensure the safety of patients, and participants were hopeful that services would continue to improve.

> *"[S]he came out on the Friday and brought the crisis team out […] they said if I needed them over the weekend that they could you know, I could ring them and they would come and see me. So you have a bigger support work now than you did a few years ago [s4]."*

The theme 'problems with communication' encapsulates many of the vulnerabilities in routes of communication between patients, relatives, and mental health services, in the provision of care for people with bipolar disorder and suicidal behaviour. It is interesting to note that the converse of this was also true. Participants' judgements of the quality of care they/their relative received were more positive in cases where the care team were seen as collaborative and inclusive. Where communication between parties was open and opinions on quality of care received were positive regardless of outcome (which for the relatives group was bereavement by suicide).

> *"[T]there was one guy, he was one of the few [health care professionals] that both [the patient] and I trusted because he was quite happy to have us both in at the same time [r2]."*

Sympathy and trust were key features of positive evaluations of care received. Clinicians who were willing to provide and receive information from patients and relatives were valued. Continuity of care to establish long-term therapeutic relationships were also vital for building trust, and allowed clinicians greater understanding of individual patients' illness, behaviour, and situation; aiding monitoring of bipolar disorder symptoms and identification of times of increased risk of suicidal behaviour. Where these factors were lacking, participants evaluated care as poor.

> *"I gave up [on] the psychiatrist because they were a bloody waste of time, they didn't know me. […] They just read the notes and there was no connection at all [s7]."*

A model of subthemes and vulnerabilities in communication is provided in Figure 2 to aid interpretation of the 'problem with communication' theme. 'Patients,' 'relatives,' and 'mental health

For peer review only - http://bmjopen.bmj.com/site/about/guidelines.xhtml

teams' are connected to each other and communication can flow in any direction. When communication is open and positive care is viewed as more collaborative and satisfaction with the care team is higher. Examples of problems in communication are given outside the pathways of communication with arrows denoting the possibility that any of these issues could interrupt communication at any time, and impact on the care of the patient.

DISCUSSION

This exploratory study used a qualitative approach to investigate experiences of mental health care in relation to suicidal behaviour in bipolar disorder. Two main themes captured the focus of participants' accounts; access to care, and problems in communication, both of which may contribute to failures to identify and respond to increased risk of suicide in bipolar disorder.

A number of subthemes identified were consistent with experiences common among psychiatric groups in general [19,20]. And while this does not diminish the importance of these concerns, their impact on the individual, or the possibility these difficulties may be over-represented among people with suicidal behaviour in bipolar disorder, there focus here is on the identification of novel factors that may be specific to care for suicidal behaviours in bipolar disorder.

Active help-seeking was common, with the majority of participants recognising the need for increased support when experiencing increased suicidal thoughts and behaviours. Barriers were predominantly external, such as gate-keeping and poor staff attitudes to suicidal behaviours [39,40].There was no evidence of issues relating to stigma or other individual-centred barriers as documented in more general studies of people with bipolar disorder [19,20]. However, it is possible that internalised stigma may have played a role in the decision not to disclose suicidal thoughts to relatives or care teams, which falls under the theme of 'problems with communication' [41].

One aspect of gate-keeping that may be particularly relevant people with bipolar disorder was the assumption of low illness severity (and therefore risk of suicidal behaviours) based on superficial aspects of participants' appearance and the ability to effectively communicate concerns and distress. Bipolar disorder is characterised by periods of illness and relative stability when patients may be better able to effectively recognise and communicate distress and risk [3]. There is also some evidence that, compared to other psychiatric groups, people with bipolar disorder have an educational attainment level similar to the general population [42,43]. Although it did not appear in the themes presented here it may be important to note that there was a high level of educational

For peer review only - http://bmjopen.bmj.com/site/about/guidelines.xhtml

attainment across the interview sample – with many undertaking higher degrees—perhaps suggesting this sample may have had more effective communication skills, especially when describing risk of suicidal behaviours.

Ensuring accurate diagnosis was critical in the management of suicidal behaviours in bipolar disorder. Compared to other psychiatric diagnoses, an accurate diagnosis of bipolar disorder is commonly delayed due to misdiagnosis of initial symptoms – especially when symptoms of depression are present, [44]. The accounts of participants, where inappropriate treatments sometimes exacerbated symptoms and increased risk of suicidal behaviours, emphasise the importance of increasing clinician knowledge—especially for those who may be a first point of contact such as GPs and psychiatric liaison staff—in recognition of possible bipolar disorder and the risks associated with this illness.

Mental health care for suicidal behaviour was enhanced by involving patients and relatives (where appropriate) in care decisions and resulted in positive evaluations of care even after the suicide of a loved-one. Work by Owen et al (2015) on the impact of social factors in suicidal thoughts, showed that 'not being understood or acknowledged,' and 'feeling burdensome,' triggered or exacerbated suicidal thoughts and acts [24]. Participants' accounts of problems in communication in the current work echo these results, especially in relation to non-disclosure of suicidal intent due to not wanting to burden family members. However, participants often wanted the support of family, and the involvement of family in care decisions was highly valued. Furthermore, therapies involving family members (e.g. family-focused therapy, psychoeducation) have been shown to help reduce relapse, symptom severity and caregiver burden may also be effective in addressing some of the communication blocks identified in this study. This may be especially true of therapies that seek to educate patients and families by increasing knowledge bipolar disorder and the associated increased risk [45,46]. Recent work examining the experiences of mental health care by people with bipolar disorder showed that meaningful information about bipolar disorder and it's treatment (as well as psychosocial interventions and access to peer support) resulted in more positive attitudes towards care and better engagement with services [29].

Previous qualitative work showed that people with serious mental illness struggle to be seen as competent and equal partners in care decisions [47] – and this was evident in accounts given here where the focus was specifically on help for suicidal behaviours. Our work also supports the idea that relatives and carers often feel excluded from mental health care [28]. As in general mental health care for people with bipolar disorder, care received for suicidal behaviour was viewed more

For peer review only - http://bmjopen.bmj.com/site/about/guidelines.xhtml

positively across interviews when care was collaborative and clinicians were sympathetic, open, and interested in involving patients and relatives [48].

Methodological limitations

Total number of participants was small but sufficient to provide a novel insight into the experiences of a small and hard to reach population--especially with regard to relatives—via rich in-depth data. Relatives may have inherently different perspectives on mental health care, their role, and concerns, following bereavement by suicide, than patients. As relatives were a more homogeneous group the commonalities of these accounts may have had greater influence on resulting themes. Although this does not reduce the validity of the themes presented, efforts were made throughout the results to give supporting quotes from both participant groups. Themes and conceptual models presented in this work do not attempt to give an exhaustive account of all experiences of accessing mental health care by people with suicidal behaviours in bipolar disorder. However, identification factors considered important by participants with diverse perspectives and experiences have been described, and common difficulties faced by this high-risk group when trying to access care identified.

Clinical implications

Many people who experience suicidal behaviour in bipolar disorder are in close proximity to care services at the time of the act. While the influence of rapidly changing mood-states and impulsivity make prediction and prevention of suicidal behaviours in this group particularly difficult, people with experience of suicidal behaviours in bipolar disorder may have a role in helping to improve the response of mental health services [49–51]. Rapid access to care is crucial, and patients and relatives need good knowledge of what services are available to them, with clearly signposted ways to access services. Relatives are often in a position to observe behavioural changes that may indicate increased or fluctuating risk, and where possible, should be kept informed of treatment/management plans. Closer involvement of family and friends may allow important information to be communicated more quickly, help build trust and supportive therapeutic relationships, and create more positive judgements of overall quality of care provided by mental health services [52].

There may also be a role for improvement in health professionals' knowledge of bipolar disorder and the increased risk of suicide conferred by this illness. Participants' accounts consistently showed some level of underestimation of illness severity and suicide risk by clinicians, even for individuals under mental health care. A combination of greater knowledge of bipolar disorder in general and

For peer review only - http://bmjopen.bmj.com/site/about/guidelines.xhtml

increased communication skills in the identification of suicidal thoughts and behaviours may be beneficial.

Additional interview work with clinicians may help in understanding problems in communication about suicidal behaviour in bipolar disorder, and help explore the perception of lower risk in people who are better able to communicate their distress and concerns, as found in the current work. Further work with a wider range of participants, particularly those from less educated backgrounds where additional barriers may include health literacy, is needed to explore the generalisability of the themes presented, as well as how they could be translated into improved care practices.

Author contributions: NK and CC designed the study with input from all authors. CC collected all data. CC and SP analysed and interpreted the data, with input from all authors. CC drafted the manuscript. NK, SP, RM, SJ provided further input on subsequent drafts of the manuscript.

Funding: This article presents independent research commissioned by the National Institute for Health Research (NIHR) under its Programme Grants for Applied Research funding scheme (RP-PG-0407-10389). The views expressed in this work are those of the authors and not those of the NHS, NIHR, or Department of Health.

Competing interests: None

Patient consent for publication: Not required

Data statement: Due to confidentiality full interview data cannot be made available.

For peer review only

REFERENCES

1 Merikangas KR, Jin R, He J, *et al.* Prevalence and correlates of bipolar spectrum disorder in the world mental health survey initiative. *Arch Gen Psychiatry* 2011;**68**:241–51. doi:10.1001/archgenpsychiatry.2011.12.Prevalence

2 Michalak EE, Murray G, Collaborative RESearch Team to Study Psychosocial Issues in Bipolar Disorder (CREST.BD). Development of the QoL.BD: a disorder-specific scale to assess quality of life in bipolar disorder. *Bipolar Disord* 2010;**12**:727–40. doi:10.1111/j.1399-5618.2010.00865.x

3 Goodwin FK, Jamison KR. *Manic-Depressive Illness:Bipolar Disorders and Recurrent Depression: Bipolar Disorders and Recurrent Depression*. Oxford University Press, USA 2007. http://books.google.co.uk/books?id=X7XLhltqsv8C

4 Schaffer A, Isometsä ET, Tondo L, *et al.* International Society for Bipolar Disorders Task Force on Suicide: Meta-analyses and meta-regression of correlates of suicide attempts and suicide deaths in bipolar disorder. *Bipolar Disord* 2015;**17**:1–16. doi:10.1111/bdi.12271

5 Tondo L, Pompili M, Forte A, *et al.* Suicide attempts in bipolar disorders: Comprehensive review of 101 reports. *Acta Psychiatr Scand* 2016;**133**:174–86. doi:10.1111/acps.12517

6 Pompili M, Gonda X, Serafini G, *et al.* Epidemiology of suicide in bipolar disorders: a systematic review of the literature. *Bipolar Disord* 2013;**15**:457–90. doi:10.1111/bdi.12087

7 Nordentoft M, Mortensen PBB, Pedersen CB. Absolute risk of suicide after first hospital contact in mental disorder. *Arch Gen Psychiatry* 2011;**68**:1058–64. doi:10.1001/archgenpsychiatry.2011.113

8 Watkins HB, Meyer TD. Is there an empirical link between impulsivity and suicidality in bipolar disorders? A review of the current literature and the potential psychological implications of the relationship. *Bipolar Disord* 2013;**15**:542–58. doi:10.1111/bdi.12090

9 Gonda X, Pompili M, Serafini G, *et al.* Suicidal behavior in bipolar disorder: epidemiology, characteristics and major risk factors. *J Affect Disord* 2012;**143**:16–26. doi:10.1016/j.jad.2012.04.041

10 Clements C, Morriss R, Jones S, *et al.* Suicide in bipolar disorder in a national English sample, 1996-2009: Frequency, trends and characteristics. *Psychol Med* 2013;**43**.

For peer review only - http://bmjopen.bmj.com/site/about/guidelines.xhtml

doi:10.1017/S0033291713000329

11 Clements C, Jones S, Morriss R, *et al.* Self-harm in bipolar disorder: Findings from a prospective clinical database. *J Affect Disord* 2015;**173**:113–9. doi:10.1016/j.jad.2014.10.012

12 Smith KA, Cipriani A. Lithium and suicide in mood disorders: Updated meta-review of the scientific literature. *Bipolar Disord* 2017;**19**:575–86. doi:10.1111/bdi.12543

13 Baldessarini RJ, Tondo L, Vázquez GH. Pharmacological treatment of adult bipolar disorder. *Mol Psychiatry* 2018;:1–20. doi:10.1038/s41380-018-0044-2

14 Tarrier N, Taylor K, Gooding P. Cognitive-behavioral interventions to reduce suicide behavior: a systematic review and meta-analysis. *Behav Modif* 2008;**32**:77–108. doi:10.1177/0145445507304728

15 Rihmer Z, Gonda X, Tondo L. Lithium and suicidal behavior in patients with bipolar disorder. *J Clin Psychiatry* 2008;**69**:1831–2.http://www.ncbi.nlm.nih.gov/pubmed/19200432 (accessed 12 May 2014).

16 Cipriani A, Hawton K, Stockton S, *et al.* Lithium in the prevention of suicide in mood disorders: updated systematic review and meta-analysis. *BMJ* 2013;**3646**:1–13. doi:10.1136/bmj.f3646

17 Anderson KK, Fuhrer R, Malla AK. “There are too many steps before you get to where you need to be”: Help-seeking by patients with first-episode psychosis. *J Ment Heal* 2012;**22**:384–95. doi:10.3109/09638237.2012.705922

18 Smith TE, Easter A, Pollock M, *et al.* Disengagement From Care: Perspectives of Individuals With Serious Mental Illness and of Service Providers. *Psychiatr Serv* 2013;**64**:770–5. doi:10.1176/appi.ps.201200394

19 Clement S, Schauman O, Graham T, *et al.* What is the impact of mental health-related stigma on help-seeking? A systematic review of quantitative and qualitative studies. *Psychol Med* 2015;**45**:11–27. doi:10.1017/S0033291714000129

20 Dockery L, Jeffery D, Schauman O, *et al.* Stigma- and non-stigma-related treatment barriers to mental healthcare reported by service users and caregivers. *Psychiatry Res* 2015;**228**:612–9. doi:10.1016/j.psychres.2015.05.044

21 Owens C, Lambert H, Donovan J, *et al.* A qualitative study of help seeking and primary care

For peer review only

consultation prior to suicide. *Br J Gen Pract* 2005;**55**:503–9.

22 Taylor TL, Hawton K, Fortune S, *et al.* Attitudes towards clinical services among people who self-harm: Systematic review. *Br J Psychiatry* 2009;**194**:104–10. doi:10.1192/bjp.bp.107.046425

23 Jordan J, McKenna H, Keeney S, *et al.* Providing meaningful care: learning from the experiences of suicidal young men. *Qual Heal Res* 2012;**22**:1207–19. doi:10.1177/1049732312450367

24 Owen R, Gooding P, Dempsey R, *et al.* A qualitative investigation into the relationships between social factors and suicidal thoughts and acts experienced by people with a bipolar disorder diagnosis. *J Affect Disord* 2015;**176**:133–40. doi:10.1016/j.jad.2015.02.002

25 Bilderbeck AC, Saunders KEA, Price J, *et al.* Psychiatric assessment of mood instability: qualitative study of patient experience. *Br J Psychiatry* 2014;**204**:234–9. doi:10.1192/bjp.bp.113.128348

26 Highet N, McNair B, Thompson M, *et al.* Experience with treatment services for people with bipolar disorder. *Med J Aust* 2004;**181**:47–51.https://www.mja.com.au/journal/2004/181/7/experience-treatment-services-people-bipolar-disorder

27 Chatzidamianos G, Lobban F, Jones S. A qualitative analysis of relatives', health professionals' and service users' views on the involvement in care of relatives in Bipolar Disorder. *BMC Psychiatry* 2015;**15**:228. doi:10.1186/s12888-015-0611-x

28 Cree L, Brooks HL, Berzins K, *et al.* Carers ' experiences of involvement in care planning : a qualitative exploration of the facilitators and barriers to engagement with mental health services. *BMC Psychiatry* 2015;:1–11. doi:10.1186/s12888-015-0590-y

29 Vallarino M, Rapisarda F, Scott J, *et al.* Experiences of Mental Healthcare Reported by Individuals Diagnosed with Bipolar Disorder: An Italian Qualitative Study. *Community Ment Health J* 2019;**55**:129–36. doi:10.1007/s10597-018-0311-3

30 Hawton K, Harriss L, Hall S, *et al.* Deliberate self-harm in Oxford, 1990-2000: a time of change in patient characteristics. *Psychol Med* 2003;**33**:987–95.http://www.ncbi.nlm.nih.gov/pubmed/12946083 (accessed 26 May 2014).

For peer review only

31 Isometsä ET. Psychological autopsy studies--a review. *Eur Psychiatry* 2001;**16**:379–85.http://www.ncbi.nlm.nih.gov/pubmed/11728849

32 Sher L. Psychological autopsy studies: Past, present, and future. *Aust New Zeal J Psychiatry* 2013;**47**:884–884. doi:10.1177/0004867413479071

33 Owens D, Kelley R, Munyombwe T, *et al.* Switching methods of self-harm at repeat episodes: Findings from a multicentre cohort study. *J Affect Disord* 2015;**180**:44–51. doi:10.1016/j.jad.2015.03.051

34 Trier-Bieniek a. Framing the telephone interview as a participant-centred tool for qualitative research: a methodological discussion. *Qual Res* 2012;**12**:630–44. doi:10.1177/1468794112439005

35 Sturges J, Hanrahan K. Comparing telephone and face-to-face qualitative interviewing: a research note. *Qual Res* 2004;**4**:107–18.

36 Shuy R. In-person versus telephone interviewing. In: Gubrium J, Holstein J, eds. *Handbook of Interview Research: context and method*. Thousand Oaks, CA: : Sage 2001. 536–54. doi:10.4135/9781412973588

37 Creswell J. *Qualitative inquiry and research design: choosing between five approaches*. Third Edit. Thousand Oaks, CA: : Sage 2012.

38 Braun V, Clarke V. Using thematic analysis in psychology. *Qual Res Psychol* 2006;**3**:77–101. doi:10.1191/1478088706qp063oa

39 Anthony JS, Baik RNS, Bowers RNBJ, *et al.* Conditions That Influence a Primary Care Clinician ' s Decision to Refer Patients for Depression Care. *Rehabil Nurs* 2010;**35**:113–22.

40 Saunders KEA, Hawton K, Fortune S, *et al.* Attitudes and knowledge of clinical staff regarding people who self-harm: A systematic review. *J Affect Disord* 2012;**139**:205–16. doi:10.1016/j.jad.2011.08.024

41 Hawke LD, Parikh S V, Michalak EE. Stigma and bipolar disorder: a review of the literature. *J Affect Disord* 2013;**150**:181–91. doi:10.1016/j.jad.2013.05.030

42 Schoeyen HK, Melle I, Sundet K, *et al.* Occupational outcome in bipolar disorder is not predicted by premorbid functioning and intelligence. *Bipolar Disord* 2013;**15**:294–305.

For peer review only - http://bmjopen.bmj.com/site/about/guidelines.xhtml

For peer review only

doi:10.1111/bdi.12056

43 Dickinson D, Harvey PD. Systemic hypotheses for generalized cognitive deficits in schizophrenia: a new take on an old problem. *Schizophr Bull* 2009;**35**:403–14. doi:10.1093/schbul/sbn097

44 Altamura AC, Buoli M, Caldiroli A, *et al.* Misdiagnosis, duration of untreated illness (DUI) and outcome in bipolar patients with psychotic symptoms: A naturalistic study. *J Affect Disord* 2015;**182**:70–5. doi:10.1016/j.jad.2015.04.024

45 Miklowitz DJ, Chung B. Family-Focused Therapy for Bipolar Disorder: Reflections on 30 Years of Research. *Fam Process* 2016;**55**:483–99. doi:10.1111/famp.12237

46 Baruch E, Pistrang N, Barker C. Psychological interventions for caregivers of people with bipolar disorder: A systematic review and meta-analysis. *J Affect Disord* 2018;**236**:187–98. doi:10.1016/j.jad.2018.04.077

47 Dahlqvist Jönsson P, Schön U-K, Rosenberg D, *et al.* Service users' experiences of participation in decision making in mental health services. *J Psychiatr Ment Health Nurs* 2015;**22**:688–97. doi:10.1111/jpm.12246

48 Norcross JC, Wampold BE. Evidence-based therapy relationships: research conclusions and clinical practices. *Psychotherapy (Chic)* 2011;**48**:98–102. doi:10.1037/a0022161

49 Cassidy F. Risk factors of attempted suicide in bipolar disorder. *Suicide Life Threat Behav* 2011;**41**:6–11. doi:10.1111/j.1943-278X.2010.00007.x

50 D'Ambrosio V, Salvi V, Bogetto F, *et al.* Serum lipids, metabolic syndrome and lifetime suicide attempts in patients with bipolar disorder. *Prog Neuropsychopharmacol Biol Psychiatry* 2012;**37**:136–40. doi:10.1016/j.pnpbp.2011.12.009

51 Tondo L, Baldessarini R. Suicidal risk in bipolar disorder. *Clin Neuropsychiatry* 2005;**2**:55–65.

52 Dirik A, Sandhu S, Giacco D, *et al.* Why involve families in acute mental healthcare? A collaborative conceptual review. *BMJ Open* 2017;**7**:1–10. doi:10.1136/bmjopen-2017-017680

For peer review only - http://bmjopen.bmj.com/site/about/guidelines.xhtml

Table 1. Gender and age of interview participants

**Variable**	**Participants**<br>**n=22(%)**
**Sex**	
Male	4 (18.2)
Female	18 (81.8)
**Age group**	
18-35	4 (18.2)
36-50	10 (45.5)
51-65	4 (18.2)
66>	4 (18.2)

For peer review only

For peer review only - http://bmjopen.bmj.com/site/about/guidelines.xhtml

Figure 1: Model of subthemes within the main theme 'access to care'.

Figure 2: Model of subthemes within the main theme 'problems with communication'.

For peer review only

For peer review only - http://bmjopen.bmj.com/site/about/guidelines.xhtml

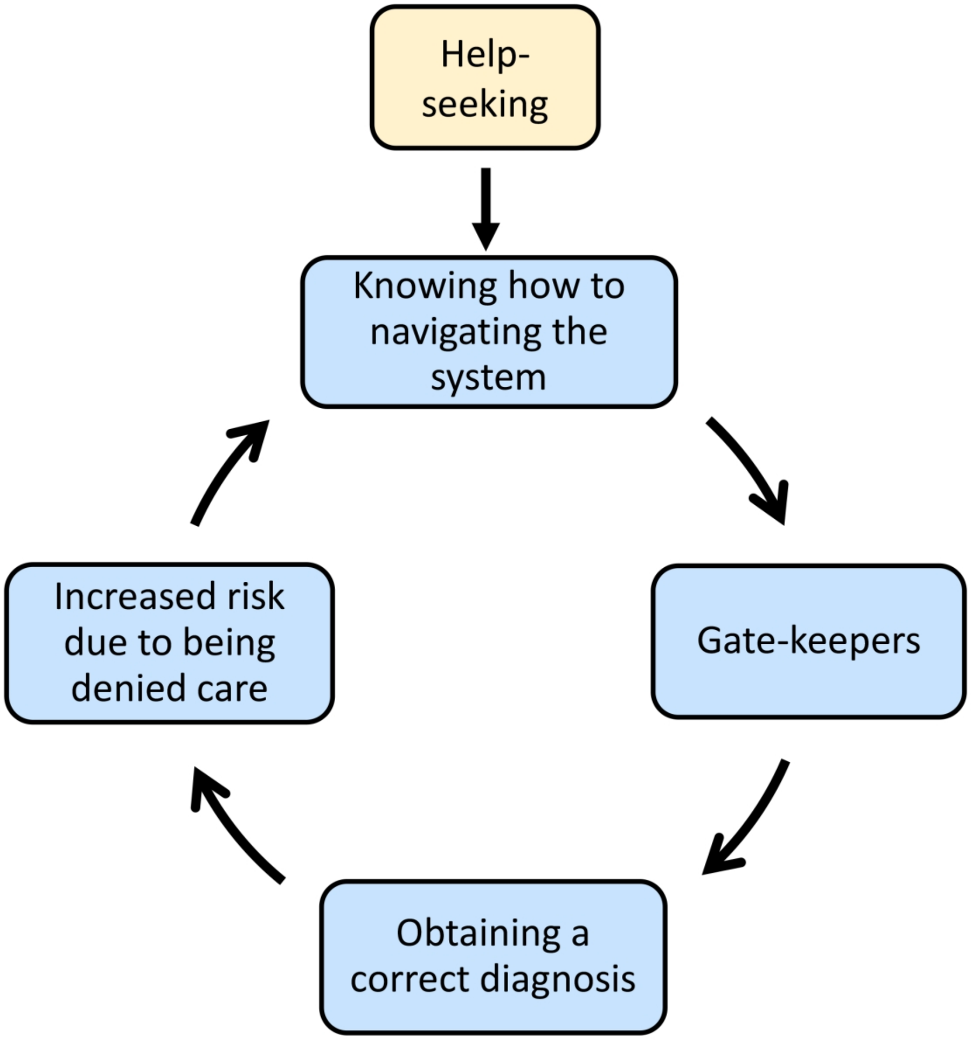

Figure 1. Model of subthemes within the main theme 'access to care'.

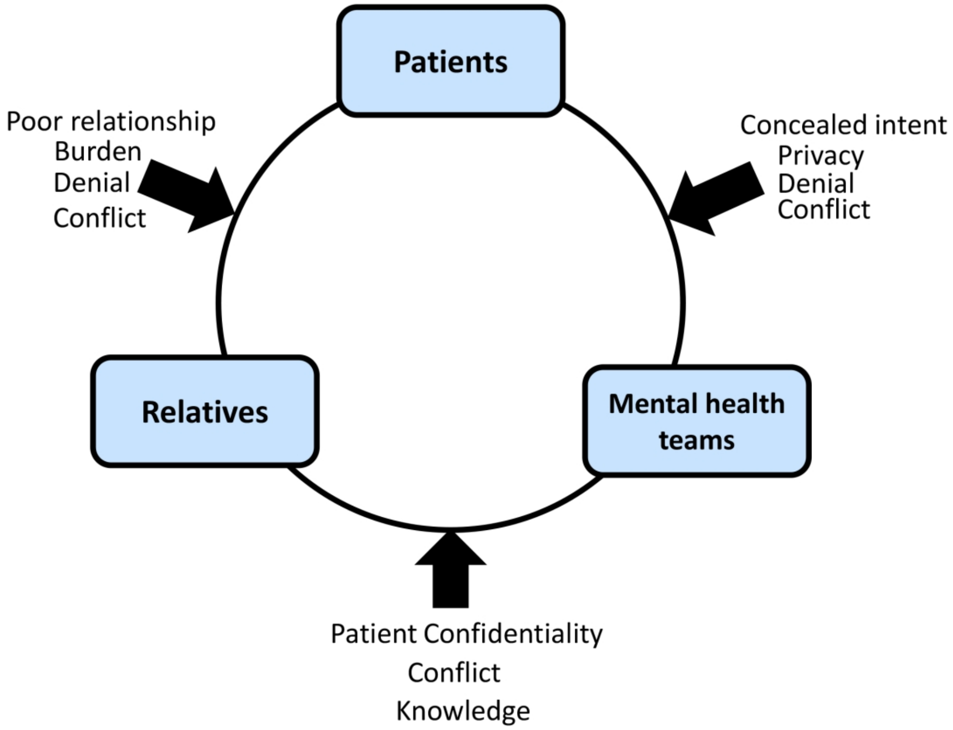

Figure 2. Model of subthemes within the main theme 'problems with communication'.

Supplementary Table S1: Account of the method used to identify themes, adapted from Braun and Clarke (2006).

Familiarisation with data corpus.<br>Analyses started when two relatives and two self-harm participants' interviews had been completed and transcribed in full. Close reading of the transcripts by CC and SP enabled familiarisation with the data. Interviews and analysis were conducted in parallel and additional transcripts were incorporated into the analysis as they became available. Back-coding of older transcripts was carried out when new codes were identified.
Generation of initial codes.<br>Coding was conducted by CC and SP in order to confirm concordance of coding and ideas during analysis. Interesting features within the participants' accounts were highlighted and allocated preliminary codes. These coded extracts were compiled and organised within a working text document. Examples of early codes included: 'family fighting for additional care,' 'changes in personality not recorded by services,' 'hiding suicidal behaviour,' 'not wanting to be a burden to family.'
Identification of themes.<br>Through an iterative process of discussion and review CC and SP consolidated codes into potential themes by grouping coded extracts that represented the same or similar issues within the working document. While the accounts were initially analyses as a single group (of people with experience of suicidal behaviours in bipolar disorder) at this point attention was given to whether themes were consistent or discriminant between the accounts of the two participant populations (i.e. people with bipolar and suicidal behaviour disorder and people bereaved by the suicide of someone with bipolar disorder). Analysis continued until thematic saturation was achieved, and no new ideas relating to the research topic emerged.
Review and definition of themes.<br>Themes identified were summarised and presented for discussion with the extended multidisciplinary research team at team meetings. Themes were presented along with supporting quotes from across the participants group. Themes were accepted as valid once there was unanimous agreement across the research team that the theme was present and accurately captured the experiences of participants.
Reporting the analysis.<br>Appropriate supporting quotes were selected from participants' account for inclusion in the report to illustrate each theme and associated subthemes. Models (Figures 1 and 2) were constructed to help describe the relationship between subthemes and aid understanding. Models were confirmed as appropriate via review and discussion with the research team. Results were then contextualized with reference to existing literature on care for suicidal behaviour in bipolar disorder.

For peer review only

Supplementary Table S2: Full quotes relating to the theme 'the merry-go-round of access to mental health care.'

Help-seeking
"He'd put himself in hospital, he hadn't been sectioned or anything, because he just felt so suicidal and depressed [r1]."
"I want to feel better, I don't want to be feeling low and you know, not happy [s6]."
Navigating the system
"We didn't realise that we had a pass, if you like, back into the mental health system so we went back to the GP [r6]."
"I went to see the doctor and she said to me, right […] I am going to get someone to ring you from the crisis team, and you need somebody to talk to. Do you know how long it took? They never phoned me five weeks. I suffered like that for five weeks. It took five weeks, and in the end I have to phone up, and then they had no recognition of who I was [s7]."
"[T]he GP had phoned up the psychiatrist's secretary as well, and had got exactly the same. It was ring five numbers on a piece of paper, she said phone them so I phoned them, and they gave me another number so I phoned them, and they gave me another number so I phoned them, and they gave me another number, and it went round in a circle, so in the end the fifth person gave me the number of the first person to phone so I had been round in a ring […]and my GP got given the same ring of numbers and went round the same way [s5]"
"I said can I short circuit it by paying for it and she sort of said 'I'm not allowed to recommend anyone.' So it was this, I'm trying to do the best for [the patient] and you won't help me [r2]."
Gate-keepers
"I cannot fault my GP, because they […] could not have dealt with it any better. He was wonderful, and got us into the psychiatric hospital as soon, the same day, which was just unbelievable [r3]."
"I have to say the GPs were very much, 'oh pull yourself together,' you know, sort of attitude [r6]."

For peer review only

"I spent 3 days in and out of A&E begging them to help me [s2]."
"I was as honest as I am being with you, and [psychiatric liaison clinician] just turned to [psychiatric liaison nurse] and he went, 'do you believe a word she's saying?' And this woman just looked at him and went, 'no' and I felt about that big, and I'm sort of thinking I need help… help [s1]"
"Nobody else would see me, so there was another psychiatrist at the hospital, they wouldn't see me because I was under the care of [consultant psychiatrist]. So they wouldn't touch me, so... it just shows how you can fall out of the system really [s5]."
Formal diagnosis
"[T]hey put me on antidepressants and I went really, really manic for seven months or something, and I ended up just leaving. Oh, at the hospital I got arrested because I threw a bin through a window. And they had me arrested and then refused to treat me, which is where I was getting the treatment. So I was just left on these antidepressants [without a mood stabiliser] *going higher and higher [s5]."*
Screaming for help
"The crisis team, the home treatment team, nobody would see me, and that is, that is when this [suicidal behaviour] happened, really I was just really desperate [s5].
"And also I present always with a mask and I think that's why I was diagnosed so late. You know because I am always, and I find with services they get quite confused you know they look at me and say , 'You are not depressed, you are bathed and clothed.' I am terribly depressed even though I am bathed and clothed [s3]."
"It used to make him angry, cross, you know, and he couldn't get people to sort of listen, and to help him [r8]."

For peer review only

Supplementary Table S3: Full quotes supporting the theme Routes and Road blocks in communication.

Evaluations of Care
"Most of them they excluded me […] there was one guy, he was one of the few people [health care professionals] *that both [the patient] and I trusted because he was quite happy to have us both in at the same time [r2]."*
"I gave up [on] *the psychiatrist because they were a bloody waste of time, they didn't know me. They didn't know who I was. They just read the notes and there was no connection at all [s7]."*
"I mean I can't remember how many different people, social workers have dealt with him. It would be sort of a few months and then they were having a baby, fair enough the woman would be having a baby, but you know it doesn't help the person you are working with [r8]."
Patients' communication
"She was putting up this front that she was absolutely fine, and that was the really big message that was coming across [r5]."
"I didn't want a professional to know. It's classic hiding it and not telling anybody, because they have to intervene [s2]."
"I had a suicide plan in place, or whatever they call it, and wouldn't reveal the details of that to him [the psychiatrist] *[s5]"*
"I used to get quite cross because, you know, he would say he hadn't seen this person [community psychiatric nurse], but when I inquired into it, the more likely thing was that they had gone to his house and he wasn't there [r8]."
"She had attempted it maybe about four times, but she didn't want us to know, she didn't want to worry us [r7]."
"I have got like a really good family, but I find it difficult to let people know a lot of stuff,

For peer review only

they do know, but I keep a lot to myself and I don't know why I just, I just feel as though if I say things I will upset people [s6].”
“I came down and found him in the chair. He took an overdose and he’d promised [the psychiatrist] that he wasn’t suicidal. He said that he was alright now things were getting done [r1].”
Relatives’ Communication
“[W]e were aware that he was drinking. I brought all this, about a month before [the patient] died, I brought all this to the attention of his CPN […] and he was quite shocked by that, and the living conditions, how [the patient] was living [r4].”
“I had been delusional for weeks and weeks and I was really poorly, and my daughter was ringing the mental health service saying you need to come and check my mum she is not, my mum is not well she is acting in a weird way, she is walking round naked, she is not good, you need to come you know, and they didn't come [s4].”
“[A]s a family we were their biggest resource and they never sort of came to us to help. Either to help us, or to use what we were saying to help him [r4].”
“I feel so sorry for anybody who had got depression or anything like that got no chance unless you have got somebody to help you [s7].”
“I would like somebody to come here and sit them all down and tell them what it involves because I don't think they have a clue [s7].”
“I have written an advanced statement, because you know the services would be so grateful to have family involved that they push the care to the family, where really that is detrimental to me [s3]”
“You just got told on many, many occasions that, you know, its patient confidentiality. But, when you’re dealing with somebody who’s suffering from mental health, and if I turn round and said to them, I said “if my son had cancer, you would have involved me, to care for him quite well at home [r4].”

For peer review only

"[W]hen he died, you know, the psychiatrist had actually said that he didn't think it was planned suicide, because he didn't think [the patient] was in that frame of mind to make that decision at that time, even though, even though he had been telling me that he wanted, you know, he didn't want to live. He wanted to die for probably months [r6]."
"[W]hen he died, you know, the psychiatrist had actually said that he didn't think it was planned suicide, because he didn't think [the patient] was in that frame of mind to make that decision at that time, even though, even though he had been telling me that he wanted, you know, he didn't want to live. He wanted to die for probably months [r6]."
Services' communication.
"[T]hey never said to me, you know your mom has mental illness you might need to you know go and get yourself checked out at the doctors, with your suicidal thoughts, suicidal tendencies... they didn't do it, they didn't do it [s4]."
"[I]n the appointment that I had, after I had self-harmed and run away, […] one of the home treatment team came into the appointment and sat in the room with me, and neither she nor the psychiatrist mentioned the fact that I had self-harmed and run away [s5]."
"[W]hen I spoke to [community psychiatric nurse] on the Thursday after me thinking about suicide on the Wednesday, she spoke to the psychiatrist and then she spoke to the crisis team, and she came out on the Friday and brought the crisis team out on the Friday and said, and they said if I needed them over the weekend that they could you know, I could ring them and they would come and see me. So you have a bigger support work now than you did a few years ago, because there wasn't no crisis team a few years ago [s4]."

For peer review only

Reporting checklist for qualitative study.

Based on the SRQR guidelines.

Instructions to authors

Complete this checklist by entering the page numbers from your manuscript where readers will find each of the items listed below.

Your article may not currently address all the items on the checklist. Please modify your text to include the missing information. If you are certain that an item does not apply, please write "n/a" and provide a short explanation.

Upload your completed checklist as an extra file when you submit to a journal.

In your methods section, say that you used the SRQR reporting guidelines, and cite them as:

O'Brien BC, Harris IB, Beckman TJ, Reed DA, Cook DA. Standards for reporting qualitative research: a synthesis of recommendations. Acad Med. 2014;89(9):1245-1251.

	Reporting Item	Page Number
#1	Concise description of the nature and topic of the study identifying the study as qualitative or indicating the approach (e.g. ethnography, grounded theory) or data collection methods (e.g. interview, focus group) is recommended	2
#2	Summary of the key elements of the study using the abstract format of the intended publication; typically	2

For peer review only

		includes background, purpose, methods, results and conclusions	
Problem formulation	#3	Description and signifcance of the problem / phenomenon studied: review of relevant theory and empirical work; problem statement	4/5
Purpose or research question	#4	Purpose of the study and specific objectives or questions	5
Qualitative approach and research paradigm	#5	Qualitative approach (e.g. ethnography, grounded theory, case study, phenomenolgy, narrative research) and guiding theory if appropriate; identifying the research paradigm (e.g. postpositivist, constructivist / interpretivist) is also recommended; rationale. The rationale should briefly discuss the justification for choosing that theory, approach, method or technique rather than other options available; the assumptions and limitations implicit in those choices and how those choices influence study conclusions and transferability. As appropriate the rationale for several items might be discussed together.	5-7
Researcher characteristics and reflexivity	#6	Researchers' characteristics that may influence the research, including personal attributes, qualifications / experience, relationship with participants, assumptions and / or presuppositions; potential or actual interaction between researchers' characteristics and the research questions, approach, methods, results and / or transferability	6

For peer review only

Context	#7	Setting / site and salient contextual factors; rationale	6-7
Sampling strategy	#8	How and why research participants, documents, or events were selected; criteria for deciding when no further sampling was necessary (e.g. sampling saturation); rationale	6-7
Ethical issues pertaining to human subjects	#9	6-7	4
Data collection methods	#10	Types of data collected; details of data collection procedures including (as appropriate) start and stop dates of data collection and analysis, iterative process, triangulation of sources / methods, and modification of procedures in response to evolving study findings; rationale	6-7
Data collection instruments and technologies	#11	Description of instruments (e.g. interview guides, questionnaires) and devices (e.g. audio recorders) used for data collection; if / how the instruments(s) changed over the course of the study	6-7
Units of study	#12	Number and relevant characteristics of participants, documents, or events included in the study; level of participation (could be reported in results)	6-7
Data processing	#13	Methods for processing data prior to and during analysis, including transcription, data entry, data management and security, verification of data integrity, data coding, and	Table S1

For peer review only

		anonymisation / deidentification of excerpts	
Data analysis	#14	Process by which inferences, themes, etc. were identified and developed, including the researchers involved in data analysis; usually references a specific paradigm or approach; rationale	Table S1
Techniques to enhance trustworthiness	#15	Techniques to enhance trustworthiness and credibility of data analysis (e.g. member checking, audit trail, triangulation); rationale	Table S1
Syntheses and interpretation	#16	Main findings (e.g. interpretations, inferences, and themes); might include development of a theory or model, or integration with prior research or theory	8-17
Links to empirical data	#17	Evidence (e.g. quotes, field notes, text excerpts, photographs) to substantiate analytic findings	8-17<br>Tables S2 & S3
Intergration with prior work, implications, transferability and contribution(s) to the field	#18	Short summary of main findings; explanation of how findings and conclusions connect to, support, elaborate on, or challenge conclusions of earlier scholarship; discussion of scope of application / generalizability; identification of unique contributions(s) to scholarship in a discipline or field	8-9
Limitations	#19	Trustworthiness and limitations of findings	18
Conflicts of interest	#20	Potential sources of influence of perceived influence on study conduct and conclusions; how these were	19

For peer review only

		managed	
Funding	#21	Sources of funding and other support; role of funders in data collection, interpretation and reporting	19

The SRQR checklist is distributed with permission of Wolters Kluwer © 2014 by the Association of American Medical Colleges. This checklist can be completed online using https://www.goodreports.org/, a tool made by the EQUATOR Network in collaboration with Penelope.ai

For peer review only

BMJ Open

A qualitative investigation of relatives' and service users' experience of mental health care for suicidal behaviour in bipolar disorder.

Journal:	*BMJ Open*
Manuscript ID	bmjopen-2019-030335.R2
Article Type:	Original research
Date Submitted by the Author:	14-Oct-2019
Complete List of Authors:	Clements, Caroline; University of Manchester Faculty of Biology, Medicine and Health, Centre for Mental Health and Safety<br>Kapur, Navneet; University of Manchester Faculty of Biology, Medicine and Health, Centre for Mental Health and Safety<br>Jones, Steven; Lancaster University Faculty of Health and Medicine<br>Morriss, Richard; University of Nottingham, Psychiatry<br>Peters, Sarah; University of Manchester Faculty of Biology, Medicine and Health, Psychology and Mental Health
**Primary Subject Heading**:	Qualitative research
Secondary Subject Heading:	Mental health
Keywords:	suicide, self-harm, bipolar disorder, QUALITATIVE RESEARCH

SCHOLARONE™
Manuscripts

A qualitative investigation of relatives' and service users' experience of mental health care for suicidal behaviour in bipolar disorder.

Caroline Clements[1], N. Kapur[1&5], Steve Jones[2], Richard Morriss[3], and Sarah Peters[4]

[1]Centre for Mental Health and Safety, Division of Psychology and Mental Health, School of Health Sciences, University of Manchester, Manchester Academic Health Sciences Centre, UK.

[2]Spectrum Centre for Mental Health Research, School of Health and Medicine, Lancaster University, UK.

[3]Division of Psychiatry and Applied Psychology, School of Medicine, University of Nottingham, UK.

[4]Division of Psychology and Mental Health, School of Health Sciences, University of Manchester, UK.

[5]Greater Manchester Mental Health NHS Foundation Trust, Manchester, UK.

Corresponding Author:
Caroline Clements
Room 2.308, Jean McFarlane Building,
Centre for Mental Health and Safety,
The University of Manchester,
Oxford Road,
Manchester,
M13 9PL
UK
Fax: +44(0)161 275 0716
Email: caroline.v.clements@manchester.ac.uk

Word Count: 4907

Key Words: Suicide, self-harm, bipolar disorder, qualitative, relatives

For peer review only

For peer review only - http://bmjopen.bmj.com/site/about/guidelines.xhtml

ABSTRACT

Objective: People with bipolar disorder are known to be at high risk of engaging in suicidal behaviours, and those who die by suicide have often been in recent contact with mental health services. The objective of this study was to explore suicidal behaviour in bipolar disorder and how these are monitored and managed by mental health services.

Aims: Identify themes within relatives' and service users' accounts of mental health care, related to management and prevention of suicidal behaviour in bipolar disorder.

Design: Thematic analysis of 22 semi-structured interviews.

Participants: Participants were aged 18 years or over, fluent in written and spoken English, and either had bipolar disorder with a history of suicidal behaviour, or were relatives of people who had bipolar disorder and died by suicide.

Setting: England, UK.

Primary outcome: Themes identified from participants' accounts of mental health care for suicidal behaviours in bipolar disorder.

Results: Two main themes were identified. 'Access to care' was characterised by a series or cycle of potential barriers to care (e.g. gate-keepers, lack of an accurate diagnosis), which had the potential to increase risk of suicidal behaviour if failure to access care continued over time. 'Problems with communication' captured the importance of maintaining open routes of communication between all parties involved in care to ensure successful monitoring and management of suicidal behaviours in bipolar disorder.

Conclusions: Mental health services need to be accessible and respond rapidly to people with suicidal behaviour in bipolar disorder. Open communication and inclusion of relatives in care, where appropriate, could help closer monitoring of changes in symptoms that indicate increased risk.

For peer review only - http://bmjopen.bmj.com/site/about/guidelines.xhtml

For peer review only

Article Summary: Strengths and limitations of the study.

- This was a novel qualitative exploration of suicidal behaviour in bipolar disorder.
- Thematic analysis of in-depth semi-structured interviews generated rich and complex participant-driven data that offered new insight into why suicidal risk in people with bipolar disorder might be missed by mental health services.
- This study is exploratory in nature, and further work is needed to confirm which themes/subthemes are specific to people with bipolar disorder.

For peer review only

For peer review only - http://bmjopen.bmj.com/site/about/guidelines.xhtml

INTRODUCTION

Bipolar disorder is a common mental illness with an estimated prevalence over 2.4% worldwide [1]. People with bipolar disorder are subject to potential burdens and reduced quality of life across a number of domains (e.g. employment, social, physical health etc.), and are known to be at high risk of engaging in suicidal behaviours [2,3]. More than half of people diagnosed with bipolar disorder self-harm at least once in their life-time and self-harm is known to be a major risk factor for future suicide [4,5]. Indeed, risk of suicide in people with bipolar disorder has been estimated to be 20-30 times that of the general population [3,6–9]. Some authors suggest that failure to identify elevated risk of suicidal behaviours in people with bipolar disorder reflects the nature of the disorder with rapid changes in mood-state alongside impulsive characteristics leading to limitations in assessing diagnosis-specific risk [10,11].

A UK study of people with bipolar disorder who died by suicide while under the care of mental health services found that 60% had been seen by the care team within seven days of the suicide, and 40% were seen within 24 hours [12]. People with bipolar disorder who present to hospital following self-harm often report being currently under the care of community mental health services [13]. Such results suggest that risk in people with bipolar disorder is not being appropriately identified or responded to and therefore opportunities for intervention are being missed.

Treatments shown to be effective at reducing suicidal behaviour, such as cognitive-behavioural therapies or medication with lithium are perhaps not being successfully implemented by mental health services in response to suicidal crises in people with bipolar disorder [14–18]. However, work with people with other psychiatric diagnoses and general samples have revealed difficulties in access to and/or engagement with mental health services in relation to suicidal behaviours [19].

A number of studies have investigated barriers and facilitators of access to/engagement with mental health and medical services in general service user groups [20–22], people with psychiatric diagnoses [19], and after episodes of suicidal behaviour [23–25]. Qualitative methods have been used to explore the broader experiences of people with bipolar disorder (and their carers), such as social factors in suicidality [26], psychiatric assessment [27] and other aspects of mental health care [28–30]. A recent study by Vallarino et al (2019) explored the experiences of mental health care in people with bipolar disorder; timely provision of information about bipolar disorder, recovery-focused treatment, provision of psychosocial therapies, and the access to peer support, all had an impact on the attitudes to and level of engagement with care services [31]. To the best of our knowledge there has been no investigation specific to accessing care and support for suicidal

For peer review only - http://bmjopen.bmj.com/site/about/guidelines.xhtml

behaviours in people with bipolar disorder. Understanding the experiences of people with bipolar disorder with regard to how mental health services respond at times of crisis and increased risk is key to reducing suicidal behaviours. The aim of this study was to explore relatives' and service users' experience of mental health services in relation to suicidal behaviour in bipolar disorder and to look at why suicidal risk in this population might be missed.

METHOD

Participants

Participants were: 1) relatives/carers of people with bipolar disorder who died by suicide (hereafter referred to as 'relatives'), or 2) people with bipolar disorder who had a history of self-harm (hereafter referred to as 'service users': self-harm is defined here as any self-poisoning or self-injury without reference to motivation or level of suicidal intent [32]). To capture the broadest range of experiences possible inclusion criteria were limited to being over 18 years of age and fluent in written and spoken English (use of translators was beyond the scope of this study and fluency in English was essential enable fully informed consent). To maximize recruitment from this small and highly specific target population no time-limits were placed on when the suicidal behaviour had taken place.

Relatives and service users were included in order to encompass the full range of suicidal behaviour in bipolar disorder. While people with bipolar disorder who have experience of self-harm can speak to their own experiences, excluding information around those who died by suicide may neglect the experiences of a group who may have the highest need for intervention by mental health services. People bereaved by suicide are often included as informants in suicide research as they are well positioned to know details of the individual and circumstances prior to death by suicide unavailable from clinical records [33,34]. Although some significant events may be unknown to the bereaved person (as well as being subject to recall bias and the influence of distress on the over or under emphasis of salient factors) the inclusion of relatives is recognised as a pragmatic approach.

Given the sensitivity of the topic and potential for burden and distress, no formal assessments were conducted to establish a diagnosis of bipolar disorder. Inclusion was based on self-reported diagnosis, or diagnosis as reported by relatives. Interviews did cover diagnostic history and it was possible to confirm that 19 (all 11 service users and most who died by suicide) had received a formal diagnosis of bipolar disorder, while 3 had a provisional or suspected diagnosis made via a mental health professional prior to death. Service users' experience of self-harm included various behaviours, from medically minor self-injury to medically serious suicide attempts. Changes in

For peer review only - http://bmjopen.bmj.com/site/about/guidelines.xhtml

method of self-harm, severity and intent over time were common, which is consistent with other studies of self-harm [35].

Recruitment

Twenty-two people were recruited into the study, split equally between relatives and service users.

Recruiting from such a small and highly specific population was challenging and a variety of pragmatic methods were used. Recruitment initially took place in the North West area of England, but by necessity was expanded to include all of England (amendments were approved by the National Research Ethics Service to reflect these changes). Identification of potential participants was done using the following methods; identification of people who had presented to hospital following self-harm via local psychiatric liaison services in the City of Manchester; identification and referral via clinical studies officers based within the local NHS mental health trust; advertising via mental health charities and local newspapers; in-person visits to local voluntary sector support groups e.g. Bipolar UK; as well as personal referrals from other participants and research colleagues.

Potential participants were given a detailed participant information sheet and an opportunity to discuss the study further with the research team, before giving written informed consent to participate in the interview. Details of the study and procedure were repeated at the time of the interview and consent re-confirmed verbally.

Interviews

Semi-structured interviews were conducted using a topic guide to facilitate conversation. The topic guide was based on factors relevant to suicidal behaviour in bipolar disorder identified from existing research literature. Topics included; history of diagnosis, changes in mood or behaviour immediately before the most recent suicidal behaviour, situational factors such as negative life events or stressors, experience of mental health care, social support, and whether opportunities to prevent the suicidal behaviour had been missed. As this work was exploratory in nature, participants were encouraged to tell the story of their experiences in their own way and encouraged to raise any additional issues they felt were important. Two authors (SP and CC) developed the interview procedure with input from the other authors, and one (CC) conducted the interviews. An outline of the sorts of topics that might be discussed was presented at the start of the interview to ensure that the participant was comfortable with the topics, and the semi-structured nature of the interview was explained to ensure that participants knew they were in control of the conversation and could discuss any topics they thought important to the experience of suicidal behaviour in bipolar disorder.

For peer review only - http://bmjopen.bmj.com/site/about/guidelines.xhtml

Participants were interviewed in-person at the participant's home (n=10), or by telephone (n=12), ensuring the participant had appropriate levels of comfort and privacy in which to talk. Interviews were audio-recorded and transcribed verbatim. A code was allocated to each interview and identifying information (e.g. names and places) was removed to maintain anonymity. Interview duration ranged from 30 to 90 minutes, with a mean duration of 1 hour. The use of both interview methods was a pragmatic decision to aid recruitment and thereby access a range of perspectives and experiences. There are potential advantages and disadvantages to using both telephone and face-to-face interviews. Interviews via telephone can add distance and detachment during a potentially sensitive interview and help people feel more comfortable disclosing personal information [36,37]; however, face-to-face methods are known to increase rapport in the interview setting and may help put participants at ease [38,39]. There was no discernible differences identified between the telephone and face-to-face interviews in topics covered or themes identified in the analysis.

Service user and Public involvement

This work was carried out as part of the PARADES programme of research into bipolar disorder. A service user reference group for the programme had input into the development of the project from an early stage. Methodology and recruitment materials were reviewed by the group and amendments made in line with feedback. A first draft of the topic guide was confirmed to be generally suitable for the purpose of the project, but the group advised that more detail be collected on past history of self-harm and interaction with services prior to the most recent episode of suicidal behaviour to give additional context to participants' experiences. The interview was also piloted with a service user who confirmed the interview procedure and topic guide were comprehensive and appropriate for the research topic.

Analysis

Thematic analysis was used on the interview data [40]. As this method is unattached to a specific theoretical framework and offers flexibility to explore and interpret detailed qualitative data, it is particularly suited to pragmatic and exploratory work.

Detailed information on the analytic procedure is presented in Supplementary Table S1. To summarize, initial coding was conducted by CC, transcripts were read by another CC and SP who to confirm concordance of coding and ideas during analysis. Data were given preliminary codes which were organised within a text document. An iterative process of discussion and review between CC and SP consolidated codes into potential themes (these codes were grouped together in the working

For peer review only - http://bmjopen.bmj.com/site/about/guidelines.xhtml

document). Themes were summarised and discussed with the extended research team to refine final themes. Conceptual models depicting possible relationships between subthemes were developed during analysis to aid understanding of each themes and how subthemes may be linked. The typical progression or sequence of events over time as described by participants were noted and used to help construct these models, which were reviewed and amended by the research team as analysis progressed.

Steps taken to ensure the trustworthiness of the analysis included a multidisciplinary team that comprised qualitative methodologists, clinical psychologists, psychiatrists and expert suicide and self-harm researchers. Throughout the study the team were reflexive, using individual reflexive notes and group discussions. This involved considering their own personal experiences and positions and how these may impact on the data generation, interpretation and final analysis. In doing so the researchers did not aspire to objectivity but to understand the subjectivity inherent in the process of qualitative research [41].

Ethical approval

Ethical approval for the study was obtained from the National Research Ethics Service (Ref: 10/H1003/84) and R&D approvals were granted from all trusts involved in recruitment.

RESULTS

A summary of participants' age and gender are provided in Table 1.

TABLE ONE HERE

Analysis of interview data identified two main themes; 'access to care' and 'problems with communication'. Themes and subthemes are presented below. For the purpose of brevity abridged quotes (presented in *italics*) by relatives [r] and service users [s] are presented. Full quotations and additional supporting quotes are provided in Supplementary Table S2 and Supplementary Table S3.

1. Access to care.

Participants reported a range of difficulties when trying access to mental health services when risk of engaging in suicidal behaviour was high. This applied across different service-contact situations, such as outpatient care, people discharged or disengaged from services, as well as people trying to access care for the first time. Within this the subthemes 'help-seeking,' 'knowing how to navigate the system,' 'gatekeepers,' 'obtaining a correct diagnosis', and 'increased risk due to being denied care,'

For peer review only - http://bmjopen.bmj.com/site/about/guidelines.xhtml

formed a series of potential barriers to accessing successful mental health care for suicidal behaviours in bipolar disorder. While barriers could be experienced as single 'one off' events, many accounts reported a sequence of events where one barrier led to the next and so on, until access to services was achieved.

Help-Seeking: Most accounts showed that efforts were made to actively seek help for suicidal behaviours from health/mental health services, particularly at times when suicidal thoughts or behaviours increased.

> *"He'd put himself in hospital, he hadn't been sectioned or anything, because he just felt so suicidal and depressed [r1]."*

Knowing how to navigate the system: Participants reported difficulties navigating access to appropriate mental health services. There was confusion over what services were available, which would provide the right support for people experiencing suicidal thoughts and behaviours, and how to initiate contact based on current patient-status (e.g. service provision differed depending on whether they were a current, discharged, or new patient). At times of suicidal crisis, when urgent help was needed, knowing how/who to contact for effective support was seen as vital—however, services did not always respond promptly.

> *"We didn't realise that we had a pass [...] back into the mental health system so we went back to the GP [r6]."*

> *"I went to see the doctor and she said […] I am going to get someone to ring you from the crisis team [...]. Do you know how long it took? They never phoned me five weeks. I suffered like that for five weeks. [s7]."*

Confusion over what services was not restricted to service users/relatives, other medical professionals such as GPs also struggled when navigating the mental health care system.

> *"[T]he GP had phoned up the psychiatrist's secretary as well, and had got exactly the same. It was ring five numbers on a piece of paper, she said phone them so I phoned them, and they gave me another number so I phoned them [...] and it went round in a circle, so in the end the fifth person gave me the number of the first person […] and my GP got given the same ring of numbers and went round the same way [s5]."*

There were also difficulties experienced when trying to access additional support services (unavailable on the NHS or only available via long waiting-lists – such as talking therapies). Where participants were willing and able (e.g. financially) to tap into additional sources of mental health support there was a wish expressed for guidance and signposting by the existing care-team. This type of guidance was either limited or refused, leaving service users/relatives frustrated.

For peer review only - http://bmjopen.bmj.com/site/about/guidelines.xhtml

> *"I said can I short circuit it by paying for it and she sort of said 'I'm not allowed to recommend anyone.' So it was this, I'm trying to do the best for [the service user] and you won't help me [r2]."*

Gate-keepers: The first point of contact during times of increased risk of suicidal behaviour was often the GP, who then acted as a gate-keeper for accessing secondary mental health services. In this role GPs could be facilitators or barriers to access.

> *"I cannot fault my GP, because they […] could not have dealt with it any better [r3]."*

Gate-keepers' poor attitudes and/or lack of knowledge about how to respond to people with bipolar disorder experiencing suicidal thoughts and behaviours were a common feature in participants' accounts.

> *"I have to say the GPs were very much, 'oh pull yourself together,' you know, sort of attitude [r6]."*

Hospital emergency departments were another critical point of contact and gate-keeping of access to mental health care. Participants were often subject to the negative attitudes of medical staff responsible for their treatment, and for referring those who present with suicidal behaviours to psychiatric teams. Psychiatric staff were also seen as gate-keepers of access to appropriate care, demonstrated by failures to recognise bipolar disorder symptomatology and the associated increased risk of suicidal behaviours in this population.

> *"I was as honest as I am being with you, and [psychiatric liaison clinician] just turned to [psychiatric liaison nurse] and he went, 'do you believe a word she's saying?' And this woman just looked at him and went, 'no' and I felt about that big, and I'm sort of thinking I need help… help [s1]."*

While the quotes above describe first attempts to access mental health care, gate-keepers were also a potential barrier to ongoing successful care for those already in contact with secondary mental health services. For example, when the psychiatrist responsible for a service user's care is unavailable at a time of crisis:

> *"[S]o there was another psychiatrist at the hospital, they wouldn't see me because I was under the care of [consultant psychiatrist]. So they wouldn't touch me [s5]."*

Obtaining a correct diagnosis: A common experience among participants was the failure of clinicians to recognise a diagnosis of bipolar disorder. By failing to take account of the individual's diagnosis clinicians failed to correctly evaluate increased risk of suicidal behaviour. Lack of a formal diagnosis also impacted on access to care and increased the risk of inappropriate or inadequate medication for

For peer review only - http://bmjopen.bmj.com/site/about/guidelines.xhtml

those already in contact with services, with the potential to exacerbated symptoms and increase suicidal behaviours.

> *"[T]hey put me on antidepressants and I went really, really manic for seven months or something [...]. So I was just left on these antidepressants going higher and higher [s5]."*

Increased risk due to being denied care: The final subtheme represents the vulnerability of participants to the emotional and illness-related toll of repeated failures to access care (e.g. when failing to overcome the barriers presented in previous subthemes). Participants were clear that this had potential to become a cyclic pattern of repeated attempts and failures to access care, with the attendant disappointment and frustration precipitating further suicidal thoughts and behaviours; leaving participants feeling like they have to *"scream for help [s2]."*

> *"The crisis team, the home treatment team, nobody would see me, and that is, that is when this [suicidal behaviour] happened, really I was just really desperate [s5].*

There was also distress at often not being seen as 'ill enough' to deserve mental health care. This was particularly evident when service users looked well or were able to articulate concerns about their illness and the risk of engaging in suicidal behaviours (it is worth noting the interview sample in this study generally had a high level of educational attainment which may be related to the ability to better describe symptoms and distress).

> *"I find with services they get quite confused you know they look at me and say, 'You are not depressed, you are bathed and clothed.' I am terribly depressed even though I am bathed and clothed [s3]."*

Barriers to accessing care were evident throughout the data, but a minority of participants reported positive experiences whereby access to care for the prevention of suicidal behaviours was facilitated by gatekeepers and/or knowledge of which service to contact in a crisis. Therefore, barriers discussed here only have the potential to interrupt or block access to care.

A model of relationships between subthemes is provided in Figure 1 to aid in interpretation of the 'access to care' theme. 'Help-seeking' is represented as the first step in the model. This connects directly to issues around 'knowing how to navigate the system,' as correct knowledge may facilitate access to care but it may also act as a barrier. This subtheme is then linked to 'gatekeepers' and on to 'obtaining a correct diagnosis.' Another failure at this point leads into the theme of 'increased risk due to being denied care.' The circular path of the arrows, denoting relationships between subthemes, represents the potential for individuals to become trapped in a cycle, this is only one interpretation and barriers may be encountered in any order and access to care maybe achieved at any point.

For peer review only - http://bmjopen.bmj.com/site/about/guidelines.xhtml

2. Problems with communication.

This theme describes the importance of clear communication in the ongoing care of people with bipolar disorder, especially at times of crisis when suicidal behaviour is likely. Paths of communication are between any parties with an interest in the care of a service user, and are here presented in relation to the ‘service user’, ‘relatives’, and the ‘mental health team’.

Service users: Service users were the main source of information about changes in illness, behaviour, and risk of suicidal behaviour. Participants acknowledged that suicidal intent or increases in suicidal thoughts and behaviour were not always disclosed. As a consequence carers and services remained awareness of changes in symptoms and behaviours that might indicate increased risk (e.g. non-fatal suicidal behaviour) remained hidden, and opportunities to intervene were lost.

> *“I didn’t want a professional to know. It’s classic hiding it and not telling anybody, because they have to intervene [s2].”*

Failure to disclosure information that might help manage suicidal behaviour was not always intentional on behalf of the service user. Participants gave examples of memory problems and disorganised lifestyles (e.g. substance use problems, or lack of a permanent address) as possible reasons why important changes may not be reported.

> *“I used to get quite cross […], he would say he hadn't seen [his community psychiatric nurse], but when I inquired into it, the more likely thing was that they had gone to his house and he wasn't there [r8].”*

Motivation for failing to communicate suicidal thoughts and behaviours was driven largely by the wish to protect others from distress and to avoid placing additional burden on relatives.

> *“[S]he didn't want to worry us [r7].”*

> *“I keep a lot to myself and I don't know why I just, I just feel as though if I say things I will upset people [s6].”*

The tangible consequence of this was that relatives and/or the mental health team were unaware of increasing risk and unable to respond effectively.

> *“I came down and found him in the chair. He took an overdose and he’d promised [the psychiatrist] that he wasn’t suicidal. [r1].”*

Relatives: The involvement of relatives in mental health care was generally viewed as positive. Proximity to the service user helped with observation of changes in illness and behaviour. Relatives were also privy to changes in situational factors, such as the presence of stressful or negative life

For peer review only - http://bmjopen.bmj.com/site/about/guidelines.xhtml

events that could increase the risk of suicidal behaviour, and which could then be communicated to the care team.

> *"[W]e were aware that he was drinking. […] I brought all this to the attention of his CPN […] and he was quite shocked by that, and the living conditions, how [the service user] was living [r4]."*

Relatives saw themselves as an untapped resource for services, and wanted to be considered and treated, as full partners in the care of the service user.

> *"[A]s a family we were their biggest resource and they never sort of came to us to help. Either to help us, or to use what we were saying to help him [r4]."*

Not all service users felt supported by relatives or wanted the support of relatives in the management of their illness or suicidal behaviours. For a couple of participants there was resistance on the part of the family to being involved in care. In these cases a lack of knowledge of bipolar disorder and the associated risks of suicidal behaviour were cited as obstacles to engaging family members in the care of the service user. Where family involvement was wanted, mental health services were viewed as a source of engagement and education for relatives, and there was a view that working collaboratively would improve support and safety for the service user at home.

> *"I would like somebody to come here and sit them all down and tell them what it involves because I don't think they have a clue [s7]."*

Where familial relationships were poor the involvement of family members was seen as potentially detrimental to the service user and their ongoing care.

> *"[T]the services would be so grateful to have family involved that they push the care to the family where really that is detrimental to me [s3]"*

Participants also gave accounts of conflict between family members and mental health staff. This was particularly evident when relatives felt prevented from contributing to care—which often went hand-in-hand with confusion over confidentiality rules.

> *"You just got told on many, many occasions that, you know, its patient confidentiality. […] I turn round and said to them, I said "if my son had cancer, you would have involved me, to care for him quite well at home [r4]."*

This led to failures in communicating information that had potential value for assessing risk. Relatives expressed frustration at being unable to help the service user despite being in possession of knowledge that may have influenced care.

For peer review only - http://bmjopen.bmj.com/site/about/guidelines.xhtml

"[W]hen he died, you know, the psychiatrist had actually said that he didn't think it was planned suicide, […] even though he had been telling me that he wanted, you know, he didn't want to live. He wanted to die for probably months [r6]."

Mental health teams: Participants gave multiple accounts of communication failures between service users and the care team. Poor explanations of diagnosis and prognosis, risks and triggers, medication regimens and possible side effects of treatments, all contributed to service users feeling unheard by clinical teams. This resulted in poorer management, weaker therapeutic relationships, and ultimately the potential to miss indicators of increased risk.

"[T]hey never said to me, you know your mom has mental illness you might need to you know go and get yourself checked out at the doctors, with your suicidal thoughts, suicidal tendencies... [s4]."

Following episodes of suicidal behaviour, some participants reported being left without any follow-up or acknowledgment from clinical teams. Subsequently there was no adjustment of treatment, additional interventions, or changes in monitoring in response to the suicidal behaviour.

"[O]ne of the home treatment team came into the appointment and sat in the room with me, and neither she nor the psychiatrist mentioned the fact that I had self-harmed and run away [s5]."

There was however evidence that communication between services and service users has improved over time. With accounts of new teams and initiatives created to better ensure the safety of service users, and overall participants were hopeful that services would continue to improve.

"[S]he came out on the Friday and brought the crisis team out […] they said if I needed them over the weekend that they could you know, I could ring them and they would come and see me. So you have a bigger support work now than you did a few years ago [s4]."

The theme 'problems with communication' encapsulates many of the vulnerabilities in routes of communication between service users, relatives, and mental health services, in the provision of care for people with bipolar disorder and suicidal behaviour. It is interesting to note that the converse of this was also true; participants' judgements of the quality of care received were more positive in cases where the care team were seen as collaborative and inclusive. Where communication between parties was good opinions on the quality of care received were positive regardless of outcome.

"[T]there was one guy, he was one of the few [health care professionals] that both [the service user] and I trusted because he was quite happy to have us both in at the same time [r2]."

For peer review only - http://bmjopen.bmj.com/site/about/guidelines.xhtml

Sympathy and trust were key features of positive evaluations of care. Clinicians who were willing to provide and receive information from service users and relatives were valued. Continuity of care to establish long-term therapeutic relationships were also vital for building trust, and allowed clinicians greater understanding of service users' illness, behaviour, and situation; aiding monitoring of bipolar disorder symptoms and identification of times of increased risk of suicidal behaviour. Where these factors were lacking, participants evaluated care as poor.

> *"I gave up [on] the psychiatrist because they were a bloody waste of time, they didn't know me. [...] They just read the notes and there was no connection at all [s7]."*

A model of subthemes and vulnerabilities in communication is provided in Figure 2 to aid interpretation of the 'problem with communication' theme. 'Service users,' 'relatives,' and 'mental health teams' are connected to each other and communication can flow in any direction. When communication is open and positive care is viewed as more collaborative and satisfaction with the care team is higher. Examples of problems in communication are given outside the pathways of communication with arrows denoting the possibility that any of these issues could interrupt communication at any time, and impact on the care of the service user.

DISCUSSION

This exploratory study used a qualitative approach to investigate experiences of mental health care in relation to suicidal behaviour in bipolar disorder. Two main themes captured the focus of participants' accounts; access to care, and problems in communication, both of which may have contributed to failures to identify and respond to increased risk of suicide in bipolar disorder.

A number of subthemes identified were consistent with experiences common among people with other psychiatric diagnoses [21,22]. While this does not diminish the importance of these concerns, their impact on the individual, or the possibility these difficulties may be over-represented among people with bipolar disorder, the focus here is on the identification of novel factors that may be specific to care for suicidal behaviours in bipolar disorder.

Active help-seeking was common with the majority of participants recognising the need for increased support when experiencing increased suicidal thoughts and behaviours. Barriers were predominantly external, such as gate-keeping and poor staff attitudes to suicidal behaviours [42,43]. There was no evidence of issues relating to stigma or other individual-centred barriers as documented in more general studies of people with bipolar disorder [21,22]. However, it is possible

For peer review only - http://bmjopen.bmj.com/site/about/guidelines.xhtml

that internalised stigma may have played a role in the decision not to disclose suicidal thoughts to relatives or care teams, which would fall under the theme ‘problems with communication’ [44].

One aspect of gate-keeping that may be particularly relevant people with bipolar disorder was the assumption of low illness severity (and therefore low risk of suicidal behaviours) based on superficial aspects of participants’ appearance and the ability to effectively communicate concerns and distress. Bipolar disorder is characterised by periods of illness and relative stability when individuals may be better able to effectively recognise and communicate distress and risk [3]. Compared to people with other psychiatric diagnoses, people with bipolar disorder often have a higher educational attainment level, similar to that of the general population [45,46]. Although it was not included as a subtheme, the participants in the current study were particularly well educated—many at degree level or higher—perhaps suggesting this sample had more effective communication skills, especially when describing risk of suicidal behaviours.

Ensuring accurate diagnosis was critical in the management of suicidal behaviours in bipolar disorder. Compared to other psychiatric diagnoses, a diagnosis of bipolar disorder is often delayed due to misdiagnosis of initial symptoms especially when symptoms of depression are present [47]. The experiences of participants who received inappropriate treatments and the exacerbated symptoms and increased risk of suicidal behaviours that followed, emphasised the importance of increasing clinician knowledge, especially for those who may be a first point of contact such as GPs and psychiatric liaison staff, in recognising bipolar disorder and the risks associated with this illness.

Mental health care for suicidal behaviour was enhanced by involving service users and relatives (where appropriate) in care decisions and resulted in positive evaluations of care even after a death by suicide. Work by Owen et al (2015) on the impact of social factors in suicidal thoughts showed that ‘not being understood or acknowledged,’ and ‘feeling burdensome’ triggered or exacerbated suicidal thoughts and acts [26]. Participants’ accounts of problems in communication in the current work echo these results, especially in terms of non-disclosure of suicidal intent to avoid burden on family members. However, participants often wanted the support of family, and the involvement of family in care decisions was highly valued. Therapies that involve family members (e.g. family-focused therapy, psychoeducation) have been shown to help reduce relapse, symptom severity and caregiver burden and may be effective in addressing communication blocks identified in this study. This may be especially true of therapies that seek to educate service users and families by increasing knowledge bipolar disorder and associated increased risks [48,49]. Recent work examining the experiences of mental health care by people with bipolar disorder showed that meaningful information about bipolar disorder and its treatment (as well as psychosocial interventions and

For peer review only - http://bmjopen.bmj.com/site/about/guidelines.xhtml

access to peer support) resulted in more positive attitudes towards care and better engagement with services [31].

Previous qualitative work showed that people with serious mental illness struggle to be seen as competent and equal partners in care decisions [50] and this was evident in the current work where the focus was specifically on help for suicidal behaviours. These results also support the idea that relatives and carers can feel excluded from mental health care [30]. As in general mental health care for people with bipolar disorder, care received for suicidal behaviour was viewed more positively when care was seen as collaborative and clinicians were sympathetic, open, and interested in involving service users and relatives [51].

Methodological limitations

Total number of participants was small but sufficient to provide a novel insight into the experiences of a small and hard to reach population via rich in-depth data. Our model arose from views of service users and relatives who are both key stakeholders who provide valuable perspectives on the experiences. The relatives' cohort had a high degree of information power on the subject of service responses to suicide as all had been bereaved by suicide [52]. The service user sample was more heterogeneous with a wide range of experience of suicide thoughts, feelings and behaviours and the final model was supported by data from both sets of participants. Given the heterogeneity of the service user group, further research may be needed to ensure all experiences are accurately captured. Themes and conceptual models presented in this work do not attempt to give an exhaustive account of all experiences of accessing mental health care by people with suicidal behaviours in bipolar disorder. However, identification of factors considered important by participants with diverse perspectives and experiences have been described, and common difficulties faced by this high-risk group when trying to access care have been successfully identified.

Clinical implications

Many people who experience suicidal behaviour in bipolar disorder are in close proximity to care services. While the influence of rapidly changing mood-states and impulsivity make prediction and prevention of suicidal behaviours in this group particularly difficult, the experience of people with suicidal behaviours and bipolar disorder may have a role in helping to improve the response of mental health services [53–55]. Rapid access to care is crucial, and service users and relatives need good knowledge of what services are available to them, alongside clearly signposted ways to access these services. Relatives are often in a position to observe behavioural changes that may indicate increased or fluctuating risk, and where possible, should be kept informed of

For peer review only - http://bmjopen.bmj.com/site/about/guidelines.xhtml

treatment/management plans. Closer involvement of family and friends may allow important information to be communicated more quickly, help build trust and supportive therapeutic relationships, and create more positive judgements of overall quality of care provided by mental health services [56].

There may also be a role for improvement in health professionals' knowledge of bipolar disorder and the increased risk of suicide conferred by this illness. Participants' accounts consistently showed some level of underestimation of illness severity and suicide risk by clinicians, even for individuals under mental health care. A combination of greater knowledge of bipolar disorder in general and increased communication skills in the identification of suicidal thoughts and behaviours may be beneficial.

Additional interview work with clinicians may help uncover problems in communication about suicidal behaviour in people with bipolar disorder, and help explore the perception of lower risk in people who are better able to communicate their distress and concerns. Further work with a wider range of participants, particularly those from less educated backgrounds where additional barriers may include health literacy, is needed to explore the generalisability of the themes presented as well as how themes might be translated into improved care practices.

Author contributions: NK and CC designed the study with input from all authors. CC collected all data. CC and SP analysed and interpreted the data, with input from all authors. CC drafted the manuscript. NK, SP, RM, SJ provided further input on subsequent drafts of the manuscript.

Funding: This article presents independent research commissioned by the National Institute for Health Research (NIHR) under its Programme Grants for Applied Research funding scheme (RP-PG-0407-10389). The views expressed in this work are those of the authors and not those of the NHS, NIHR, or Department of Health.

Competing interests: None

Patient consent for publication: Not required

Data statement: Due to confidentiality full interview data cannot be made available.

For peer review only - http://bmjopen.bmj.com/site/about/guidelines.xhtml

REFERENCES

1 Merikangas KR, Jin R, He J, *et al.* Prevalence and correlates of bipolar spectrum disorder in the world mental health survey initiative. *Arch Gen Psychiatry* 2011;**68**:241–51. doi:10.1001/archgenpsychiatry.2011.12.Prevalence

2 Michalak EE, Murray G, Collaborative RESearch Team to Study Psychosocial Issues in Bipolar Disorder (CREST.BD). Development of the QoL.BD: a disorder-specific scale to assess quality of life in bipolar disorder. *Bipolar Disord* 2010;**12**:727–40. doi:10.1111/j.1399-5618.2010.00865.x

3 Goodwin FK, Jamison KR. *Manic-Depressive Illness:Bipolar Disorders and Recurrent Depression: Bipolar Disorders and Recurrent Depression*. Oxford University Press, USA 2007. http://books.google.co.uk/books?id=X7XLhltqsv8C

4 Hawton K, Zahl D, Weatherall R. Suicide following deliberate self-harm : long-term follow-up of patients who presented to a general hospital Suicide following deliberate self-harm : long-term follow-up of patients who presented to a general hospital. *Br J Psychiatry* 2003;**182**:537–42. doi:10.1192/02-540

5 Carroll R, Metcalfe C, Gunnell D. Hospital presenting self-harm and risk of fatal and non- fatal repetition: Systematic review and meta-analysis. *PLoS One* 2014;**9**. doi:10.1371/journal.pone.0089944

6 Schaffer A, Isometsä ET, Tondo L, *et al.* International Society for Bipolar Disorders Task Force on Suicide: Meta-analyses and meta-regression of correlates of suicide attempts and suicide deaths in bipolar disorder. *Bipolar Disord* 2015;**17**:1–16. doi:10.1111/bdi.12271

7 Tondo L, Pompili M, Forte A, *et al.* Suicide attempts in bipolar disorders: Comprehensive review of 101 reports. *Acta Psychiatr Scand* 2016;**133**:174–86. doi:10.1111/acps.12517

8 Pompili M, Gonda X, Serafini G, *et al.* Epidemiology of suicide in bipolar disorders: a systematic review of the literature. *Bipolar Disord* 2013;**15**:457–90. doi:10.1111/bdi.12087

9 Nordentoft M, Mortensen PBB, Pedersen CB. Absolute risk of suicide after first hospital contact in mental disorder. *Arch Gen Psychiatry* 2011;**68**:1058–64. doi:10.1001/archgenpsychiatry.2011.113

10 Watkins HB, Meyer TD. Is there an empirical link between impulsivity and suicidality in

For peer review only - http://bmjopen.bmj.com/site/about/guidelines.xhtml

bipolar disorders? A review of the current literature and the potential psychological implications of the relationship. *Bipolar Disord* 2013;**15**:542–58. doi:10.1111/bdi.12090

11 Gonda X, Pompili M, Serafini G, *et al.* Suicidal behavior in bipolar disorder: epidemiology, characteristics and major risk factors. *J Affect Disord* 2012;**143**:16–26. doi:10.1016/j.jad.2012.04.041

12 Clements C, Morriss R, Jones S, *et al.* Suicide in bipolar disorder in a national English sample, 1996-2009: Frequency, trends and characteristics. *Psychol Med* 2013;**43**. doi:10.1017/S0033291713000329

13 Clements C, Jones S, Morriss R, *et al.* Self-harm in bipolar disorder: Findings from a prospective clinical database. *J Affect Disord* 2015;**173**:113–9. doi:10.1016/j.jad.2014.10.012

14 Smith KA, Cipriani A. Lithium and suicide in mood disorders: Updated meta-review of the scientific literature. *Bipolar Disord* 2017;**19**:575–86. doi:10.1111/bdi.12543

15 Baldessarini RJ, Tondo L, Vázquez GH. Pharmacological treatment of adult bipolar disorder. *Mol Psychiatry* 2018;:1–20. doi:10.1038/s41380-018-0044-2

16 Tarrier N, Taylor K, Gooding P. Cognitive-behavioral interventions to reduce suicide behavior: a systematic review and meta-analysis. *Behav Modif* 2008;**32**:77–108. doi:10.1177/0145445507304728

17 Rihmer Z, Gonda X, Tondo L. Lithium and suicidal behavior in patients with bipolar disorder. *J Clin Psychiatry* 2008;**69**:1831–2.http://www.ncbi.nlm.nih.gov/pubmed/19200432 (accessed 12 May 2014).

18 Cipriani A, Hawton K, Stockton S, *et al.* Lithium in the prevention of suicide in mood disorders: updated systematic review and meta-analysis. *BMJ* 2013;**3646**:1–13. doi:10.1136/bmj.f3646

19 Anderson KK, Fuhrer R, Malla AK. “There are too many steps before you get to where you need to be”: Help-seeking by patients with first-episode psychosis. *J Ment Heal* 2012;**22**:384–95. doi:10.3109/09638237.2012.705922

20 Smith TE, Easter A, Pollock M, *et al.* Disengagement From Care: Perspectives of Individuals With Serious Mental Illness and of Service Providers. *Psychiatr Serv* 2013;**64**:770–5. doi:10.1176/appi.ps.201200394

For peer review only

For peer review only - http://bmjopen.bmj.com/site/about/guidelines.xhtml

21 Clement S, Schauman O, Graham T, *et al.* What is the impact of mental health-related stigma on help-seeking? A systematic review of quantitative and qualitative studies. *Psychol Med* 2015;**45**:11–27. doi:10.1017/S0033291714000129

22 Dockery L, Jeffery D, Schauman O, *et al.* Stigma- and non-stigma-related treatment barriers to mental healthcare reported by service users and caregivers. *Psychiatry Res* 2015;**228**:612–9. doi:10.1016/j.psychres.2015.05.044

23 Owens C, Lambert H, Donovan J, *et al.* A qualitative study of help seeking and primary care consultation prior to suicide. *Br J Gen Pract* 2005;**55**:503–9.

24 Taylor TL, Hawton K, Fortune S, *et al.* Attitudes towards clinical services among people who self-harm: Systematic review. *Br J Psychiatry* 2009;**194**:104–10. doi:10.1192/bjp.bp.107.046425

25 Jordan J, McKenna H, Keeney S, *et al.* Providing meaningful care: learning from the experiences of suicidal young men. *Qual Heal Res* 2012;**22**:1207–19. doi:10.1177/1049732312450367

26 Owen R, Gooding P, Dempsey R, *et al.* A qualitative investigation into the relationships between social factors and suicidal thoughts and acts experienced by people with a bipolar disorder diagnosis. *J Affect Disord* 2015;**176**:133–40. doi:10.1016/j.jad.2015.02.002

27 Bilderbeck AC, Saunders KEA, Price J, *et al.* Psychiatric assessment of mood instability: qualitative study of patient experience. *Br J Psychiatry* 2014;**204**:234–9. doi:10.1192/bjp.bp.113.128348

28 Highet N, McNair B, Thompson M, *et al.* Experience with treatment services for people with bipolar disorder. *Med J Aust* 2004;**181**:47–51.https://www.mja.com.au/journal/2004/181/7/experience-treatment-services-people-bipolar-disorder

29 Chatzidamianos G, Lobban F, Jones S. A qualitative analysis of relatives', health professionals' and service users' views on the involvement in care of relatives in Bipolar Disorder. *BMC Psychiatry* 2015;**15**:228. doi:10.1186/s12888-015-0611-x

30 Cree L, Brooks HL, Berzins K, *et al.* Carers ' experiences of involvement in care planning : a qualitative exploration of the facilitators and barriers to engagement with mental health

For peer review only

For peer review only - http://bmjopen.bmj.com/site/about/guidelines.xhtml

services. *BMC Psychiatry* 2015;:1–11. doi:10.1186/s12888-015-0590-y

31 Vallarino M, Rapisarda F, Scott J, *et al.* Experiences of Mental Healthcare Reported by Individuals Diagnosed with Bipolar Disorder: An Italian Qualitative Study. *Community Ment Health J* 2019;**55**:129–36. doi:10.1007/s10597-018-0311-3

32 Hawton K, Harriss L, Hall S, *et al.* Deliberate self-harm in Oxford, 1990-2000: a time of change in patient characteristics. *Psychol Med* 2003;**33**:987–95.http://www.ncbi.nlm.nih.gov/pubmed/12946083 (accessed 26 May 2014).

33 Isometsä ET. Psychological autopsy studies--a review. *Eur Psychiatry* 2001;**16**:379–85.http://www.ncbi.nlm.nih.gov/pubmed/11728849

34 Sher L. Psychological autopsy studies: Past, present, and future. *Aust New Zeal J Psychiatry* 2013;**47**:884–884. doi:10.1177/0004867413479071

35 Owens D, Kelley R, Munyombwe T, *et al.* Switching methods of self-harm at repeat episodes: Findings from a multicentre cohort study. *J Affect Disord* 2015;**180**:44–51. doi:10.1016/j.jad.2015.03.051

36 Trier-Bieniek a. Framing the telephone interview as a participant-centred tool for qualitative research: a methodological discussion. *Qual Res* 2012;**12**:630–44. doi:10.1177/1468794112439005

37 Sturges J, Hanrahan K. Comparing telephone and face-to-face qualitative interviewing: a research note. *Qual Res* 2004;**4**:107–18.

38 Shuy R. In-person versus telephone interviewing. In: Gubrium J, Holstein J, eds. *Handbook of Interview Research: context and method*. Thousand Oaks, CA: : Sage 2001. 536–54. doi:10.4135/9781412973588

39 Creswell J. *Qualitative inquiry and research design: choosing between five approaches*. Third Edit. Thousand Oaks, CA: : Sage 2012.

40 Braun V, Clarke V. Using thematic analysis in psychology. *Qual Res Psychol* 2006;**3**:77–101. doi:10.1191/1478088706qp063oa

41 Peters S. Qualitative research methods in mental health. *Evid Based Ment Health* 2010;**13**:35–40. doi:10.1136/ebmh.13.2.35

For peer review only

42 Anthony JS, Baik RNS, Bowers RNBJ, *et al.* Conditions That Influence a Primary Care Clinician ' s Decision to Refer Patients for Depression Care. *Rehabil Nurs* 2010;**35**:113–22.

43 Saunders KEA, Hawton K, Fortune S, *et al.* Attitudes and knowledge of clinical staff regarding people who self-harm: A systematic review. *J Affect Disord* 2012;**139**:205–16. doi:10.1016/j.jad.2011.08.024

44 Hawke LD, Parikh S V, Michalak EE. Stigma and bipolar disorder: a review of the literature. *J Affect Disord* 2013;**150**:181–91. doi:10.1016/j.jad.2013.05.030

45 Schoeyen HK, Melle I, Sundet K, *et al.* Occupational outcome in bipolar disorder is not predicted by premorbid functioning and intelligence. *Bipolar Disord* 2013;**15**:294–305. doi:10.1111/bdi.12056

46 Dickinson D, Harvey PD. Systemic hypotheses for generalized cognitive deficits in schizophrenia: a new take on an old problem. *Schizophr Bull* 2009;**35**:403–14. doi:10.1093/schbul/sbn097

47 Altamura AC, Buoli M, Caldiroli A, *et al.* Misdiagnosis, duration of untreated illness (DUI) and outcome in bipolar patients with psychotic symptoms: A naturalistic study. *J Affect Disord* 2015;**182**:70–5. doi:10.1016/j.jad.2015.04.024

48 Miklowitz DJ, Chung B. Family-Focused Therapy for Bipolar Disorder: Reflections on 30 Years of Research. *Fam Process* 2016;**55**:483–99. doi:10.1111/famp.12237

49 Baruch E, Pistrang N, Barker C. Psychological interventions for caregivers of people with bipolar disorder: A systematic review and meta-analysis. *J Affect Disord* 2018;**236**:187–98. doi:10.1016/j.jad.2018.04.077

50 Dahlqvist Jönsson P, Schön U-K, Rosenberg D, *et al.* Service users' experiences of participation in decision making in mental health services. *J Psychiatr Ment Health Nurs* 2015;**22**:688–97. doi:10.1111/jpm.12246

51 Norcross JC, Wampold BE. Evidence-based therapy relationships: research conclusions and clinical practices. *Psychotherapy (Chic)* 2011;**48**:98–102. doi:10.1037/a0022161

52 Malterud K, Siersma VD, Guassora AD. Sample Size in Qualitative Interview Studies: Guided by Information Power. *Qual Health Res* 2016;**26**:1753–60. doi:10.1177/1049732315617444

For peer review only - http://bmjopen.bmj.com/site/about/guidelines.xhtml

53 Cassidy F. Risk factors of attempted suicide in bipolar disorder. *Suicide Life Threat Behav* 2011;**41**:6–11. doi:10.1111/j.1943-278X.2010.00007.x

54 D'Ambrosio V, Salvi V, Bogetto F, *et al.* Serum lipids, metabolic syndrome and lifetime suicide attempts in patients with bipolar disorder. *Prog Neuropsychopharmacol Biol Psychiatry* 2012;**37**:136–40. doi:10.1016/j.pnpbp.2011.12.009

55 Tondo L, Baldessarini R. Suicidal risk in bipolar disorder. *Clin Neuropsychiatry* 2005;**2**:55–65.

56 Dirik A, Sandhu S, Giacco D, *et al.* Why involve families in acute mental healthcare? A collaborative conceptual review. *BMJ Open* 2017;**7**:1–10. doi:10.1136/bmjopen-2017-017680

For peer review only

Table 1. Gender and age of interview participants

**Variable**	**Participants**<br><br>**n=22(%)**
**Sex**	
Male	4 (18.2)
Female	18 (81.8)
**Age group**	
18-35	4 (18.2)
36-50	10 (45.5)
51-65	4 (18.2)
66>	4 (18.2)

For peer review only

Figure 1: Model of subthemes within the main theme 'access to care'.

Figure 2: Model of subthemes within the main theme 'problems with communication'.

For peer review only

For peer review only - http://bmjopen.bmj.com/site/about/guidelines.xhtml

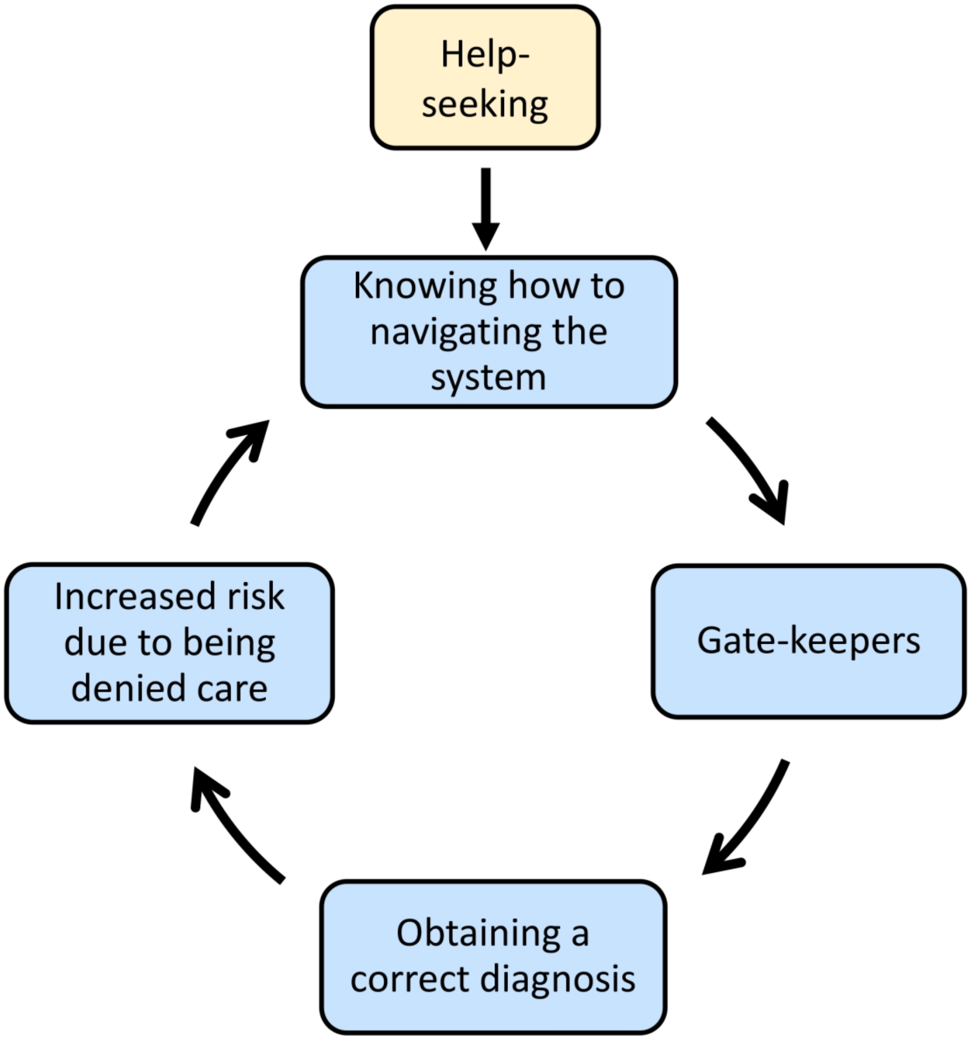

Figure 1. Model of subthemes within the main theme 'access to care'.

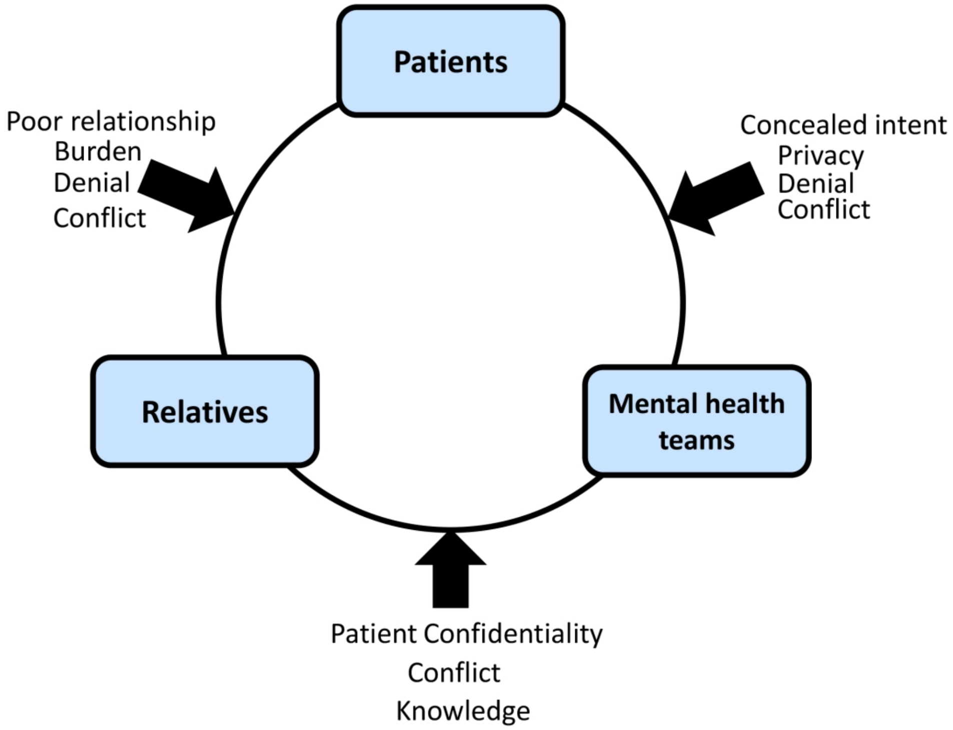

Figure 2. Model of subthemes within the main theme 'problems with communication'.

Supplementary Table S1: Account of the method used to identify themes, adapted from Braun and Clarke (2006).

Familiarisation with data corpus.<br>Analyses started when two relatives and two self-harm participants' interviews had been completed and transcribed in full. Close reading of the transcripts by CC and SP enabled familiarisation with the data. Interviews and analysis were conducted in parallel and additional transcripts were incorporated into the analysis as they became available. Back-coding of older transcripts was carried out when new codes were identified.
Generation of initial codes.<br>Coding was conducted by CC and SP in order to confirm concordance of coding and ideas during analysis. Interesting features within the participants' accounts were highlighted and allocated preliminary codes. These coded extracts were compiled and organised within a working text document. Examples of early codes included: 'family fighting for additional care,' 'changes in personality not recorded by services,' 'hiding suicidal behaviour,' 'not wanting to be a burden to family.'
Identification of themes.<br>Through an iterative process of discussion and review CC and SP consolidated codes into potential themes by grouping coded extracts that represented the same or similar issues within the working document. While the accounts were initially analyses as a single group (of people with experience of suicidal behaviours in bipolar disorder) at this point attention was given to whether themes were consistent or discriminant between the accounts of the two participant populations (i.e. people with bipolar and suicidal behaviour disorder and people bereaved by the suicide of someone with bipolar disorder). Analysis continued until thematic saturation was achieved, and no new ideas relating to the research topic emerged.
Review and definition of themes.<br>Themes identified were summarised and presented for discussion with the extended multidisciplinary research team at team meetings. Themes were presented along with supporting quotes from across the participants group. Themes were accepted as valid once there was unanimous agreement across the research team that the theme was present and accurately captured the experiences of participants.
Reporting the analysis.<br>Appropriate supporting quotes were selected from participants' account for inclusion in the report to illustrate each theme and associated subthemes. Models (Figures 1 and 2) were constructed to help describe the relationship between subthemes and aid understanding. Models were confirmed as appropriate via review and discussion with the research team. Results were then contextualized with reference to existing literature on care for suicidal behaviour in bipolar disorder.

For peer review only

Supplementary Table S2: Full quotes relating to the theme 'access to care.'

Help-seeking
"He'd put himself in hospital, he hadn't been sectioned or anything, because he just felt so suicidal and depressed [r1]."
"I want to feel better, I don't want to be feeling low and you know, not happy [s6]."
Knowing how to navigate the system
"We didn't realise that we had a pass, if you like, back into the mental health system so we went back to the GP [r6]."
"I went to see the doctor and she said to me, right […] I am going to get someone to ring you from the crisis team, and you need somebody to talk to. Do you know how long it took? They never phoned me five weeks. I suffered like that for five weeks. It took five weeks, and in the end I have to phone up, and then they had no recognition of who I was [s7]."
"[T]he GP had phoned up the psychiatrist's secretary as well, and had got exactly the same. It was ring five numbers on a piece of paper, she said phone them so I phoned them, and they gave me another number so I phoned them, and they gave me another number so I phoned them, and they gave me another number, and it went round in a circle, so in the end the fifth person gave me the number of the first person to phone so I had been round in a ring […]and my GP got given the same ring of numbers and went round the same way [s5]"
"I said can I short circuit it by paying for it and she sort of said 'I'm not allowed to recommend anyone.' So it was this, I'm trying to do the best for [the patient] and you won't help me [r2]."
Gate-keepers
"I cannot fault my GP, because they […] could not have dealt with it any better. He was wonderful, and got us into the psychiatric hospital as soon, the same day, which was just unbelievable [r3]."
"I have to say the GPs were very much, 'oh pull yourself together,' you know, sort of attitude [r6]."

For peer review only

"I spent 3 days in and out of A&E begging them to help me [s2]."
"I was as honest as I am being with you, and [psychiatric liaison clinician] just turned to [psychiatric liaison nurse] and he went, 'do you believe a word she's saying?' And this woman just looked at him and went, 'no' and I felt about that big, and I'm sort of thinking I need help… help [s1]"
"Nobody else would see me, so there was another psychiatrist at the hospital, they wouldn't see me because I was under the care of [consultant psychiatrist]. So they wouldn't touch me, so... it just shows how you can fall out of the system really [s5]."
Obtaining a correct diagnosis
"[T]hey put me on antidepressants and I went really, really manic for seven months or something, and I ended up just leaving. Oh, at the hospital I got arrested because I threw a bin through a window. And they had me arrested and then refused to treat me, which is where I was getting the treatment. So I was just left on these antidepressants [without a mood stabiliser] *going higher and higher [s5]."*
Increased risk due to being denied care
"The crisis team, the home treatment team, nobody would see me, and that is, that is when this [suicidal behaviour] happened, really I was just really desperate [s5].
"And also I present always with a mask and I think that's why I was diagnosed so late. You know because I am always, and I find with services they get quite confused you know they look at me and say, 'You are not depressed, you are bathed and clothed.' I am terribly depressed even though I am bathed and clothed [s3]."
"It used to make him angry, cross, you know, and he couldn't get people to sort of listen, and to help him [r8]."

For peer review only

Supplementary Table S3: Full quotes supporting the theme "problems with communication".

Service users
"She was putting up this front that she was absolutely fine, and that was the really big message that was coming across [r5]."
"I didn't want a professional to know. It's classic hiding it and not telling anybody, because they have to intervene [s2]."
"I had a suicide plan in place, or whatever they call it, and wouldn't reveal the details of that to him [the psychiatrist] *[s5]"*
"I used to get quite cross because, you know, he would say he hadn't seen this person [community psychiatric nurse], but when I inquired into it, the more likely thing was that they had gone to his house and he wasn't there [r8]."
"She had attempted it maybe about four times, but she didn't want us to know, she didn't want to worry us [r7]."
"I have got like a really good family, but I find it difficult to let people know a lot of stuff, they do know, but I keep a lot to myself and I don't know why I just, I just feel as though if I say things I will upset people [s6]."
"I came down and found him in the chair. He took an overdose and he'd promised [the psychiatrist] that he wasn't suicidal. He said that he was alright now things were getting done [r1]."
Relatives
"[W]e were aware that he was drinking. I brought all this, about a month before [the patient] died, I brought all this to the attention of his CPN […] and he was quite shocked by that, and the living conditions, how [the patient] was living [r4]."
"I had been delusional for weeks and weeks and I was really poorly, and my daughter was ringing the mental health service saying you need to come and check my mum she is not, my

For peer review only

mum is not well she is acting in a weird way, she is walking round naked, she is not good, you need to come you know, and they didn't come [s4].”
“[A]s a family we were their biggest resource and they never sort of came to us to help. Either to help us, or to use what we were saying to help him [r4].”
“I feel so sorry for anybody who had got depression or anything like that got no chance unless you have got somebody to help you [s7].”
“I would like somebody to come here and sit them all down and tell them what it involves because I don't think they have a clue [s7].”
“I have written an advanced statement, because you know the services would be so grateful to have family involved that they push the care to the family, where really that is detrimental to me [s3]”
“You just got told on many, many occasions that, you know, its patient confidentiality. But, when you’re dealing with somebody who’s suffering from mental health, and if I turn round and said to them, I said “if my son had cancer, you would have involved me, to care for him quite well at home [r4].”
“[W]hen he died, you know, the psychiatrist had actually said that he didn't think it was planned suicide, because he didn't think [the patient] was in that frame of mind to make that decision at that time, even though, even though he had been telling me that he wanted, you know, he didn't want to live. He wanted to die for probably months [r6].”
“[W]hen he died, you know, the psychiatrist had actually said that he didn't think it was planned suicide, because he didn't think [the patient] was in that frame of mind to make that decision at that time, even though, even though he had been telling me that he wanted, you know, he didn't want to live. He wanted to die for probably months [r6].”
Mental health teams
“[T]hey never said to me, you know your mom has mental illness you might need to you know go and get yourself checked out at the doctors, with your suicidal thoughts, suicidal tendencies... they didn't do it, they didn't do it [s4].”

For peer review only

"[I]n the appointment that I had, after I had self-harmed and run away, [...] one of the home treatment team came into the appointment and sat in the room with me, and neither she nor the psychiatrist mentioned the fact that I had self-harmed and run away [s5]."
"[W]hen I spoke to [community psychiatric nurse] on the Thursday after me thinking about suicide on the Wednesday, she spoke to the psychiatrist and then she spoke to the crisis team, and she came out on the Friday and brought the crisis team out on the Friday and said, and they said if I needed them over the weekend that they could you know, I could ring them and they would come and see me. So you have a bigger support work now than you did a few years ago, because there wasn't no crisis team a few years ago [s4]."
"I gave up [on] *the psychiatrist because they were a bloody waste of time, they didn't know me. They didn't know who I was. They just read the notes and there was no connection at all [s7]."*

For peer review only

Reporting checklist for qualitative study.

Based on the SRQR guidelines.

Instructions to authors

Complete this checklist by entering the page numbers from your manuscript where readers will find each of the items listed below.

Your article may not currently address all the items on the checklist. Please modify your text to include the missing information. If you are certain that an item does not apply, please write "n/a" and provide a short explanation.

Upload your completed checklist as an extra file when you submit to a journal.

In your methods section, say that you used the SRQR reporting guidelines, and cite them as:

O'Brien BC, Harris IB, Beckman TJ, Reed DA, Cook DA. Standards for reporting qualitative research: a synthesis of recommendations. Acad Med. 2014;89(9):1245-1251.

	Reporting Item	Page Number
#1	Concise description of the nature and topic of the study identifying the study as qualitative or indicating the approach (e.g. ethnography, grounded theory) or data collection methods (e.g. interview, focus group) is recommended	2
#2	Summary of the key elements of the study using the abstract format of the intended publication; typically	2

For peer review only

		includes background, purpose, methods, results and conclusions	
Problem formulation	#3	Description and signifcance of the problem / phenomenon studied: review of relevant theory and empirical work; problem statement	4/5
Purpose or research question	#4	Purpose of the study and specific objectives or questions	5
Qualitative approach and research paradigm	#5	Qualitative approach (e.g. ethnography, grounded theory, case study, phenomenolgy, narrative research) and guiding theory if appropriate; identifying the research paradigm (e.g. postpositivist, constructivist / interpretivist) is also recommended; rationale. The rationale should briefly discuss the justification for choosing that theory, approach, method or technique rather than other options available; the assumptions and limitations implicit in those choices and how those choices influence study conclusions and transferability. As appropriate the rationale for several items might be discussed together.	5-7
Researcher characteristics and reflexivity	#6	Researchers' characteristics that may influence the research, including personal attributes, qualifications / experience, relationship with participants, assumptions and / or presuppositions; potential or actual interaction between researchers' characteristics and the research questions, approach, methods, results and / or transferability	6

For peer review only

Context	#7	Setting / site and salient contextual factors; rationale	6-7
Sampling strategy	#8	How and why research participants, documents, or events were selected; criteria for deciding when no further sampling was necessary (e.g. sampling saturation); rationale	6-7
Ethical issues pertaining to human subjects	#9	6-7	4
Data collection methods	#10	Types of data collected; details of data collection procedures including (as appropriate) start and stop dates of data collection and analysis, iterative process, triangulation of sources / methods, and modification of procedures in response to evolving study findings; rationale	6-7
Data collection instruments and technologies	#11	Description of instruments (e.g. interview guides, questionnaires) and devices (e.g. audio recorders) used for data collection; if / how the instruments(s) changed over the course of the study	6-7
Units of study	#12	Number and relevant characteristics of participants, documents, or events included in the study; level of participation (could be reported in results)	6-7
Data processing	#13	Methods for processing data prior to and during analysis, including transcription, data entry, data management and security, verification of data integrity, data coding, and	Table S1

For peer review only

		anonymisation / deidentification of excerpts	
Data analysis	#14	Process by which inferences, themes, etc. were identified and developed, including the researchers involved in data analysis; usually references a specific paradigm or approach; rationale	Table S1
Techniques to enhance trustworthiness	#15	Techniques to enhance trustworthiness and credibility of data analysis (e.g. member checking, audit trail, triangulation); rationale	Table S1
Syntheses and interpretation	#16	Main findings (e.g. interpretations, inferences, and themes); might include development of a theory or model, or integration with prior research or theory	8-17
Links to empirical data	#17	Evidence (e.g. quotes, field notes, text excerpts, photographs) to substantiate analytic findings	8-17 Tables S2 & S3
Intergration with prior work, implications, transferability and contribution(s) to the field	#18	Short summary of main findings; explanation of how findings and conclusions connect to, support, elaborate on, or challenge conclusions of earlier scholarship; discussion of scope of application / generalizability; identification of unique contributions(s) to scholarship in a discipline or field	8-9
Limitations	#19	Trustworthiness and limitations of findings	18
Conflicts of interest	#20	Potential sources of influence of perceived influence on study conduct and conclusions; how these were	19

For peer review only

		managed	
Funding	#21	Sources of funding and other support; role of funders in data collection, interpretation and reporting	19

The SRQR checklist is distributed with permission of Wolters Kluwer © 2014 by the Association of American Medical Colleges. This checklist can be completed online using https://www.goodreports.org/, a tool made by the EQUATOR Network in collaboration with Penelope.ai

For peer review only
